# Development of Kilogram-Scale Electrochemical Ni-Catalyzed Cross Electrophile Coupling in Flow

**DOI:** 10.1021/acs.oprd.6c00110

**Published:** 2026-06-16

**Authors:** Megan Kelly, Luana Cardinale, Karrin V. Sackett, Suqi Zhang, Gregory L. Beutner, Benjamin Cohen, Shannon S. Stahl, Marcel Schreier

**Affiliations:** † Department of Chemical and Biological Engineering, 5228 University of Wisconsin-Madison, Madison, Wisconsin 53706, United States; ‡ Department of Chemistry, 5228 University of Wisconsin-Madison, Madison, Wisconsin 53706, United States; § Chemical Process Development, Bristol Myers Squibb, 1 Squibb Drive, New Brunswick, New Jersey 08903, United States

**Keywords:** electrochemistry, nickel, homogeneous catalysis, flow chemistry

## Abstract

Organic electrosynthesis opens novel routes for pharmaceutical production; however, limited knowledge and experience with the scale up of this technique have hindered its industrial implementation. In this work, we report a kilogram-scale flow electrochemical Ni-catalyzed cross-electrophile coupling (XEC) to prepare a key intermediate in the synthesis of the toll-like receptor (TLR) 7/8 inhibitor afimetoran. We successfully demonstrate scalability across 2 orders of magnitude while maintaining reaction performance. We also conduct a continuous 120 h reaction, highlighting compatibility with industrially relevant time scales. Key design aspects of our custom parallel-plate reactor are discussed that enabled kilogram-scale synthesis with a single electrode stack. This report showcases that with proper reaction engineering, electrochemical Ni-catalyzed XEC is a viable alternative to existing Pd-catalyzed cross-coupling methods.

## Graphical abstract

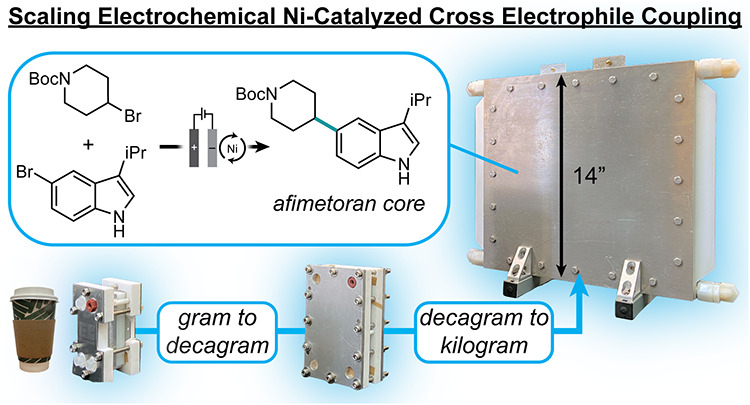

## Introduction

Electrochemistry is a powerful tool that can unlock new routes for challenging active pharmaceutical ingredient (API) syntheses.^1–8^ However, industrial implementation of organic electrochemistry will require scaling these pathways well beyond the multigram scales commonly employed in academic laboratories.^1^ Demonstrations exceeding 100 g are uncommon,^1,10–23^ and kilogram-scale examples are even fewer. To our knowledge, since 2000, only 6 literature examples report organic electrosyntheses targeting pharmaceutical production at the kilogram scale (Figure 1a).^22,24–28^ Achieving this scale of operation is a critical stepping-stone in moving from exploratory medicinal chemistry to implementation in production processes. Furthermore, despite growing interest in reductive electrosynthetic pathways,^7,29–35^ only one out of the six recent kilogram-scale examples was a reductive reaction, published in 2002.^25^

**Figure 1:**
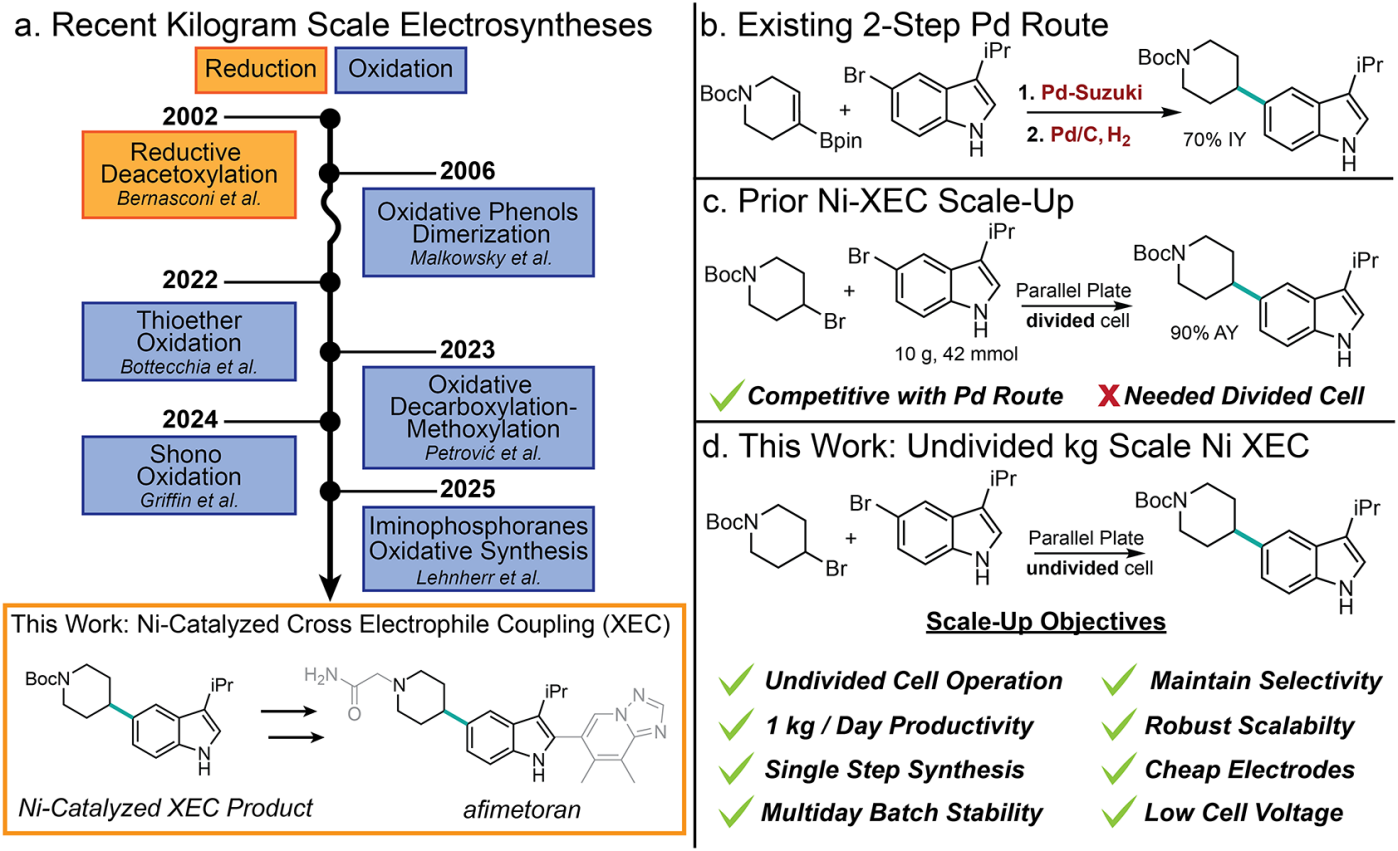
(a) Reported kilogram-scale electrosyntheses since 2000.^22,24–28^ (b) Existing Pd-catalyzed route to the afimetoran intermediate **3**, IY = isolated yield. (c) Prior scale-up of this Ni-catalyzed XEC reaction completed at the decagram scale in a divided parallel plate flow cell, AY = assay yield.^40^ (d) The objective of this work, featuring Ni-catalyzed XEC performed at the kilogram scale in an undivided parallel plate flow cell.

For electrochemistry to become an accepted and routine industrial technique, five key factors have been identified that an ideal electrochemical process must meet: safety, environmental friendliness, high productivity, cost efficiency, and process robustness.^1^ Ni-catalyzed cross-electrophile coupling (XEC) is a synthetic pathway that has the potential to satisfy the aforementioned criteria for an ideal large-scale electrochemical process. Cross-coupling reactions are ubiquitous in API production, and many rely on palladium-catalyzed technology.^31,35^ However, given the rising cost and scarcity of Pd, moving to more earth-abundant transition metals such as nickel could provide significant environmental and economic benefits.^36,37^ Furthermore, Ni-catalyzed XEC can be electrochemically conducted at ambient temperatures and pressures on inexpensive electrodes.^30,38^ However, previous investigations into electrochemical Ni-catalyzed XEC revealed challenges with maintaining reaction selectivity, as homodimer and proto-dehalogenated side products can form in significant quantities, reducing yield and impacting product purity.^40^ Ni-catalyzed XEC was previously identified as a possible alternative to the existing two-step Pd-catalyzed synthesis of a key intermediate for the toll-like receptor (TLR) 7/8 antagonist afimetoran (Figure 1b).^41^ A 10 g scale electrosynthesis was successfully completed in 2024,^40^ but achieving high yields required the use of a divided cell (Figure 1c). While these findings provided key insights into the drivers needed to maintain high product selectivity, the complexity of using a separated cell raised concerns about the viability of moving to larger scales.

Herein, we describe research toward the electrochemical Ni-catalyzed XEC synthesis of the afimetoran core **3** with a specific focus on (1) maximizing product yield in an undivided reactor, (2) increasing the applied current to minimize reactor size, (3) demonstrating multiday stability, and (4) completing a kilogram-scale synthesis without sacrificing reaction performance. We demonstrated that electrochemical Ni-catalyzed XEC can run at the kilogram scale in an undivided reactor while maintaining the selectivity seen at smaller scales. In addition to operating at the kilogram scale, we achieved an 87% in-process product yield after a 120 h electrolysis at the 100 mmol scale (Figure 1d), demonstrating compatibility with industrially relevant batch lengths. On the basis of these findings, we can project requirements for a theoretical production campaign, including estimated reactor footprint, power requirements, batch lengths, and safety considerations, informing a data-driven assessment of the true viability of organic electrosynthesis in large-scale pharmaceutical development.

## Results

### Initial Optimization in a Batch-Mode Undivided Cell

The first objective of our study was to optimize the reaction conditions for an undivided batch cell. Prior studies indicated that operating in a divided cell provided higher product selectivity and yield;^40^ however, implementing a membrane at scale presents design and stability challenges.^1,42,43^ Thus, we sought to optimize the product yield obtainable in a small undivided batch cell at the 0.42 mmol scale (Figure S8). Previously optimized literature conditions yielded 71% of product **3** in an undivided batch configuration using dimethylacetamide (DMA) as the solvent.^40^ We began by testing more benign solvents, such as acetonitrile, propylene carbonate, and 2-butanone, as alternatives to DMA. However, in all cases, the reaction performance was drastically lowered, leading to a mixture of side products and unconverted starting material (entries 2–4, Table 1). We then explored the use of sacrificial reductants as an alternative to using sacrificial Zn metal rods, given the previously identified deleterious role of Zn­(II) ions on reaction selectivity.^40^ However, neither γ-terpinene nor DIPEA (diisopropylethylamine)^44^ were tolerated under the reaction conditions, with both leading to very low conversions of starting material **2**. Next, we evaluated the effect of increased current densities on reaction performance (entries 7–8, Table 1). Generally, higher current densities led to lower conversions, which were likely due to catalyst deactivation. Finally, we increased the electrolyte concentration to 0.4 M, which led to 86% of **3**, comparable to yields previously obtained in a divided cell (90%).^40^ This result confirmed that high product yields could be achieved in an undivided cell configuration.

**Table 1:**
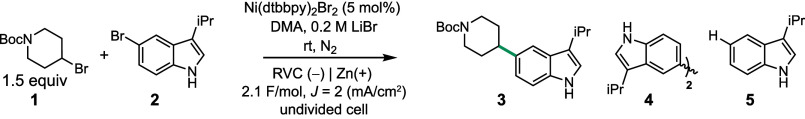
Results from Initial Optimization Scheme in Undivided Batch Cell

**Entry**	**Deviations from std**	**2 (%)** ^a^	**3 (%)** ^a^	**4 (%)** ^a^	**5 (%)** ^a^
1	None	0	71	3	28
2	MeCN instead of DMA	48	35	0	0
3	Propylene carbonate instead of DMA	49	16	7	26
4	2-butanone instead of DMA	50	0	0	50
5	γ-terpinene (4 equiv)^b^	90	5	0	0
6	DIPEA (4 equiv)^b^	83	20	0	0
7	J = 4 mA cm^–2^	52	39	0	0
8	J = 5 mA/cm^–2^	58	33	0	0
9	**LiBr 0.4 M**	**0**	**86**	**0**	**7**

a Yields were reported from ^1^H NMR spectroscopy with 1,3,5-trimethoxybenzene as an external standard.

b Graphite was used as the anode.

### Increasing Production Rate

Our results in the undivided batch cell revealed that the Ni-catalyzed XEC reaction was highly sensitive to the current density. Maximizing the applied current is critical to increasing productivity while minimizing the reactor size. Thus, we sought to determine the highest current density that could be applied without sacrificing the reaction performance. With the eventual target of achieving a kilogram-scale synthesis, we transitioned from a 0.42 mmol scale in a batch reactor to batch sizes between 20 and 80 mmol in a parallel plate reactor with a geometric electrode area of 86 cm^2^ (Figure 2) run in a flow-recirculation configuration. To compare productivity across different reaction conditions, we used a nominal current density metric^27^ normalized to the 2D geometric area of the electrode (Figure S3). Normalizing to the 2D geometric area of the electrode also allowed us to compare the reaction performance between different thicknesses of carbon felt despite the difference in 3D surface area.

**Figure 2:**
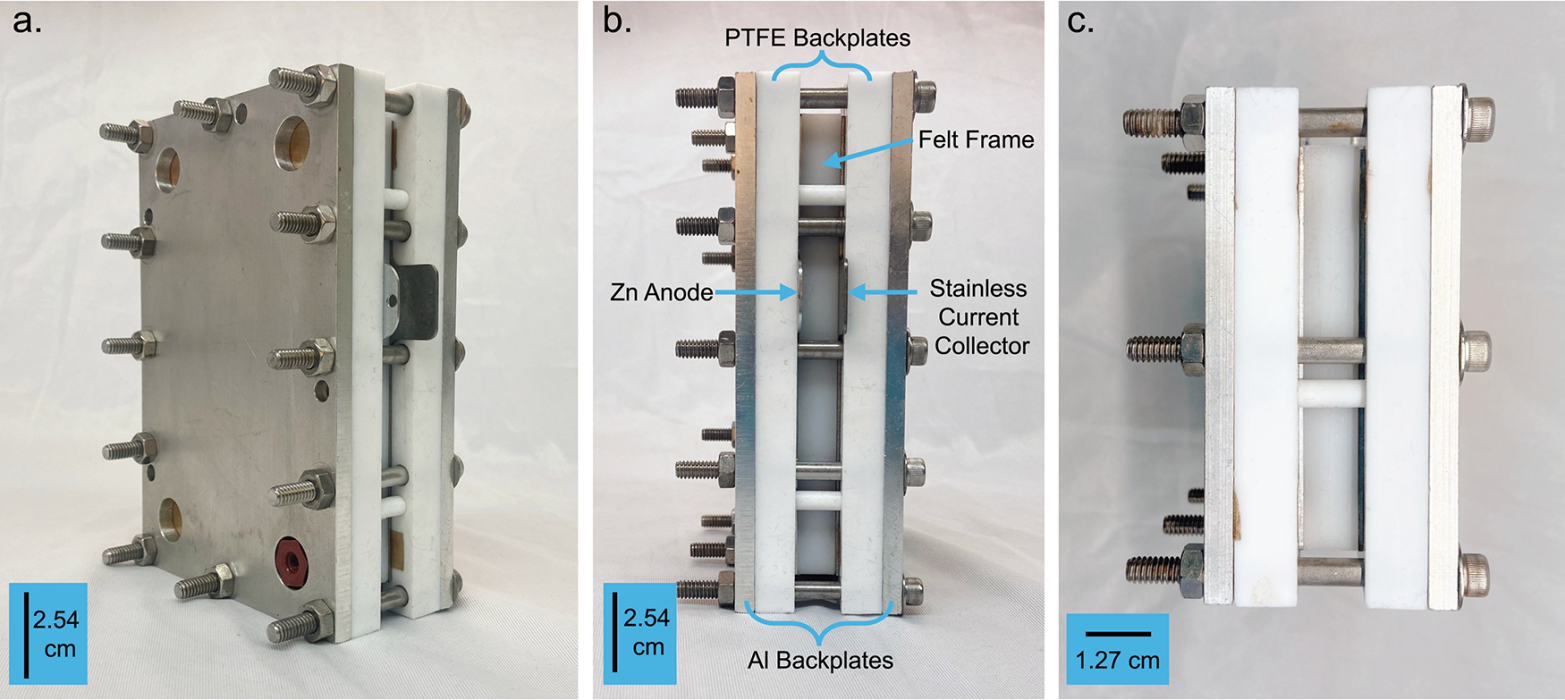
(a) In-house designed and fabricated standard 86 cm^2^ reactor. Tabs on side are connection points for the electrical leads. (b) Side profile of reactor with the layers of the reactor labeled. (c) Overhead view of reactor, felt frame is 1.27 cm thick.

We hypothesized that two parameters could be varied to increase the applied current: the starting material concentration and electrode surface area. Despite the 2D geometric area for the cathode being fixed at 86 cm^2^ for our reactor design, we circumvented this limitation in surface area by using a high-surface-area 3D carbon felt cathode on a current collector instead of a graphite plate. As shown in Table 2, the material identity of the current collector was insignificant, with comparable performance between resin-filled graphite and stainless steel (SST) (entries 1–6). However, stainless steel is preferable from a cost and structural perspective compared to resin-filled graphite.

**Table 2:**
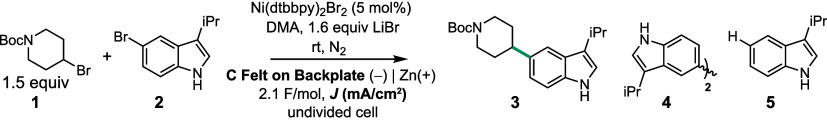
Optimization of Reaction Parameters to Maximize Production Rate in 86 cm^2^ Reactor

Entry	Carbon felt Thickness (cm)	Carbon felt Backplate Material	[**2**] (M)	Mass **2** (g)	*J* (mA cm^–2^)	**2 (%)** ^a^	**3 (%)** ^a^	**4 (%)** ^a^	**5 (%)** ^a^
1	0.635	Resin Filled Graphite	0.25	4.73	5.5 (1 F mol^–1^) to 3.1 (1.1 F mol^–1^)	0	89	6	8
2	0.635	Stainless Steel	0.25	4.73	5.5 (1 F mol^–1^) to 3.1 (1.1 F mol^–1^)	1	89	4	8
3	0.635	Resin Filled Graphite	0.5	9.46	11 (1 F mol^–1^) to 6.3 (1.1 F mol^–1^)	2	83	10	4
4	0.635	Stainless Steel	0.5	9.46	11 (1 F mol^–1^) to 6.3 (1.1 F mol^–1^)	7	81	9	3
5	0.635	Resin Filled Graphite	0.5	9.46	9	0	83	9	4
6	0.635	Stainless Steel	0.5	9.46	9	1	86	9	4
7	0.635	Stainless Steel	0.8^b^	18.92	15	23	54	14	3
8	0.635	Stainless Steel	1^b^	18.92	12	21	54	20	3
9	1.27	Stainless Steel	0.3	9.46	10	3	82	8	3
10	1.27	Stainless Steel	0.3	9.46	12	29	63	2	1
11	1.27	Stainless Steel	0.4	11.35	12	20	75	6	3
**12**	**1.27**	**Stainless Steel**	**0.5**	1**4.19**	**12**	**8**	**84**	**6**	**3**
13	1.27	Stainless Steel	0.75^b^	21.28	15	23	60	8	3

a Yields reported from ^1^H NMR spectroscopy with 1,3,5-trimethoxybenzene as an external standard.

b The solubility limit of LiBr was reached at 0.8 M; thus, these concentrations did not feature 1.6 equiv of LiBr.

We began our flow experiments using 0.635 cm thick carbon felt with 0.25 M **2**, the concentration previously tolerated in flow conditions.^40^ We assessed the impact of operating under a multistep current profile that applied a high current density for the first 1 F mol^–1^ of charge passed, then decreased the current density to prevent catalyst deactivation for the remaining 1.1 F mol^–1^ of charge. With 0.25 M of compound **2**, this current profile was 5.5 mA cm^–2^ stepped to 3.1 mA cm^–2^ — corresponding to an average current density of 4.2 mA cm^–2^. As shown in entry 2 in Table 2, this profile allowed for an 89% yield of **3** at a current density slightly higher than the 4 mA cm^–2^ electrolysis in the batch cell which did not achieve high conversion of **2**. We next studied the impact of changing the concentration of **2** with this multistep current profile. Doubling the concentration of **2** to 0.5 M indeed allowed for the current density profile to also be doubled (11 mA cm^–2^ stepped to 6.3 mA cm^–2^, entry 4, Table 2). This profile corresponded to an average current density of 8.5 mA cm^–2^. However, 0.5 M **2** could tolerate a constant current density of 9 mA cm^–2^ without a significant change in the yield of **3** (entry 6 Table 2). Based on these results, all further studies used a constant current density for operational simplicity. The solubility limit was reached at 1.0 M of **2**. However, concentrations above 0.5 M **2** led to a decrease in conversion of **2** and a simultaneous undesirable increase in homodimer **4** generation regardless of current density (entries 7–8, Table 2). Thus, 0.5 M **2** was determined to be the optimal concentration.

In addition to increasing the concentration of **2**, we also increased the felt thickness. Using a thicker felt increases the overall surface area, which we postulated should allow for an overall higher current to be applied. Moving from a 0.635 cm (0.25”) to a 1.27 cm (0.5”) thick carbon felt allowed the nominal current to be increased from 9 mA cm^–2^ to 12 mA cm^–2^ with 0.5 M **2**. An attempt to run at a higher current density of 15 mA cm^–2^ and a higher concentration of 0.75 M **2** on the 1.27 cm thick carbon felt resulted in incomplete conversion of **2**, likely due to catalyst deactivation (entry 13, Table 2). Thus, we determined 12 mA cm^–2^ to be the maximum current density that could be applied on the 1.27 cm felt, even at an increased concentration of **2**.

### Long-Term Stability

The next step of our investigation was to evaluate the long-term stability of the reaction in the context of a multiday electrolysis for a production campaign. In order to extend the length of the run while maintaining a similar batch size, we employed a small parallel plate reactor with a geometric electrode area of 4 cm^2^ (Figure S1). The Zn anode thickness was increased to 1.27 cm (0.5”) to provide ample tolerance for the expected change in anode thickness of approximately 2 mm (see Supporting Information for details on calculation) due to the oxidation of Zn. We translated our optimized conditions from Table 2 with 0.5 M **2** and 12 mA cm^–2^ to the small reactor. As shown in entry 1 of Table 3, the reaction was intended to run for 117.3 h. However, at 71 h, a solid began to precipitate (Figures S15, S16). While the solid did not adversely affect product formation, it caused an increase in cell voltage that necessitated termination of the experiment after 94.3 h, resulting in only 1.7 F mol^–1^ of charge being passed. Attempts to handle the solid via in-line filtration were unsuccessful due to filter clogging; thus, we sought to understand the origin of its formation and prevent precipitation. Analysis of the isolated solids (Figure S17, Table S2) confirmed that the precipitated solid was the homodimer **6** formed from the excess piperidine **1** (Table 3). This homodimer **6** has been reported to form during the cross-coupling reaction;^31^ however, its precipitation under these conditions was unexpected.

**Table 3:**
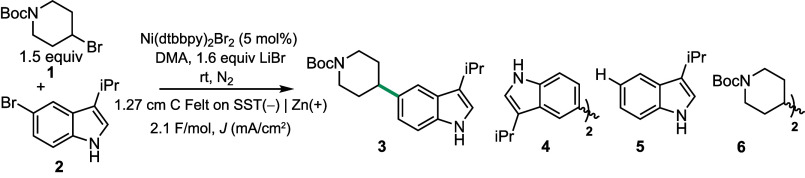
Screening Concentration of **2** to Eliminate Precipitation of **6** in 4 cm^2^ Reactor^a^

	[**2**]	Mass **2**	*J*	Reaction Time (h)	Charge Passed	**2** ^b^	**3** ^b^	**4** ^b^	**5** ^b^	**6**
Entry	(M)	(g)	(mA cm^–2^)	Actual (Intended)	(F mol^–1^)	(%)	(%)	(%)	(%)	Precipitation
1	0.5	23.65	12	94.3	1.7	5	77	6	5	Yes (72 h)
				(117.5)						
2	0.4^c^	18.92	12	94^d^	2.1	2	86	9	6	Yes (67 h)
				(94)						
3	0.3	23.65	12	96	1.7	1	82	5	7	No
				(117.5)						
**4**	**0.3**	**23.65**	**10**	**140.7** ^e^	2.1	**2**	**87**	**6**	**8**	**No**
				**(140.7)**						

a 1.27 cm in reaction scheme refers to thickness of felt.

b Yields reported from ^1^H NMR spectroscopy with 1,3,5-trimethoxybenzene as an external standard.

c The solution was diluted by 20% at 67 h due to precipitation of **6**.

d 95% conversion of product reached at 67 h (1.5 F mol^–1^).

e 99% conversion of **2** reached at 120 h (1.8 F mol^–1^), but electrolysis continued until 140.7 h (2.1 F mol^–1^).

We observed that the precipitation of the alkyl homodimer **6** was slow, resulting in its appearance only during multiday electrolysis. We compared the results between the standard and small reactors for a 100 mmol scale electrolysis at 0.5 M **2** and current density of 12 mA cm^–2^. The standard cell electrolysis ran for 5.5 h, and immediately upon conclusion of the reaction, no solids were observed. However, solid precipitation of species **6** was observed after the postelectrolysis solution sat for 16 h, consistent with slow nucleation kinetics (Figure S16). We postulated that decreasing overall reaction concentration would aid in solubilizing the dimer **6** and prevent its precipitation during the multiday runs which provide sufficient time for the solid to form. Thus, we conducted dilution studies and determined that a concentration of 0.3 M **2** was sufficient to prevent solid precipitation, even after extended reaction times (Table 3).

Diluting the concentration of **2** to 0.3 M enabled a stable multiday electrolysis with comparable yields of **3** to the optimized electrolysis in the 86 cm^2^ standard parallel plate reactor (87% vs 83%, respectively). The 4 cm^2^ small parallel plate reactor demonstrated less sensitivity to current density, as running at either 10 or 12 mA cm^–2^ resulted in >80% product **3** (entries 3–4, Table 3). This is likely because the same flow rate of 200 mL min^–1^ was used for both the 86 cm^2^ standard and 4 cm^2^ small parallel plate cells, reducing the relative amount of time the catalyst is exposed to the electric field per pass through the reactor in the small cell. However, as shown in Table 2, in the 86 cm^2^ standard reactor, 0.3 M **2** required a current density of 10 mA cm^–2^ to prevent lower conversions and yield. Thus, our reoptimized reaction conditions with 0.3 M **2** and a current density of 10 mA cm^–2^ struck a balance between the need to increase reaction concentration to achieve high productivity and the need to maintain a homogeneous reaction mixture to ensure robustness.

It should be noted that the multiday electrolysis conducted at 0.3 M **2** (entries 3–4, Table 3) reached full conversion of **2** at less than the theoretically required 2.1 F mol^–1^ of charge (Table 3, Figure S19). A control experiment in which the reaction solution was circulated through the reactor in the presence and absence of applied current revealed that a small quantity of product **3** formed when the current was turned off for 24 h, suggesting the presence of a minor nonelectrochemical pathway with slow kinetics (Figure S20). Entry 4 in Table 3 reached full conversion of **2** after passing 1.8 F mol^–1^ of charge. Continuing electrolysis until 2.1 F mol^–1^ of charge did not result in product **3** degradation but caused a significant increase in cell voltage (Figure S19). The mechanistic origins of this phenomenon remain unclear, but for practical implementation, a shorter reaction time is unproblematic. Overall, the results of our multiday studies highlight the importance of conducting long-term electrolysis during scale-up, as reaction behaviors can arise on the multiday scale that are otherwise obfuscated by short reaction times.

### Large Cell Design and Validation

Having identified the maximum current density and concentration of **2** compatible with multiday electrolysis, we moved to scaling the reactor itself (Figure 3). We chose electrode dimensions of 29 × 29 cm, informed by the commercial availability of 30 × 30 cm carbon felt. The main changes from the smaller scale reactor designs were (1) the fluid flow through the reactor and (2) the method of attachment of the carbon felt to the stainless steel current collector. Because we were using 1.27 cm (0.5”) thick carbon felt, the high-density polyethylene (HDPE) frame surrounding the felt was also 1.27 cm (0.5”) thick, providing an opportunity to minimize leak points by having the fluid flow exclusively through this felt frame instead of entering and exiting through ports on the front and back. As shown in Figure 3, we designed fluid inlets and outlets on each corner of the reactor frame and evenly spaced 3.2 mm (1/8”) openings to uniformly distribute fluid across the felt. This design required only two ethylene propylene diene monomer rubber (EPDM) O-rings to keep the reactor sealed. On the 86 cm^2^ standard reactor, compression from the anode on the polytetrafluoroethylene (PTFE) mesh was used to prevent shorting and maintain an electrical connection between the carbon felt and current collector. However, for industrial implementation, a more robust connection would be desirable. Thus, we used plastic caps (white circles, Figure 3c) to hold the carbon felt onto the backplate; the caps went through the PTFE mesh and felt and attached to screws on the stainless steel current collector (Figure S7). Commercially available EPDM O-rings were placed between the screw and plastic cap to prevent leaking (Figure S7), but the screws could also be soldered to the current collector for a permanent seal.

**Figure 3:**
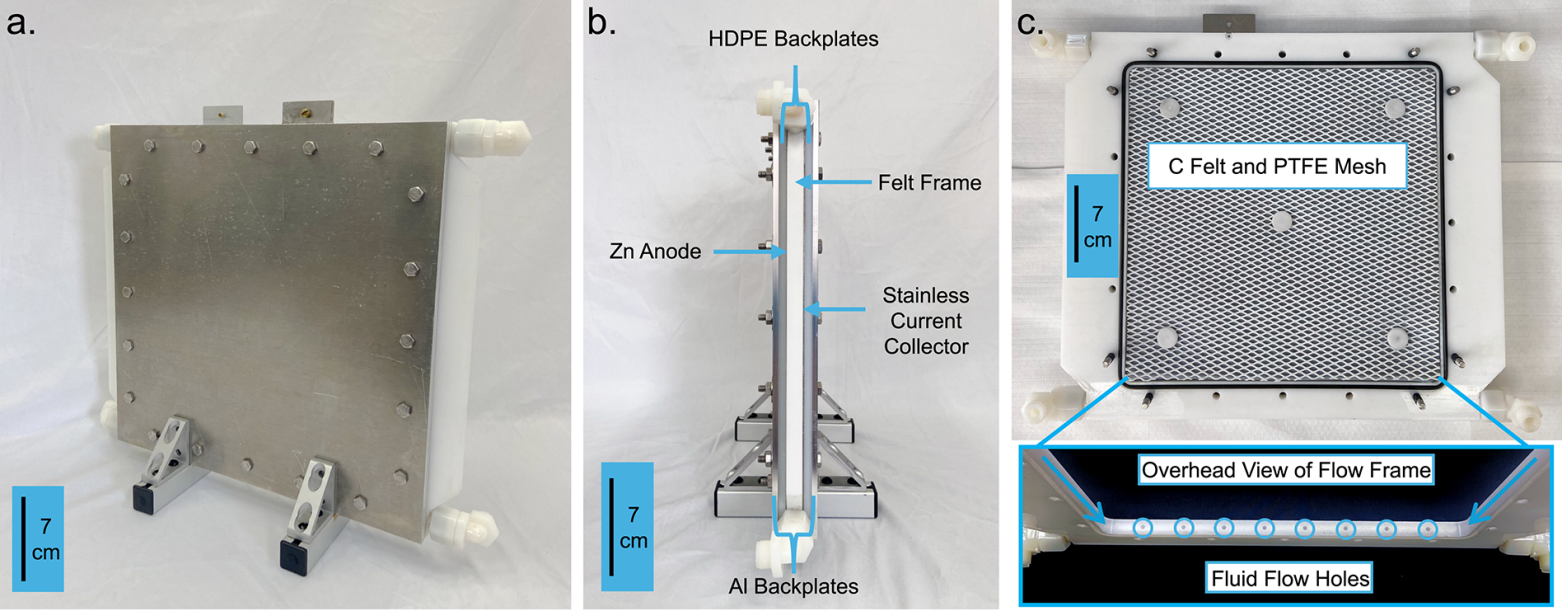
(a) In-house designed and fabricated large reactor on stand. Tabs on top are screw terminal connection points for the electrical leads. (b) Side profile of reactor with the layers of the reactor labeled. (c) Overhead view of felt frame flow plate with carbon felt attached to stainless steel backplate with plastic caps (white circles) and covered in 2 layers of PTFE mesh to prevent shorting against the anode. Inset shows the fluid flow openings on the bottom and top of the felt frame. Fluid inlet and outlet connections are on each corner of the frame. Electrode dimensions are 29 × 29 cm.

To validate the performance of our large reactor, we conducted a 400 mmol scale electrolysis with the same reaction conditions as the optimized multiday electrolysis of 0.3 M **2** and a current density of 10 mA cm^–2^, corresponding to an applied current of 8.41 A (Figure 4a). The higher applied current and larger reactor introduced the possibility of temperature increases and pressure drop across the cell. Thus, we introduced temperature and pressure sensors at the reactor inlet and outlet, as well as an ice water bath around the recirculation vessel to provide cooling (Figure 4b,c). A flow rate of 1.8 L min^–1^ was employed to maintain consistency with the nominal space time of ∼ 0.5 min in the 86 cm^2^ standard reactor. As shown in Figure 4d, the large reactor performed comparably to the 86 cm^2^ standard reactor, with 94% conversion of starting material **2** and 83% assay yield of product **3**. The temperature change and pressure drop across the cell were negligible (Figures S21, S22), and the cell voltage (Figure 4d) moderately increased during the electrolysis as expected. Overall, these results confirmed that the reaction successfully translated to the large reactor without a change in performance.

**Figure 4:**
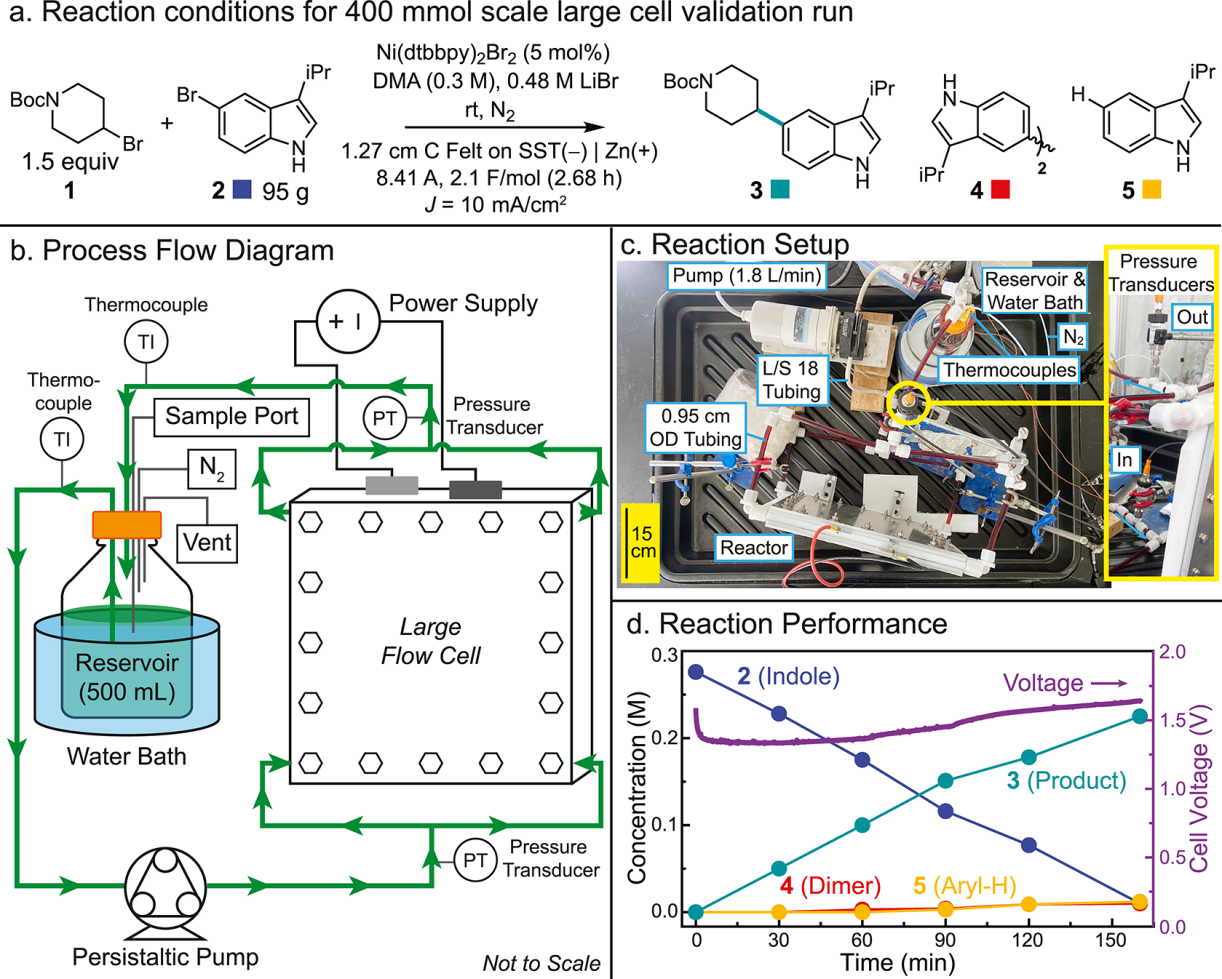
(a) Optimized reaction conditions for 400 mmol scale validation run to verify performance of large cell. Nominal concentration of **2** (indole) relative to DMA at 0.3 M displayed. (b) Process flow diagram of 400 mmol scale validation run. Entire setup was inside of a ventilated enclosure. Green lines indicate fluid flow path. (c) Overhead image of reaction setup inside ventilated enclosure. Inset shows inlet and outlet pressure transducers. (d) Reaction performance over time, left *y* axis displays concentration and right *y* axis displays cell voltage. Reaction performance vs charge passed provided in Figure 6 (blue triangles).

### Kilogram Scale Synthesis

With the performance of the large reactor validated, the final step of our scale-up was completing a reaction using 2.92 mol of **2**, which corresponds to a theoretical yield of 1 kg of **3**. Given the increased scale of the reaction, we first assessed the safety of the reaction mixture. Despite the presence of an electrified reactor, Ni-catalyzed XEC does not feature some of the risks associated with other organic electrosyntheses. For example, this reaction does not involve hydrogen evolution at the cathode, removing a flammability hazard and precluding the need to dilute the generated H_2_ with a constant flow of an inert gas. In addition, this chemistry employs DMA, a solvent with a low vapor pressure and high flash point, making it relatively safe for use at room temperature.^46^ The catalyst is oxygen-sensitive, requiring the system to be under inert gas, further reducing ignition hazards in the event of spark generation. Despite the ampere-level currents employed, the cell voltage during the kilogram run remained below 2 V, allowing the use of a 20 V/20 A power supply and minimizing the risk of electric shock. The low cell voltage also results in a low power draw, eliminating the need to actively cool the cell.

The primary change from the 400 mmol validation electrolysis was replacing the recirculation vessel from a bottle to a jacketed 20 L vessel with an overhead impeller. Furthermore, because the recirculation vessel did not fit inside our ventilated enclosure, the inlet and outlet lines to and from the reactor were fed into the ventilated enclosure and the recirculation vessel had a vent line that was directed to the building exhaust system. Additionally, shut-off valves were added to the inlet and outlet lines both inside and outside of the ventilated enclosure in case of an emergency (Figure 5).

**Figure 5:**
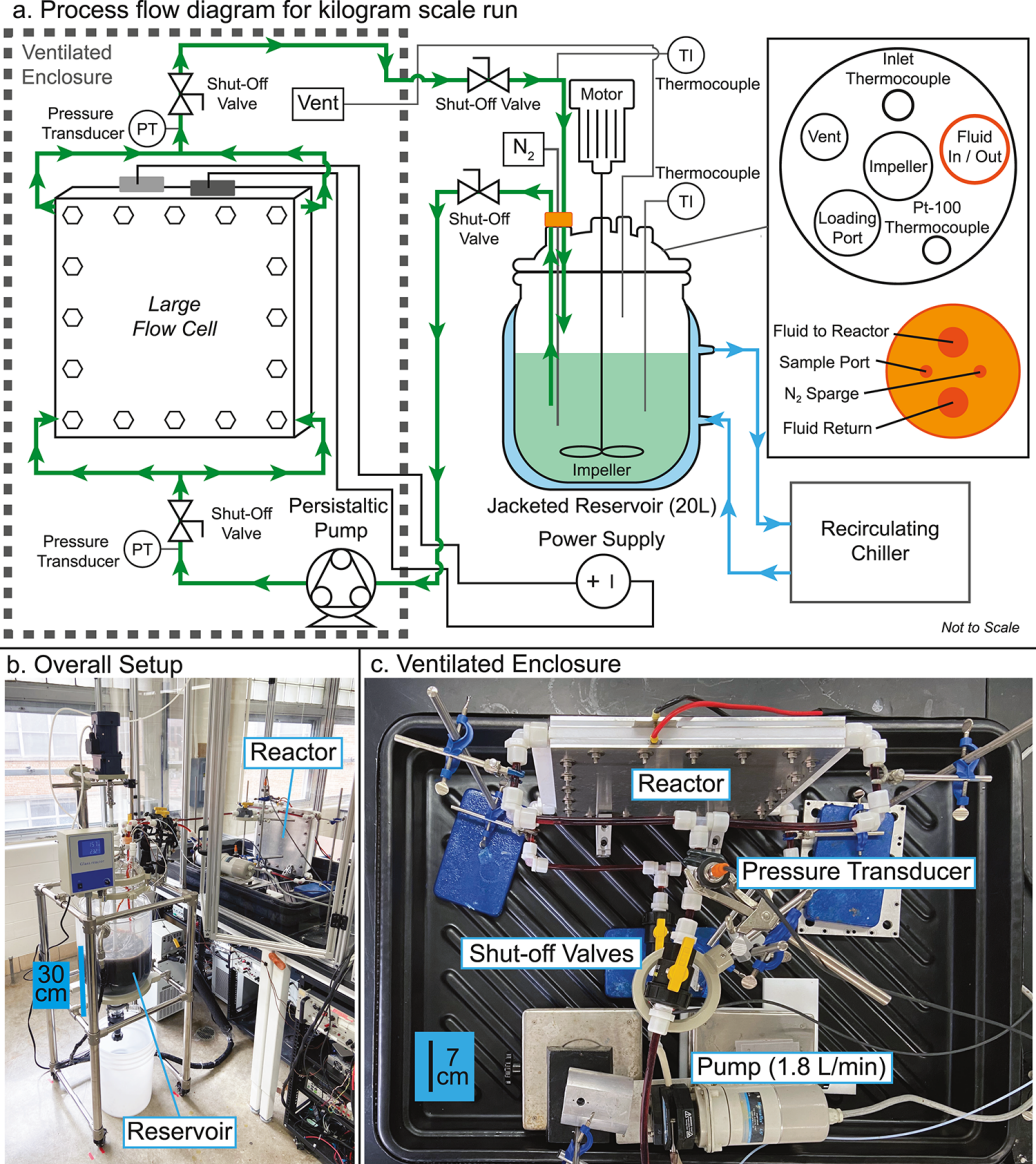
(a) Representative process flow diagram for kilogram scale run. Green lines indicate fluid flow path. (b) Image of overall reaction setup for kilogram scale run. The reactor is inside of a ventilated enclosure. (c) Overhead view of ventilated enclosure with reactor, pump, pressure transducers, and shut-off valves.

As shown in Figure 6, the kilogram scale electrolysis ran for 19.55 h with an applied current of 8.41 A and featured full conversion of **2** and 82% assay yield of product **3**. The cell voltage profile was consistent with prior runs. The temporary spikes seen at approximately 6 and 15 h arose from unintentional bubble introduction into the reactor inlet line from the N_2_ sparge line (Figure 6b). As shown in Figure 6c, upon conclusion of electrolysis and flushing the reactor with DMA, the PTFE mesh and cathode showed no evidence of fouling, and visually, the Zn anode showed uniform oxidation over the entire wetted area. There was also no evidence of precipitation of homodimer species **6**. Similar to the 400 mmol validation electrolysis, the pressure drop and temperature increase across the cell were negligible (Figures S23, S24). The combination of visually even oxidation of the Zn anode as well as negligible pressure drop suggests that the flow holes of the larger reactor enable sufficiently uniform flow distribution across the cell for consistent reaction performance. A cooling jacket around the recirculation vessel was used with a recirculating chiller, maintaining the solution temperature at ∼ 20 °C when the jacket temperature was kept between 18.5 and 20 °C. These results highlight the ability to scale the reaction seamlessly across 2 orders of magnitude with no significant difference in product yield (Table 4, Figure 7). After an aqueous workup and seeded crystallization from isopropanol/water, the desired product **3** could be obtained in 62% isolated yield in high purity with respect to the impurities **4** and **5** (Figure 6a). Furthermore, the workup demonstrated excellent control over residual metals, as X-ray fluorescence (XRF) indicated Zn or Ni present at <20 ppm levels in the isolated **3**.

**Figure 6:**
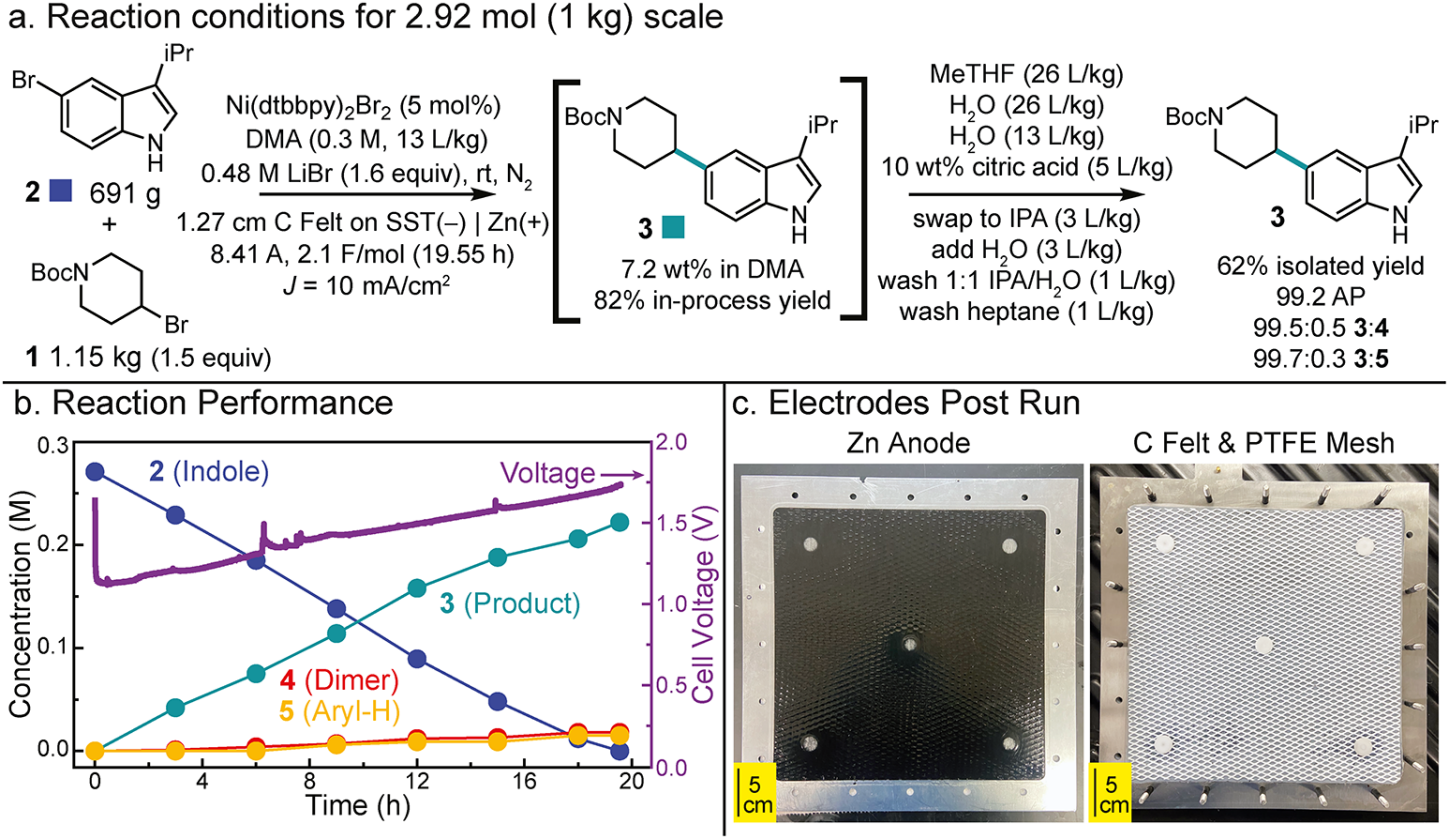
(a) Reaction conditions and isolation procedure for 1 kg scale run. Nominal **2** (indole) concentration of 0.3 M displayed. AP = assay purity, 1.27 cm C felt referring to felt thickness. (b) Reaction performance over time, left-hand *y* axis displays concentration and right-hand *y* axis displays cell voltage. Reaction performance vs charge passed provided in Figure 6 (blue squares). (c) Appearance of Zn anode and carbon felt cathode with both PTFE mesh layers attached upon conclusion of reaction. Reactor was emptied and rinsed with 1.6 L of DMA flowing at 1 L min^–1^ prior to photo being taken.

**Figure 7:**
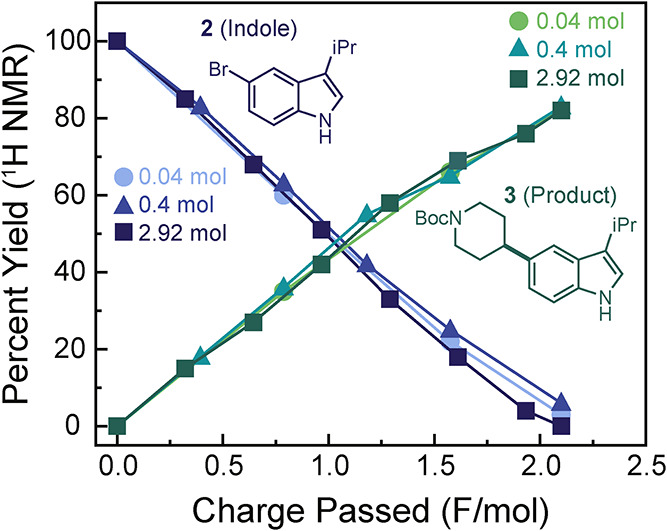
Comparison of reaction performance across three different scales, 0.04 mol, 0.4 mol, and 2.92 mol.

**Table 4:** Comparison of Reaction Performance across Scales and Electrode Areas

Entry	Reaction Scale (mol)	Electrode Area (cm^2^)	**2 (%)** ^a^	**3 (%)** ^a^	**4 (%)** ^a^	**5 (%)** ^a^
1	0.04	86	3	82	8	3
2	0.1	4	2	87	6	8
3	0.4	841	6	83	7	4
4	2.92	841	0	82	5	13

a Yields reported from ^1^H NMR based on mol % with 1,3,5-trimethoxybenzene as an external standard.

Based on the successful kilogram scale-up, we completed calculations for conducting this reaction at a nominal 100 kg scale in an industrial production campaign. If the campaign is completed in 2 × 50 kg batches, 8 cells (a total reactor footprint <0.1 m^3^, Figure S26) running at 10 mA cm^–2^ (67.3 A) would result in a batch length of 5.1 days — the same time frame as our successful multiday electrolysis. Estimating an average cell voltage of 1.5 V per cell (average cell voltage during kg scale run was 1.43 V), the total power requirement for the reactors would be ∼ 100 W (Table S3), and for the entire production campaign, the estimated electrical energy required by the reactors is a mere 25 kWh (Table S4). The Zn requirements for this scale of production are reasonable; 20.05 kg of Zn would be required, and the industrial price of Zn is below 5 USD per kg.^47^ The expected change in thickness for each Zn anode is less than 2.5 mm per 50 kg batch (see Supporting Information for details). This small change in anode thickness significantly reduces the risk of leaking from anode dissolution, as Zn anodes up to 1” thick are readily commercially available. Our multiday electrolysis demonstrated that such a change in thickness does not significantly impact the flow profile or magnitude of the cell voltage. Furthermore, as shown in Figure S25, the voltage profiles and final voltage for the 120 and 19.55 h runs are similar, despite the expected changes in anode thickness being nearly an order of magnitude different (2.4 mm vs 0.33 mm, respectively). The modest electrical and Zn requirements, coupled with the proven scalability of this reaction, lend credence to implementation of this process on yet larger scales.

## Conclusion

In this work, we successfully completed the kilogram-scale synthesis of an API intermediate using electrochemical Ni-catalyzed cross electrophile coupling. A 5-day 100 mmol electrolysis ran with no change in overall reaction performance, reproducing results obtained at significantly smaller scales. We also demonstrate kilogram-per-day productivity on a single electrode stack, with an overall reactor footprint less than 0.01 m^3^. Key to achieving this scale-up was an understanding of the interplay between substrate concentration, current density, and reactor design. Additionally, validating reaction parameters on a multiday scale prior to further scale-up revealed an unforeseen solid precipitation which could be mitigated by substrate dilution. Maximizing productivity while maintaining multiday stability at intermediate scales of ∼100 mmol batches allowed for subsequent seamless scale up to the 2.92 mol scale.

Informed by these results, we can ask if Ni-catalyzed XEC is an electrosynthetic method suited to large scale implementation that meets the criteria of an ideal electrochemical process — safety, environmental friendliness, high productivity, cost efficiency, and process robustness.^1^ Addressing safety, the low voltages and lack of H_2_ generation at the cathode significantly reduce electrical hazards and flammability concerns. From a sustainability and cost standpoint, electrochemical Ni-catalyzed XEC provides an opportunity to replace Pd-catalyzed routes and could be driven with renewable electricity. This potential financial benefit could help offset the investment required to develop electrochemical setups and know-how within pharmaceutical process groups and contract manufacturers. High productivity is achieved thanks to the high current density attained in a compact reactor. Cost efficiency is attained due to the high yield of the process compared to existing methods as well as the low cost of the reactor, which features inexpensive and commercially available electrode materials that require no customized pretreatments or coatings. Furthermore, our simplified reactor design was machined in-house via traditional computer numerical control machining — a widely available technology. The ease of fabrication lowers the barrier to implementing electrochemical reactors on a wider scale. Finally, the robustness of the process was impressive. Performance did not change across 2 orders of magnitude in both scale and time, and the product was not adversely impacted by overelectrolysis. The isolated product also featured very low levels of residual metals despite the use of a Ni catalyst and sacrificial Zn anode. Taken together, these results are another step in demonstrating the feasibility of using electrosynthesis in the pharmaceutical industry.

## Supplementary Material



## Notes

Additional experimental details, data, technical drawings, and compound spectra (PDF)

## Notes

Safety Statement: The use of a sacrificial Zn anode creates a layer of high surface area Zn, which theoretically could render the spent anode pyrophoric. In practice, we did not observe any pyrophoric behavior of the anodes postreaction, and they were stable in ambient air both with and without rinsing with solvent for cleaning.

## Abbreviations

XEC: cross electrophile coupling
TLR: toll-like receptor
API: active pharmaceutical ingredient
IY: isolated yield
AY: assay yield
AP: assay purity
DMA: N,N-dimethylacetamide
DIPEA: diisopropylethylamine
SST: stainless steel
HDPE: high density polyethylene
EPDM: ethylene propylene diene monomer
PTFE: polytetrafluoroethylene
XRF: X-ray fluorescence
NMR: nuclear magnetic resonance
