# Supplementary material for: Development of Kilogram-Scale Electrochemical Ni-Catalyzed Cross Electrophile Coupling in Flow
Source: Org Process Res Dev. 2026 Jun 16;30(7):1926–36. doi: 10.1021/acs.oprd.6c00110 (PMC13386638; doi:10.1021/acs.oprd.6c00110)
Supplement: Supplementary file 1 [file op6c00110_si_001.pdf]

Supporting Information

Development of Kilogram-Scale Electrochemical Ni-Catalyzed Cross Electrophile Coupling in Flow

*Megan Kelly,[1] Luana Cardinale,[2] Karrin V. Sackett,[1] Suqi Zhang,[1] Gregory L. Beutner,[3] Benjamin Cohen,[3] Shannon S. Stahl,[2] Marcel Schreier[1,2]**

[1] Department of Chemical and Biological Engineering, University of Wisconsin-Madison, Madison WI, 53706, United States
[2] Department of Chemistry, University of Wisconsin-Madison, Madison, WI, 53706, United States
[3] Chemical Process Development, Bristol Myers Squibb, 1 Squibb Drive, New Brunswick, New Jersey 08903, United States

Contents

General Experimental Procedure S2
Setup Photos S5
General Procedure for Initial Optimization Study S16
General Procedure for Production Rate Optimization Runs on Standard Flow Cell S17
Flow Rate Selection S19
General Procedure for Multiday Electrolysis Using Small Cell (4 cm^2) S21
Solid Formation During Multiday Electrolysis S23
Successful Multiday Electrolysis Performance S27
Multiday Electrolysis Finishing Before 2.1 F mol^{-1} S28
General Procedure for 400 mmol Large Cell Validation S30
Cell Pressure and Temperature Data for 400 mmol Validation Run S31
General Procedure for 1 kg Scale Run S32
Cell Pressure and Temperature Data for 1 kg Scale S33
Voltage Trace Comparison S34
Estimating Expected Change in Zn Anode Thickness S35
Power and Energy Requirement Calculations for 100 kg Production Campaign S36
Product Isolation Procedure S37
Spectra of Compounds S38
Technical Specifications and Drawings S41

General Experimental Procedure

Electrode Sourcing

Resin Filled Graphite Current Collector: Fuel Cell Store

C Felt – Fisher Scientific

Zn: Online metals and McMaster Carr

Stainless Steel Current Collector: McMaster Carr

PTFE Mesh: Industrial Netting ET8120 (fine), ET8900 (coarse)

Materials Sourcing

**Compound**	**Source**	**Purity**
N,N Dimethylacetamide	Oakwood Chemical	99%
Ni(II)Br_2·3H2O	ChemCruz	99.9%
4,4'-di-tert-butyl-2,2'-bipyridine	Ambeed*<br>ChemScene<br>CombiBlocks	98%<br>98%<br>98%
LiBr Anhydrous	Sigma Aldrich	≥99%
4-bromo-1-boc-piperidine	Ambeed	98%
5-bromo-3-isopropyl-1H-indole	See synthesis beolow	
Me-THF	Sigma Aldrich	≥99.5% w/ 140-500 ppm BHT
Diethyl Ether	Sigma Aldrich	≥99% w/ BHT
THF	Supelco	≥99.9%
N-Heptane	Fisher Scientific	99%
IPA	VWR Chemical	≥99%
HCl	Sigma Aldrich	ACS Reagent 37%
Ethanol	Decon Laboratories	≥99%
1,3,5-trimethoxybenzene	Thermo Scientific	99%
$CDCl_3$	Sigma Aldrich	≥99.8%
Acetone	VWR Chemical	≥99.5%

*Note: Lot AS7 of ligand from Ambeed contained an unknown impurity that adversely affected reaction performance.

5-bromo-3-isopropyl-1H-indole Synthesis

Indole synthesis was performed at Bristol Myers Squibb using an existing literature procedure.[31]

In a jacketed 5 L reactor equipped with an overhead stirrer and reflux condenser under N_2, 440 g of 5-bromoindole (2.24 mol, 1.0 equiv) was added to 2.3 L of toluene (5 mL/g) and 235 mL of acetone (3.316 mol, 1.4 equiv). The reaction was cooled to 0 °C, and 245 mL of trifluoroacetic acid (3.316 mmol, 1.4 equiv) was slowly added over ~30 min, maintaining an internal temperature below 10 °C. The resulting brown solution was held at 0 °C for 30 min and then charged with 758

mL of triethylsilane (4.7 mol, 2.1 equiv) in a single portion at 0 °C. The resulting brown solution was heated to 45 °C for 16 h and cooled to 23 °C; then a solution of 945 g of potassium carbonate (6.72 mol, 3.0 equiv) in 1.35 L of water was added. The solution was aged for 1 h; then, the lower aqueous layer was removed, and 200 mL of 5 wt % brine was added. Again, the lower aqueous layer was removed. The organic layer was dried over 75 g $MgSO_4$. Then the solution was filtered through a thin pad of Celite, charged with 7.5 g of activated carbon, aged with stirring for 1 h, filtered through a thin pad of Celite, and concentrated to a light brown oil. Then, 1.4 L of heptane was added, and the solution was heated to 50 °C. A slow cool down to 35 °C commenced over 1 h, and then the solution was charged with 0.375 g of authentic **2**. The solution was held at 30 °C for 1 h, and a very thick, heavy slurry formed, which was cooled to 10 °C and held for at least 3 h. The residue was filtered, washed with 428 mL of heptane, and dried under a N_2/vacuum sweep to yield 424 g of **2a** white crystalline solids (79% yield). Mp: 68 °C (DSC). TLC (7:3 hexanes/EtOAc, R_f = 0.58 (UV 254 nm)). ^{1}H NMR ($CDCl_3$, 500 MHz): δ 7.88 (bs, 1H), 7.79 (d, J = 1.8 Hz, 1H), 7.27 (dd, J = 8.6, 1.8 Hz, 1H), 7.21 (d, J = 8.6 Hz, 1H), 6.96 (br d, J = 2.3 Hz, 1H), 3.16 (sept, J = 6.8 Hz, 1H), 1.36 (d, J = 6.8 Hz, 6H). $^{13}C\{^1H\}$ NMR ($CDCl_3$, 125 MHz): δ 135.1, 128.5, 124.6, 123.8, 121.9, 120.5, 112.5, 112.3, 25.3, 23.2. IR (SS ATR): 1469, 1456, 1418, 1228, 1102, 1076 cm^{-1}. HRMS (ESI): m/z $[M + H]^+$ calcd for $C_{11}H_{12}BrN$, 238.0226; found, 238.0226 (0.04 ppm error).

Electrode Cleaning

Carbon Felt:

For runs on the 4 cm^2 and 841 cm^2 electrodes, new felt was used each time. For the 86 cm^2 run, the felt was cleaned as follows: Reactor emptied and flushed with ethanol. Felt rinsed with acetone / lightly compressed until acetone ran clear. Felt soaked in acetone with gentle stirring for at least 6 h. Felt rinsed thoroughly with DI water. Felt soaked in 1M HCl for at least 6 h. Felt rinsed thoroughly with DI water – if desired, pH of residual water running off felt can be checked to ensure it is neutral. Felts dried in an oven at 140 °C least overnight. To cut felt to necessary shapes, scissors were used.

Zn Anodes:

Zn was rinsed with acetone to remove organic residue and allowed to air dry. 1M HCl was squirted onto oxidized areas to remove initial layer of black Zn then rinsed with water. Polished with sandpaper (100 grit). On 841 cm^2 electrode, mechanical sander was used (Dewalt ¼ Sheet Palm Grip Sander). On Zn electrodes for the small cell on multiday runs, electrodes were taken to machine shop to be polished to re-establish a flat surface.

Catalyst Synthesis

To a clean and dry glass bottle equipped with a PTFE stir bar, 1 equivalent of $NBr_2·3H_2O$ and 2 equivalents of dtbbpy (4,4'-di-tert-butyl-2,2'-bipyridine) were charged. Stirring was initiated, and 6 volumes of EtOH (mL EtOH : mmol $NiBr_2·3H_2O$) were added. The reaction solution was stirred overnight until a deep blue solution formed. The solution was then concentrated to ~1/10 of the original volume, and a solution of 1:4 THF / Et_2O was added, with the total volume of the solution

being roughly 5/6 of the original volume of EtOH used (see volumes used below). If the precipitation was insufficient and the solution did not turn opaque, more Et_2O can be added. The solution was stirred for ~10 min and then filtered and washed with Et_2O. The obtained solid was left to dry under air overnight. The product was obtained as a pale green / blue solid.

The following scales of the synthesis were run:

15 gram: 20 mmol (5.46 g) $NiBr_2{\cdot}3H_2O$, 40 mmol (10.8 g) dtbbpy in 120 mL EtOH

Synthesis performed in 250 mL glass bottle, 100 mL 1:4 THF / Et_2O used for precipitation

30 gram: 40 mmol (10.92 g) $NiBr_2{\cdot}3H_2O$, 80 mmol (21.6 g) dtbbpy in 240 mL EtOH

Synthesis performed in 500 ml glass bottle, 200 mL 1:4 THF/Et_2O used for precipitation

Power Supplies

Standard 86 cm^2 cell electrolysis was powered by either a Kepco BOP 20/20 power supply or a Keithley 2230-30-1 triple channel DC power supply. Large cell electrolysis was powered with a Kepco BOP 20/20 power supply. Multiday electrolyses were powered by either a Kepco BOP 20/20 power supply, Keithley 2230-30-1 triple channel DC power supply, Gamry Interface1010E potentiostat, or a Gamry Reference 600+ potentiostat.

^{1}H NMR Procedure

Assay yields were determined via ^{1}H NMR conducted on either a 400, 500 or 600 MHz Bruker NMR spectrometer. 1,3,5-trimethoxybenzene was added to the sample post-electrolysis as an external standard.

X-Ray Fluorescence

X-ray fluorescence spectroscopy (XRF) for Ni and Zn was performed on a Malvern Panalytical Epsilon 1 spectrometer.

Setup Photos

Small Reactor

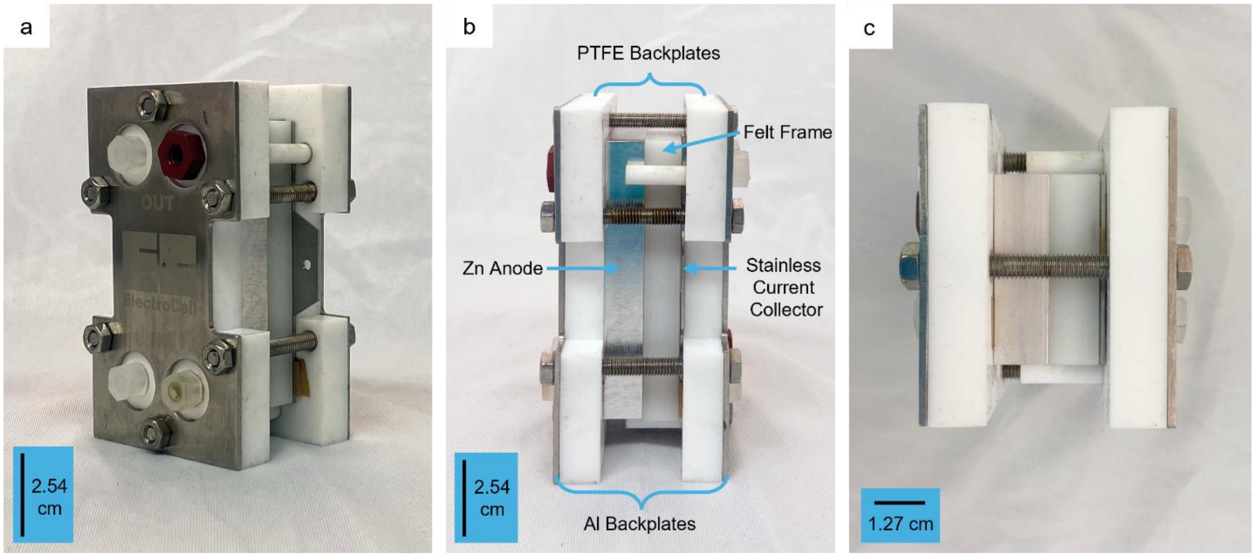

Figure S1. Small reactor used for multiday electrolysis, geometric electrode size was 4 cm^2 **(a)** Front view of assembled reactor, red port on upper right hand is for fluid flow **(b)** Side view of small reactor with main components labeled **(c)** Overhead view of assembled reactor.

Large Reactor

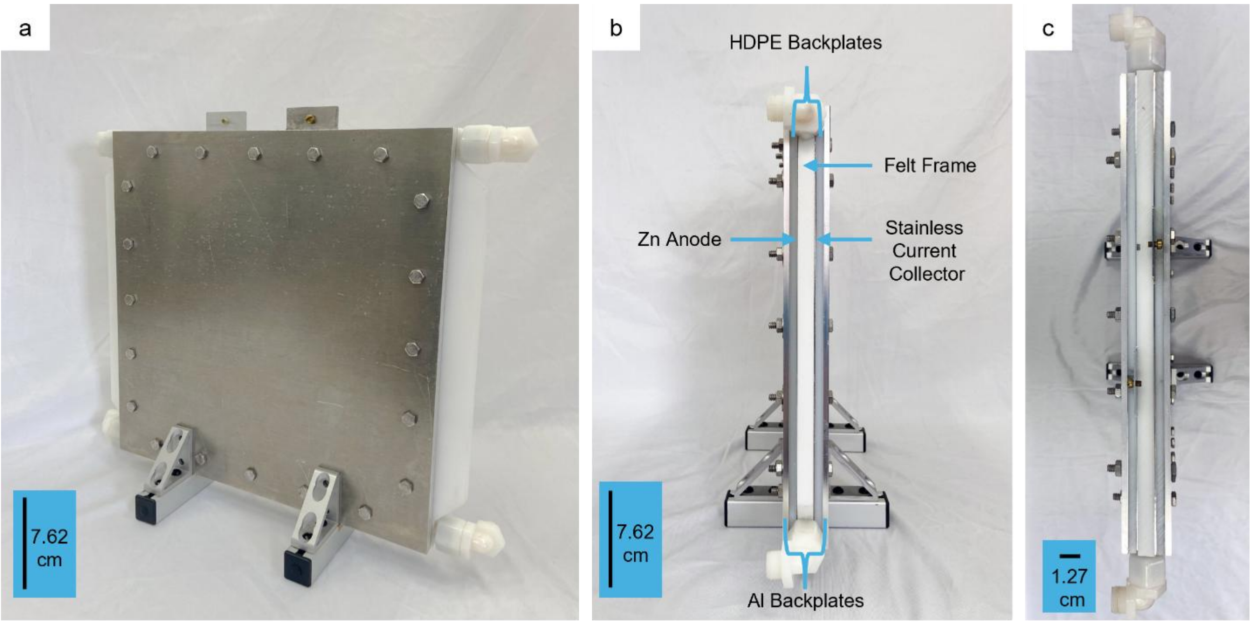

Figure S2. Large reactor used for 400 mmol validation run and kilogram scale electrolysis **(a)** Front view of assembled reactor **(b)** Side view of large reactor with main components labeled **(c)** Overhead view of assembled reactor.

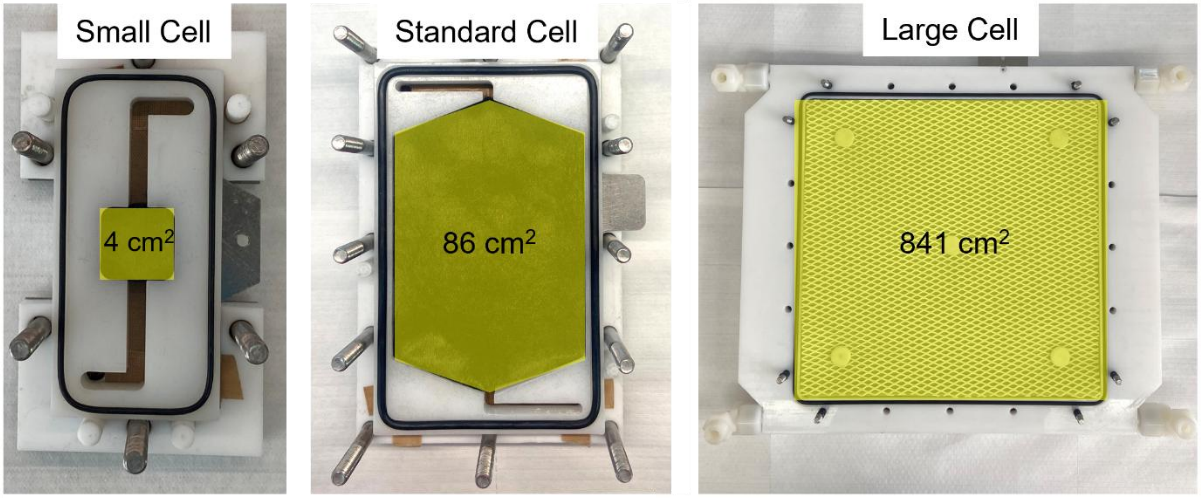

Figure S3. Nominal geometric area of each reactor's electrodes used for the current density metric, shown with the yellow highlighted shape. Note that the slight curvature of the corners of the small and large cell were not used in the calculation, a perfect square geometry was used for simplicity.

Standard Reactor Assembly

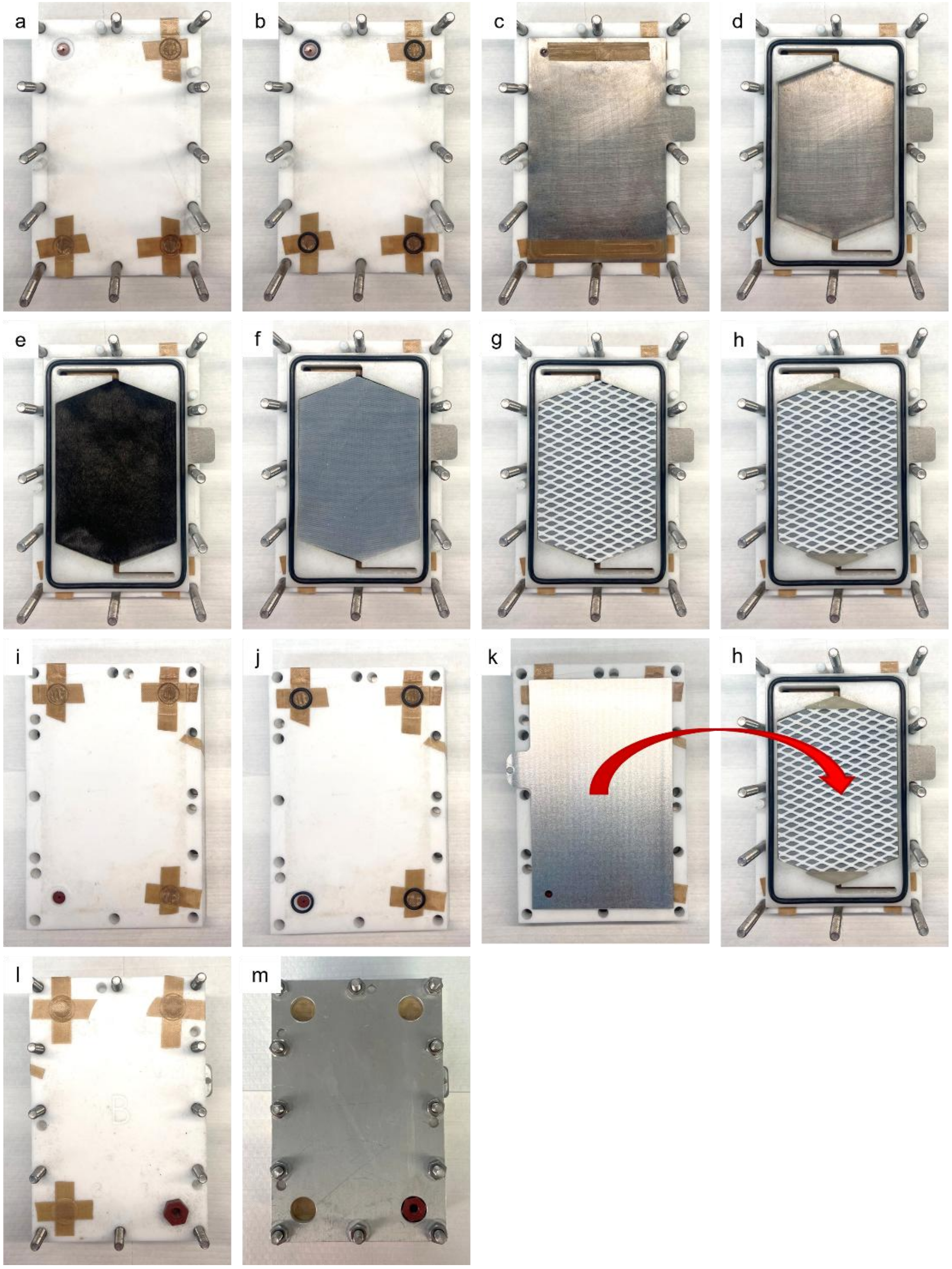

Figure S4. Step by step images of standard reactor assembly, see below for detailed instructions.

Standard Reactor Assembly Steps

1. Feed bolts through ¼" aluminum backplate and PTFE backplate and invert (**Fig S5a**).
2. Place EPDM o-rings onto PTFE backplate grooves (**Fig S5b**)
3. Place stainless steel current collector on top of PTFE backplate and o-rings (**Fig S5c**).
4. Use PTFE tape to cover stainless steel exposed by flow channel (**Fig S5d**).
5. Place desired HDPE C felt frame (0.25" or 0.5" depending on felt thickness. 0.5" shown in image) with o-rings in place on top of current collector (**Fig S5e**).
6. Place 86 cm^2 C felt electrode cut from 0.5" thick sheet in felt frame (**Fix S5f**).
7. Place fine PTFE mesh over felt electrode (**Fig S5g**).
8. Place coarse PTFE mesh over fine mesh on the electrode
9. Place triangular electrode holders on top of PTFE mesh (**Fig S5h**).
10. Take top PTFE frame and place next to cell assembly (not shown) (**Fig S5i**).
11. Place EPDM o-rings into grooves on top PTFE frame (**Fig S5j**).
12. Place Zn anode on top of the top PTFE backplate and o-rings (**Fig S5k**).
13. Take anode assembly in **Fig S5k**, carefully holding anode to not displace any o-rings, invert and place on top of cell assembly from **Fig S5h** (**Fig S5l**). Ensure HDPE felt frame o-rings did not come out of grooves during this step, if so, remove anode assembly, replace o-rings and try again.
14. Place aluminum top plate on top of assembled cell, add nuts to the bolts and tighten in a star pattern (**Fig S5m**).

Note: C felt available from Fisher comes in 0.25" and 0.5" nominal thicknesses. The 0.5" felt is closer to 3/8" thick as delivered. A 13/32" HDPE felt frame can be used in lieu of a 0.5" felt frame, in this case, the electrode fixers in step 9 are not needed, as the PTFE mesh provides sufficient compression to keep C felt pressed against backplate.

Small Cell Assembly

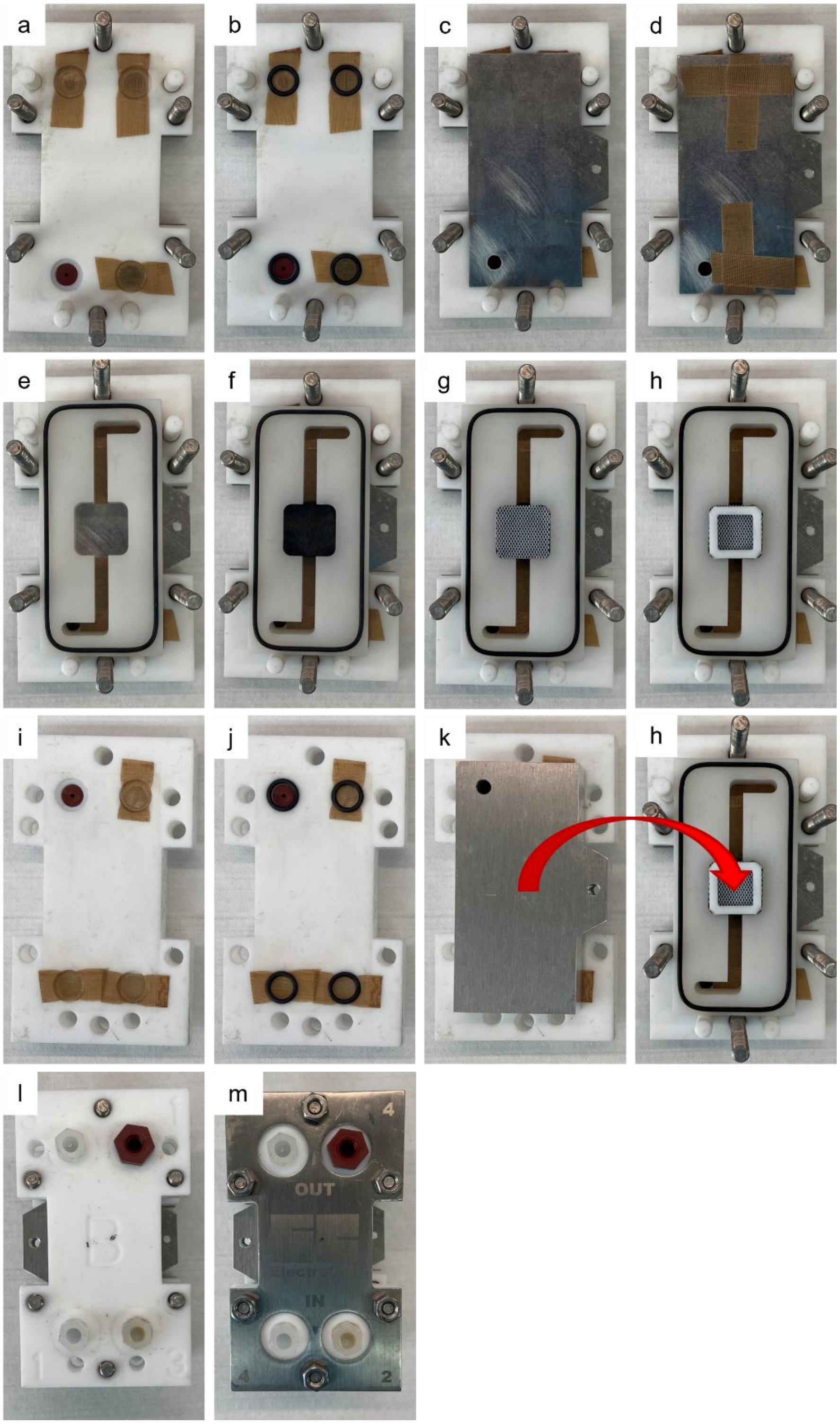

Figure S5. Step by step images of small reactor assembly, see below for detailed instructions.

Small Reactor Assembly Steps

1. Feed bolts through aluminum backplate and PTFE plate and invert (**Fig S6a**).
2. Place EPDM o-rings onto PTFE backplate grooves (**Fig S6b**)
3. Place stainless steel current collector on top of PTFE backplate and o-rings (**Fig S6c**).
4. Use PTFE tape to cover stainless steel exposed by flow channel (**Fig S6d**).
5. Place 0.5" HDPE C felt frame with o-rings in place on top of current collector (**Fig S6e**).
6. Place 4 cm^2 C felt electrode cut from 0.5" thick sheet in felt frame (**Fix S6f**).
7. Place PTFE mesh over felt electrode (**Fig S6g**).
8. Place PTFE electrode holder on top of PTFE mesh (**Fig S6h**).
9. Take top PTFE frame and place next to cell assembly (not shown) (**Fig S6i**).
10. Place EPDM o-rings into grooves on top PTFE frame (**Fig S6j**).
11. Place 0.5" thick Zn anode on top of the top PTFE backplate and o-rings (**Fig S6k**).
12. Take anode assembly in **Fig S6k**, carefully holding anode to not displace any o-rings, invert and place on top of cell assembly from Fig **S6h** (**Fig S6l**). Ensure HDPE felt frame o-rings did not come out of grooves during this step, if so, remove anode assembly, replace o-rings and try again.
13. Place aluminum top plate on top of assembled cell, add nuts to the bolts and tighten in a star pattern (**Fig S6m**).

Large Reactor Assembly

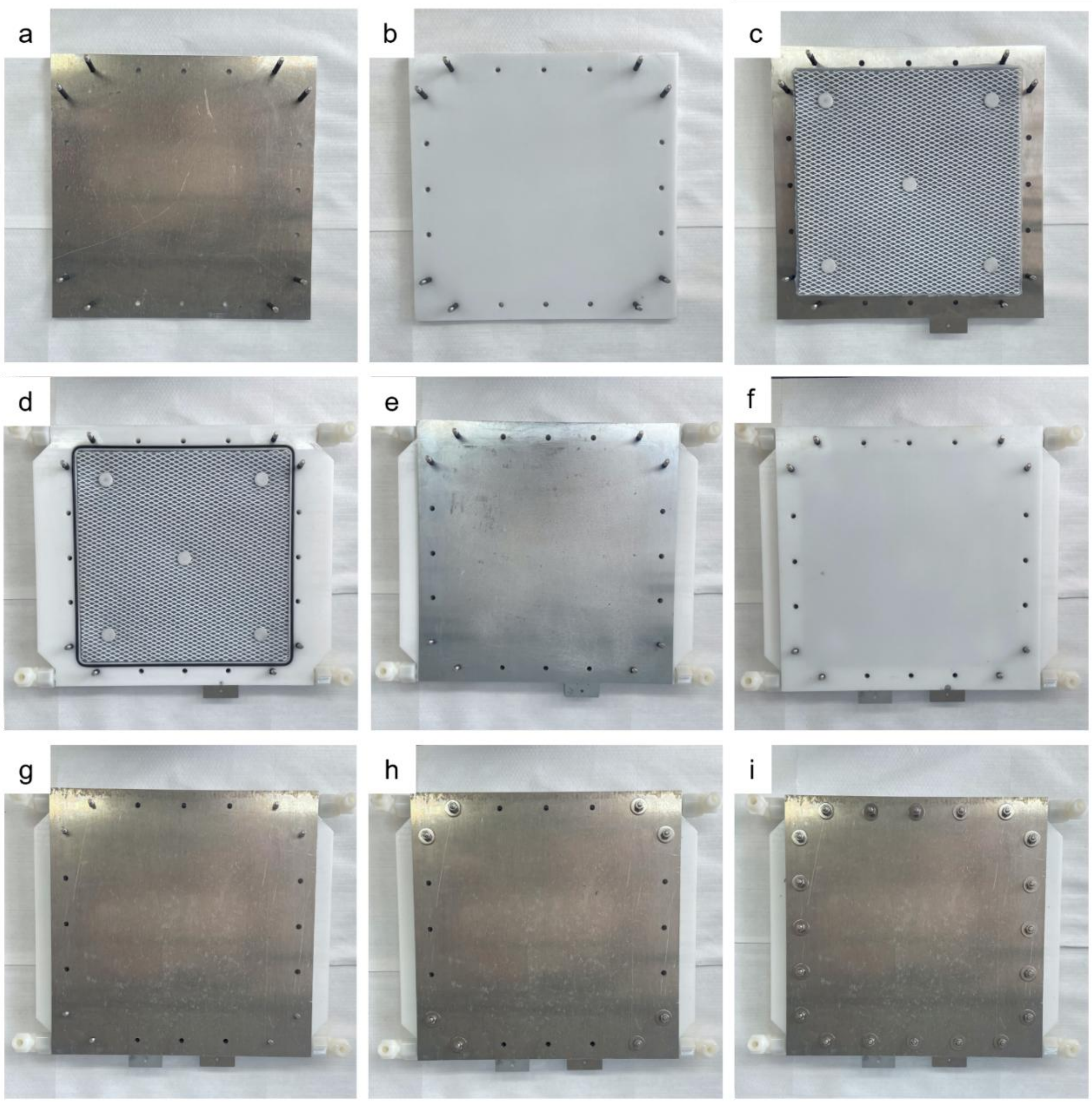

Figure S6. Step by step images of large reactor assembly, see below for detailed instructions.

Large Reactor Assembly Steps

1. Feed 4 insulated bolts through the corner holes of the ¼" Al clamp plate (**Fig S7a**)
2. Place ¼" HDPE backplate on top of Al clamp plate (**Fig S7b**).
3. Place 0.5" C felt with PTFE mesh attached to stainless steel current collector on top of HPDE back plate (**Fig S7c**). See **Figure S8** for details on C felt assembly.
4. Place main flow frame with EPDM o-rings placed in the grooves over C felt assembly (**Fig S7d**).
5. Place Zn anode over flow frame, make sure tab is on the same side as the stainless steel current collector tab for lead connections (**Fig S7e**).
6. Place second ¼" HDPE backplate over Zn anode (**Fig S7f**).
7. Place second ¼" Al clamp plate on top of HPDE backplate (**Fig S7g**).
8. Add washers and nuts to existing bolts in corners of reactor (**Fig S7h**).
9. Add remaining bolts / nuts / washers and tighten in star pattern. Reactor can be placed in stand (not depicted in sequence, see **Fig S3**) for this step.

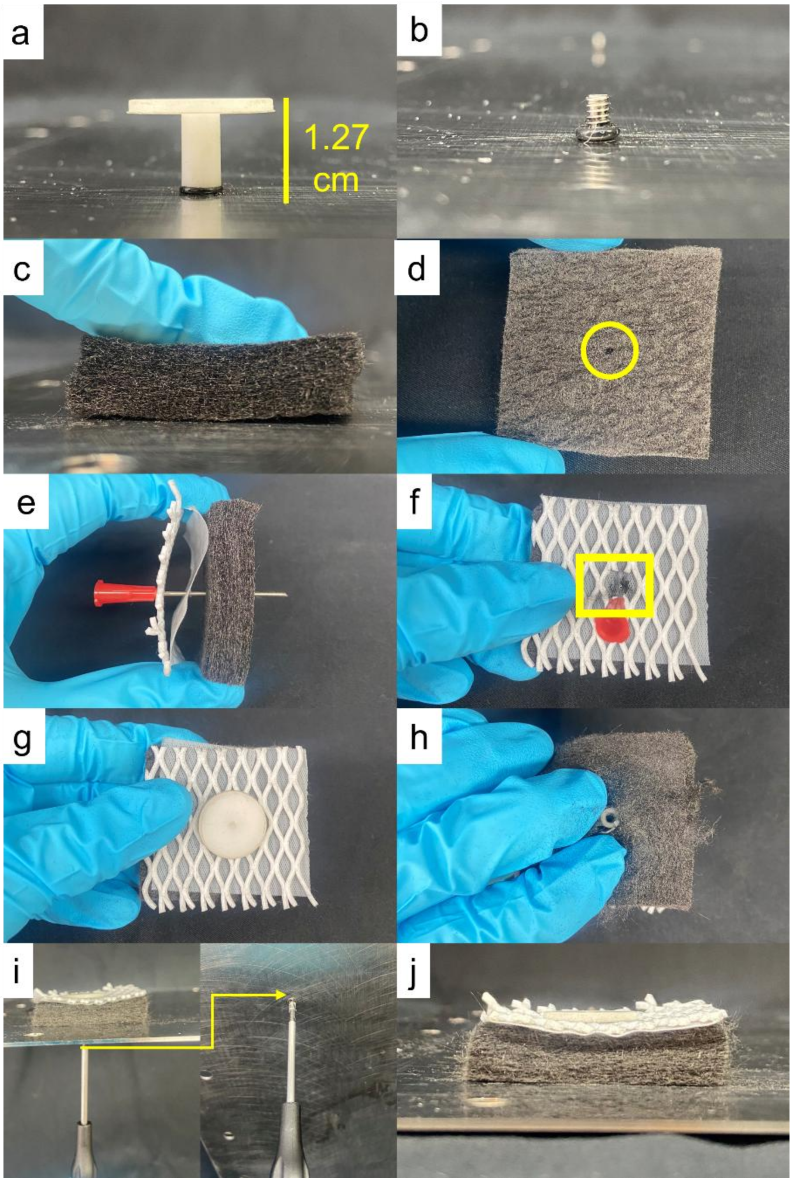

Figure S7. Step by step illustration of how carbon felt and PTFE mesh were secured to the stainless steel current collector in the large cell with the plastic electrode holder screws. A representative piece of felt and mesh being attached to a singular connection screw is shown for clarity. Full size attached felt can be seen in **Fig S3**. **(a)** Plastic electrode holder screw tightened onto o-ring, without C felt or mesh, for visualization. **(b)** Metal screw for connection to screw point and o-ring. **(c-j)** See detailed assembly instructions below. Only blunt needles were used for assembly.

C Felt Assembly Steps

1. Press felt onto back plate to create guide holes by pressing onto the metal screw connections shown in **Fig S8b** (**Figure S8c, S8d**)
2. Using guide hole from step 3, place fine and coarse PTFE mesh (fine on top of felt, then coarse on top of fine mesh), and use a blunt needle fed through the felt guide hole to pierce a hole in the fine mesh (**Fig S8e**)
3. Carefully cut coarse mesh with scissors to allow stem of electrode screw to pass through **(Fig S8f)**
4. Push plastic electrode screw through the felt and meshes (**Fig S8g**) until bottom can be seen through underside of felt (**Fig S8h**)
5. Use screwdriver to fix electrode screw into place by tightening the metal screw on the underside of the stainless steel current collector (**Fig S8i**). May need to hold the plastic screw on top in place (not shown in image).
6. C felt is now affixed (**Fig S8j**). On full size cell, it is recommended to do center hole first, then go from top corners to bottom corners, gently realigning mesh as necessary. It may be helpful to loosely attach each plastic electrode screw first, reposition mesh, then tighten each one fully.

General Procedure for Initial Optimization Study

To an undivided cell (Figure S9) was added a cross-shaped stir bar, $NiBr_2(dtbbpy)_2$ (15.9 mg, 0.02 mmol, 5 mol%) and the two coupling substrates: **1** (0.63 mmol, 166.4 mg, 1.5 equiv) and **2** (0.42 mmol, 100 mg, 1.0 equiv). The cell was then transferred into a N_2-filled glove box where anhydrous LiBr (86 mg, 1 mmol, 0.2 M) was added together with 5 mL of anhydrous DMA (or the chosen solvent). The cell was sealed with a rubber septum containing an RVC electrode (–), a sacrificial Zn anode (+), and a Teflon tube as N_2-inlet. The RVC is cut into a piece of 3 x 1 x 0.5 cm; during electrolysis the electrode is submerged only by 2 cm in height. At this point, the cell was removed from the glovebox and connected through the thin Teflon tube to a N_2-line to allow a positive N_2 pressure during the reaction. The reaction mixture was stirred at 1000 rpm (full dissolution of LiBr can take up to 30 minutes; bulk electrolysis is started only after full dissolution is achieved). The reaction mixture was electrolyzed under an applied constant current of –4 mA ($J = -2$ mA/cm^{-2}), or the specified current, until 2.1 F mol^{-1} were passed (85.1 C). After electrolysis, the cell was opened and exposed to air, and the electrodes were rinsed with EtOAc and 0.2 mL of AcOH. For quantification by 1H-NMR spectroscopy: 0.21 mmol of TMB was added to the crude reaction, and 0.1 mL of this solution was directly diluted in 0.5 mL of $CDCl_3$ and submitted for analysis. Note: For reactions in which an organic sacrificial reductant such as DIPEA or γ - terpinene (4 equiv) was used, another piece of RVC was employed as the anode.

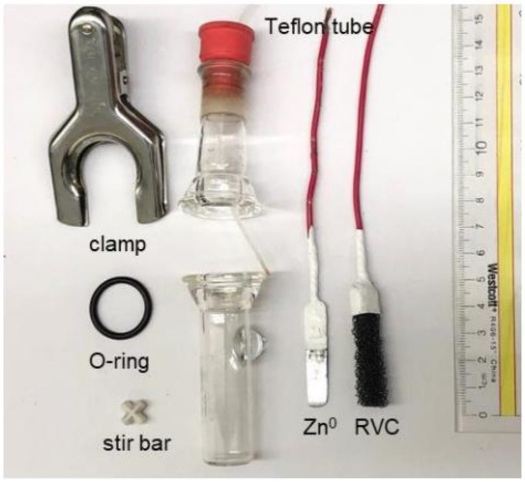

Figure S8. Picture of Setup

General Procedure for Production Rate Optimization Runs on Standard Flow Cell

Experimental Procedure

The standard cell (86 cm^2 electrode) was assembled according to procedure described above. To a clean dry glass bottle equipped with a stir bar was added: 1 equiv species **2**, 1.5 equiv 1-bromo-4-boc-piperidine (**1**), 1.6 equiv LiBr and 5 mol% (relative to species **2**) Ni catalyst synthetized according to procedure described above. Bottle was gently shaken to intermix solids prior to DMA addition. For runs using 0.635 cm C felt, 80 mL of DMA was added. For runs using 1.27 cm C felt, 120 mL of DMA was added. Solution was stirred and vigorously sparged with N_2 until complete dissolution. Manual breaking of clumps of LiBr with a glass stirring rod may be required. N_2 sparging occurred for duration of experiment. Upon complete dissolution of solids, tubing was connected to reactor and bottle containing to reactor solution. Reactor was filled at 100 mL min^{-1} (MasterFlex Peristaltic Pump, L/S 25 tubing PharmMed BPT material) and allowed to circulate for ~5 min upon reactor filling. Flow rate was then increased to 200 mL min^{-1}, leads were connected and electrolysis commenced at desired current density until 2.1 F mol^{-1} had passed. Cell current and voltage were recorded via a LabView program reading signals from an NI USB-6001 DAQ. Samples were taken every 30-60 min depending on reaction length and analyzed with 1H NMR with 1,3,5-trimethoxybenzene added as an external standard. NMR samples were quenched via exposure to air prior to analysis.

Standard Reactor Flow Setup

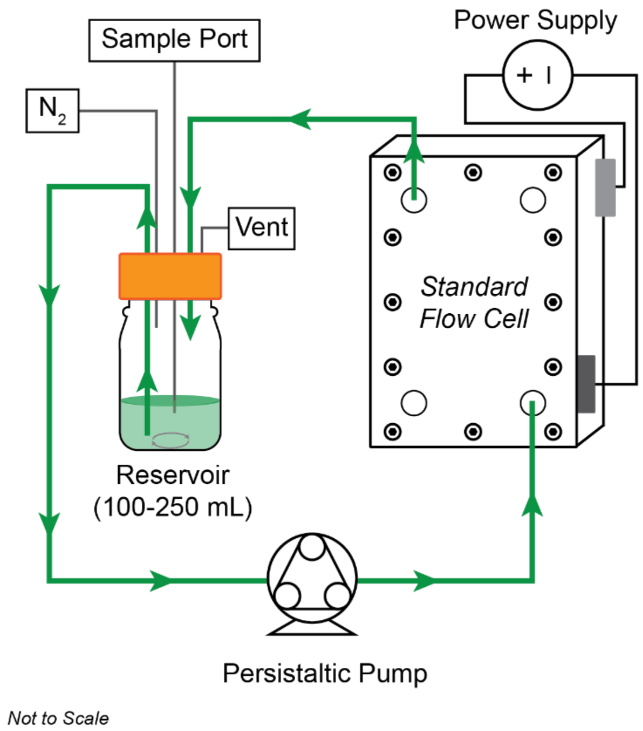

Figure S9. Process flow diagram for standard cell recirculation optimization studies

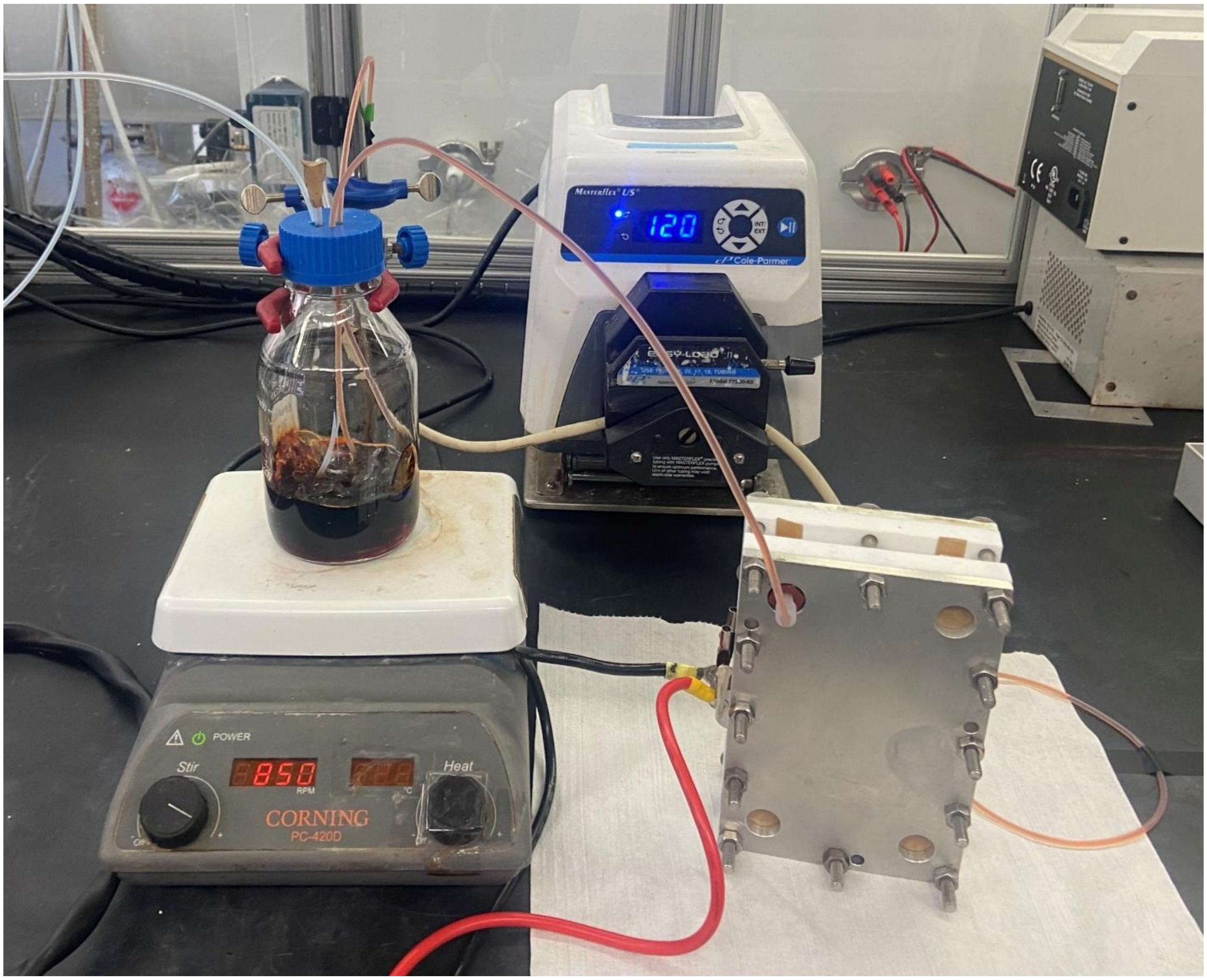

Figure S10. Image of standard cell electrolysis setup.

Flow Rate Selection

Table S1. Flow Rate Optimization in 86 cm^2 cell

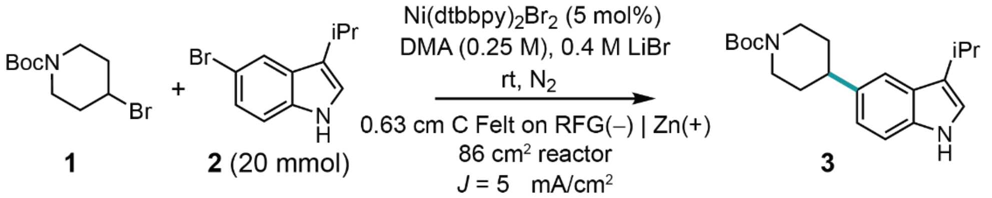

Flow Rate (mL min^{-1})	Conversion of **2**	^{1}H NMR % Yield of **3**
100	84%	73%
**200**	**91%**	**86%**
300	89%	86%

Nominal space time calculation

$$Nominal\ space\ time\ (min) = \frac{electrode\ volume\ (cm^3)}{flow\ rate\ (cm^3 min^{-1})} = \frac{86\ cm^2 * 1.27cm}{200\ (cm^3 min^{-1})} = \sim 0.54\ min$$

4 cm^2 Cell Flow Rate Selection

Scheme S1. Initial 10 mL min^{-1} attempt for multiday electrolysis

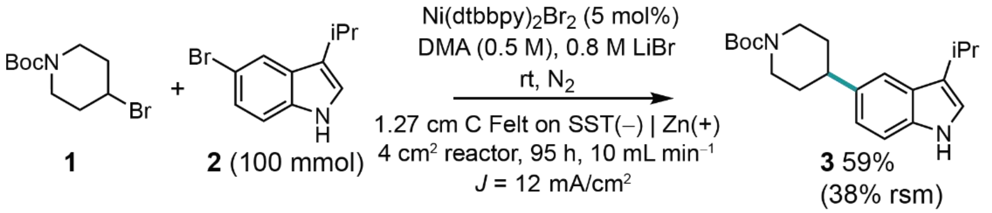

A flow rate of 10 mL min^{-1} to maintain the nominal space time of ~0.54 min, which featured precipitation of **6** and clogging of the cell. During the optimization of the multiday runs to eliminate precipitation of homodimer **6**, this flow rate was increased to 200 mL min^{-1} because it was initially hypothesized that the low flow rate was causing solid to settle in the reactor. However, it was later found that the concentration of alkyl bromide 1 governed the precipitation of species **6** and not the flow rate.

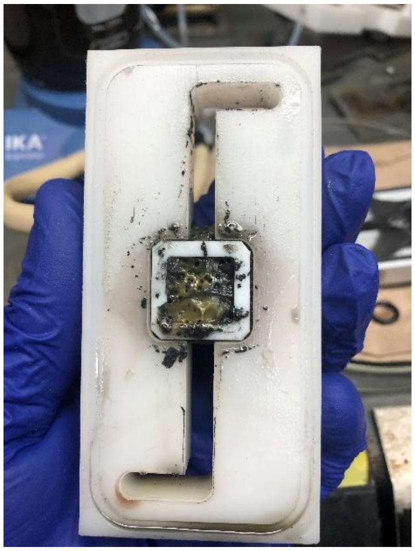

Figure S11. Image of the cell containing solid species **6** after a multiday electrolysis at 10 mL min^{-1}.

841 cm^2 Cell Flow Rate Selection

Initial prototype dimensions of the large reactor featured to an internal volume of roughly 0.93 L, which corresponded to a flow rate of 1.7 L min^{-1} to maintain the nominal space time of ~0.54 min. The operating flow rate was increased to 1.8 L min^{-1} because increasing the flow rate in the 86 cm^2 reactor did not negatively affect reaction performance, and it was possible that the relationship between nominal space time and reaction selectivity did not scale linearly. The final reactor dimensions had a slightly larger internal volume of 1.07 L. This larger internal volume increased the actual nominal space time value; however, this did not adversely affect reaction performance.

General Procedure for Multiday Electrolysis Using Small Cell (4 cm^2)

Experimental Procedure

A small cell (4 cm^2 electrode) was assembled according to the procedure described above. To a clean dry 500 mL glass bottle equipped with a stir bar was added: 1 equiv species **2**, 1.5 equiv 1-bromo-4-boc-piperidine (**1**), 1.6 equiv LiBr and 5 mol% (relative to **2**) Ni catalyst synthetized according to procedure described above. Bottle was gently shaken to intermix solids prior to DMA addition. DMA was then added to bottle. Solution was stirred and vigorously sparged with N_2 until complete dissolution. Manual breaking of clumps of LiBr with a glass stirring rod may be required. N_2 sparging occurred for duration of experiment. Upon complete dissolution of solids, tubing was connected to reactor and bottle containing to reactor solution. Reactor was filled at 20 mL min^{-1} (MasterFlex Peristaltic Pump, L/S 25 tubing PharmMed BPT material) and allowed to circulate for ~5 min upon reactor filling. Flow rate was then increased to 200 mL min^{-1}, leads were connected and electrolysis commenced at desired current density until 2.1 F mol^{-1} had been passed. At the end of the first day of electrolysis, the N_2 sparge line was pulled above liquid line of bottle to purge the headspace. Cell current and voltage were recorded with a LabView program reading signals from an NI USB-6001 DAQ when the Kepco or Keithley power supplies were used. Cell voltage and current were recorded using Gamry Framework software when a potentiostat was used. Samples were taken every 24 h and analyzed with 1H NMR with 1,3,5-trimethoxybenzene added as an external standard. NMR samples were quenched via exposure to air prior to analysis.

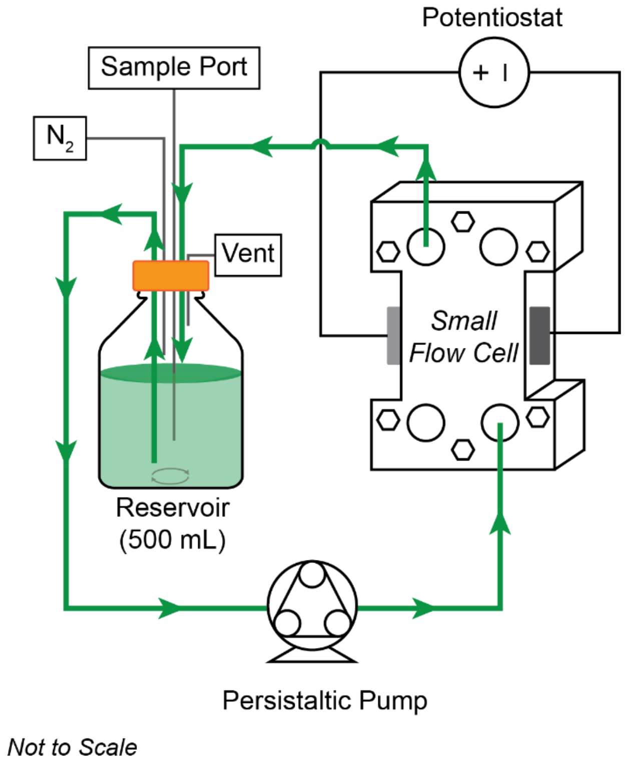

Figure S12. Process flow diagram for multiday electrolysis.

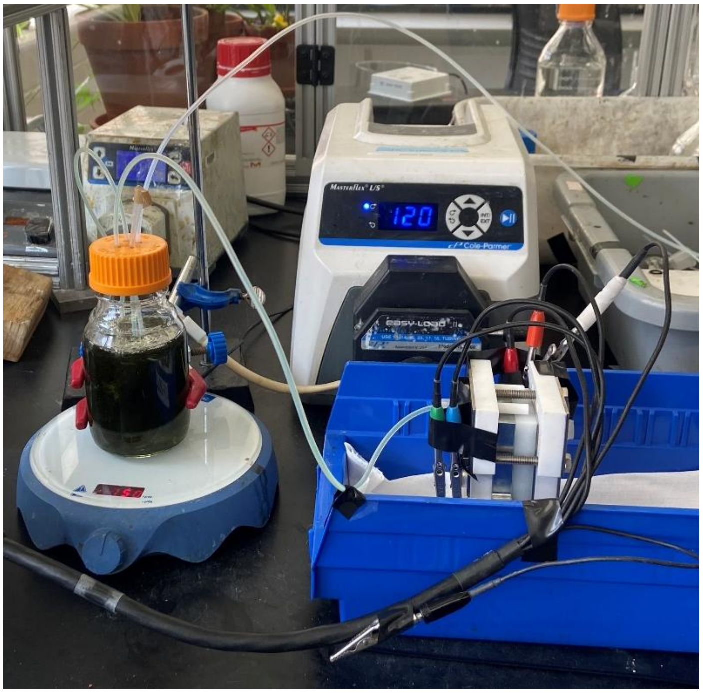

Figure S13. Image of multiday setup running.

Solid Formation During Multiday Electrolysis

Scheme S2. Reaction condition for multiday electrolysis that featured precipitation of species **6** (Entry 1 **Table 3**).

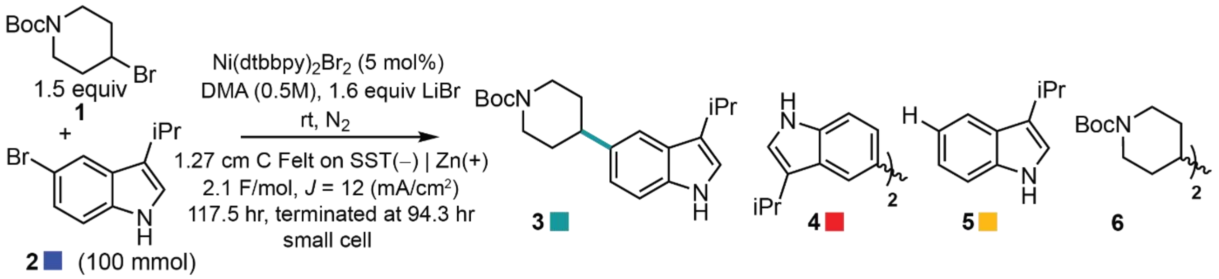

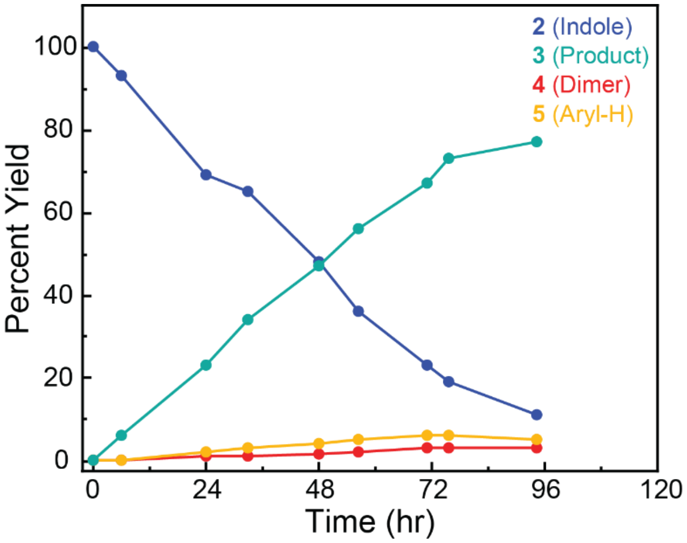

Figure S14: Time series of long term run that featured the onset of solid precipitation at 71 h. At ~72 h, electrolysis was paused, solution was filtered and then reintroduced into system. Reaction was terminated at 94.3 h due to high cell voltage (see **Fig S15**).

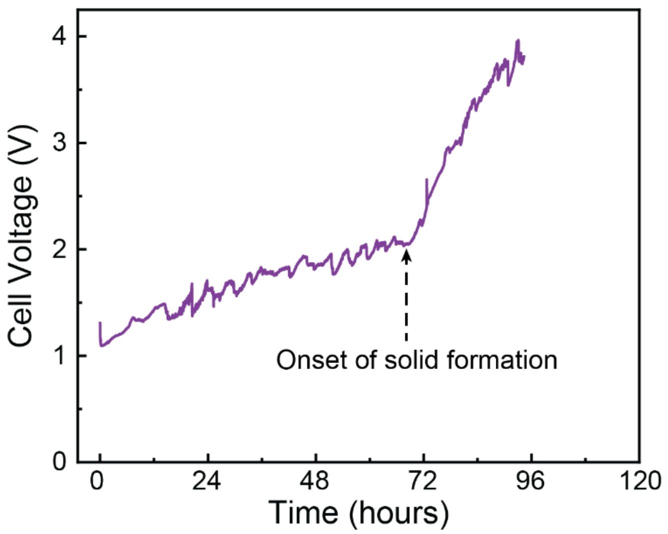

Figure S15: Voltage trace of entry 1, **Table 3** (reaction conditions shown in **Scheme S1**). Onset of solid formation at 71 h corresponded to the increase in cell voltage. The high cell voltage value necessitated reaction termination at 94.3 h.

Scheme S3. Reaction conditions for 100 mmol experiment conducted on standard cell to compare to long term run (entry 1, **Table 3**).

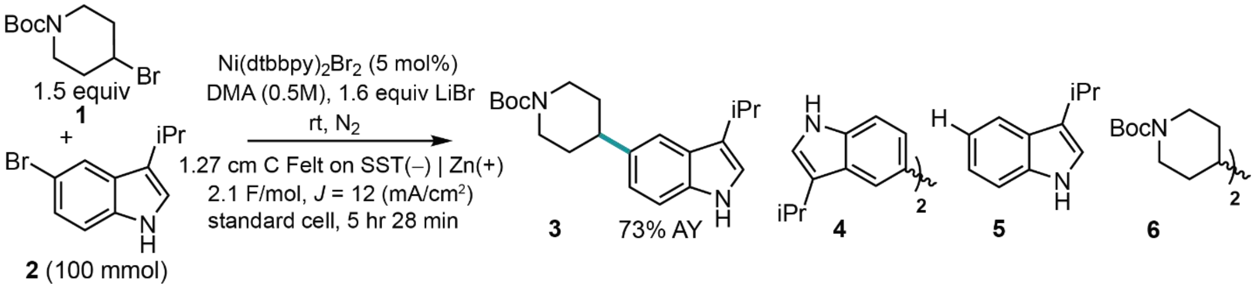

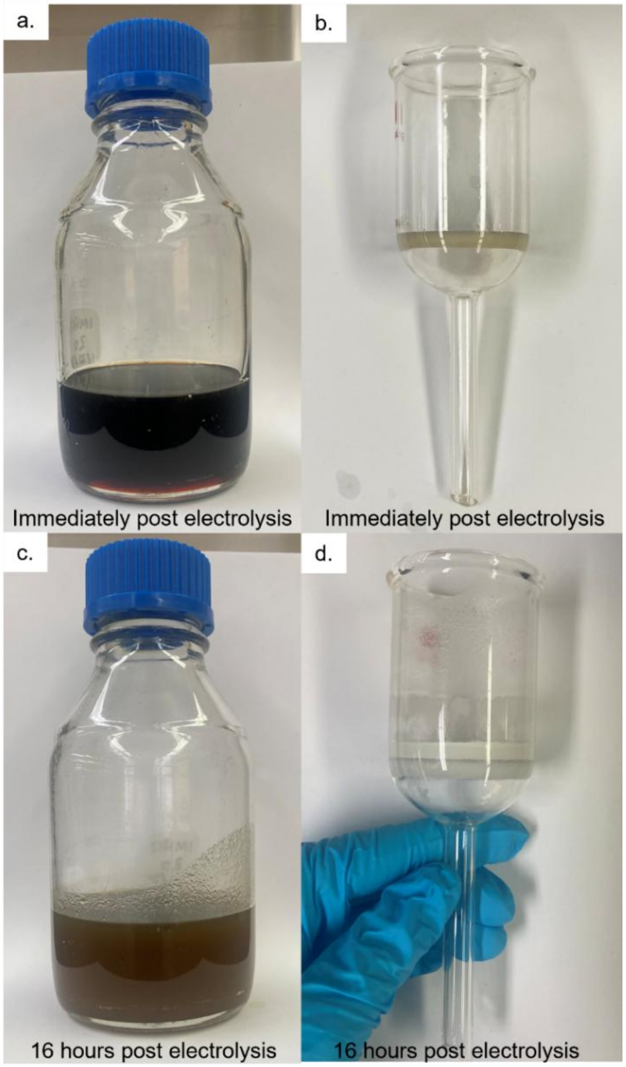

Figure S16. Evidence of slow precipitation kinetics. **(a)** Reaction solution immediately post electrolysis using standard cell, 100 mmol scale, 0.5M species **2**, 12 mA cm^2 **(b)** Filter after filtration of solution in **(a)**. **(c)**. Reaction solution **(a)** after having sat for 16 h **(d)** Filter after filtration of solution depicted in **(c)**, white layer on top of frit solids (washed with DMA/H_2O prior to photo).

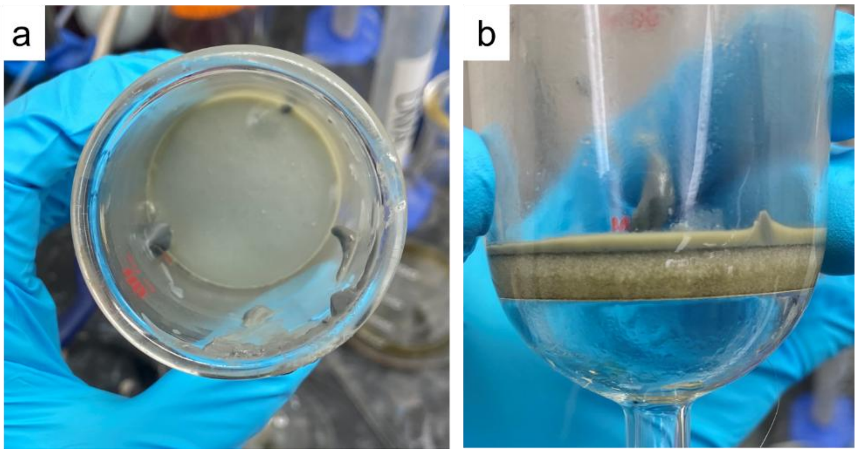

Figure S17. Images of unwashed solid from Entry 1 in **Table 3** after solution was filtered at ~72 h. **(a)** overhead view of filter **(b)** side view of filter.

Table S2. ICP-OES Data for Zn and Ni content of precipitated solid

Element	ppm Found via ICPOES	wt% in Sample
Ni	0.46	0.08
Zn	11.39	2.09

Sample size: 19.2 mg diluted with 35.1561 g of water.

Based on the low Ni and Zn content, this provided evidence that the solid was organic. Mass spectrometry was conducted and the primary mass fragment corresponded to the dimer of the alkyl species **1**.

MS (EMM / ESI): *m/z* $[M + Na]^+$ calcd for $C_{20}H_{36}N_2O_4$, 391.2567; found, 391.2566 (0.2 ppm error).

Successful Multiday Electrolysis Performance

Scheme S4. Reaction conditions for successful multiday run conducted with a starting material **2** concentration of 0.3M and electrolyzed at 10 mA cm^2.

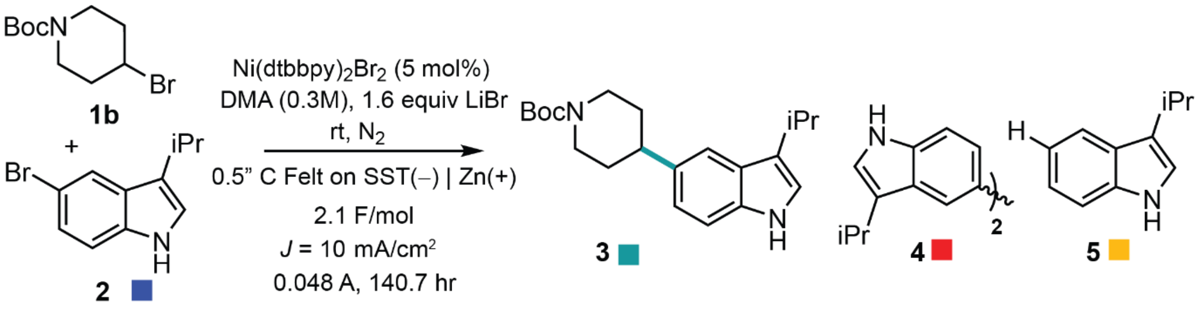

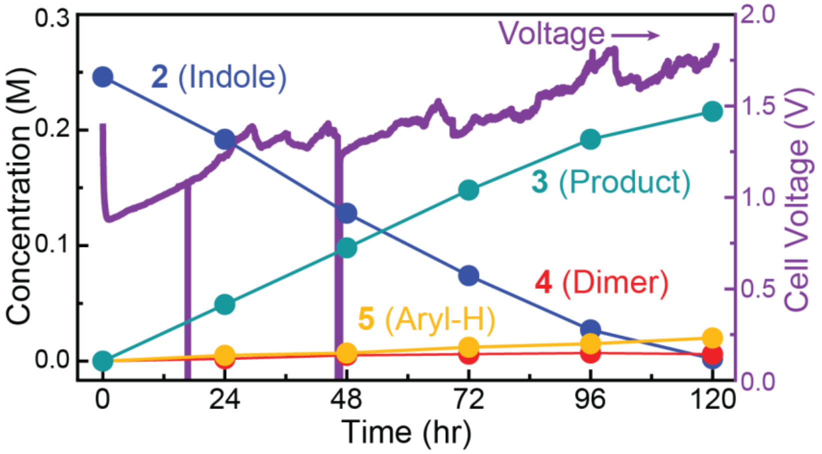

Figure S18. Time series and voltage trace for successful multiday electrolysis (entry 4 **Table 3**). Note that full conversion of starting material **2** was achieved at 120 h, but electrolysis was continued for 140.7 h until 2.1 F mol^{-1} of charge had been passed. Only the first 120 h shown here, see next section for full voltage trace and time series (**Fig S19**). To demonstrate robustness to interruptions in electrolysis, connection was briefly interrupted at 16.6 h and 29.75 h – this did not negatively impact the overall result of the electrolysis.

Multiday Electrolysis Finishing Before 2.1 F mol^{-1}

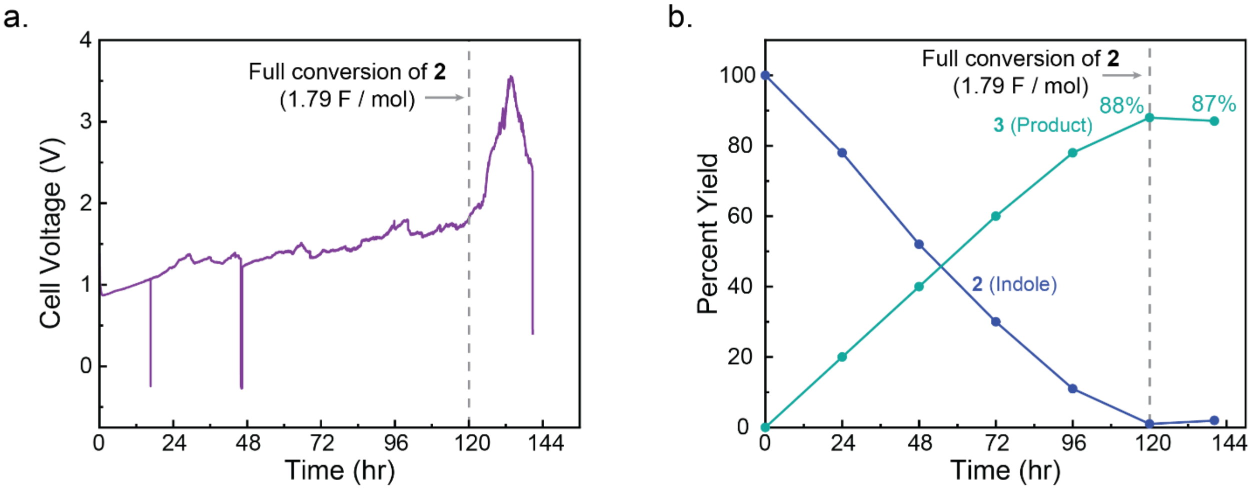

Figure S19. Full voltage trace and time series for entry 4 **Table 3**. **(a)** Voltage trace – voltage behavior after 120 h is due to starting material 2 having been fully consumed. **(b)** Time series of starting material **2** and product **3** for the entire 140.7 h electrolysis. Note that continuing electrolysis beyond full consumption of starting material **2** did not result in degradation of product **3**.

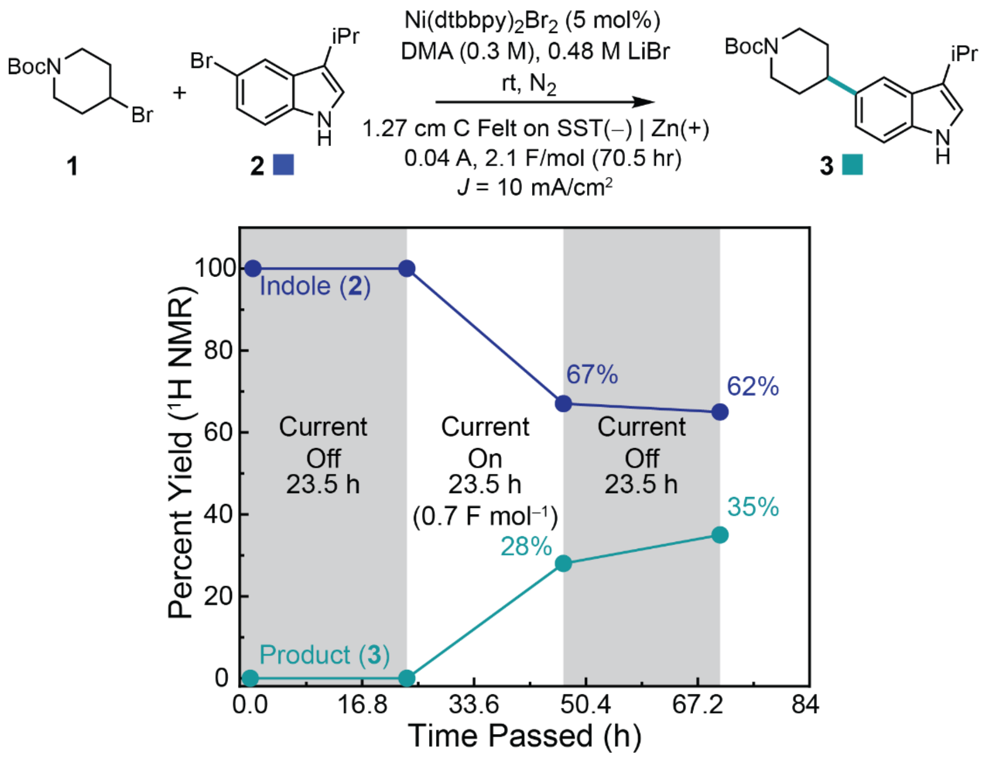

Figure S20. Current off/on/off experiment to probe non-electrochemical pathway. First 23.5 h time period conducted under no current, no product formation observed. 28% product formed during next 23.5 h (0.7 F mol^{-1}) under an applied current. The last 23.5 hour time period conducted in the absence of current resulted in 7% product **3** formation. Reaction conducted in 4 cm^2 cell with 50 mmol indole. Because initial exposure to current was needed to enable product formation when the current was turned off, we hypothesize that the Zn anode dissolution could form a high surface area of Zn(0), some of which could reduce the Ni catalyst non-electrochemically.

General Procedure for 400 mmol Large Cell Validation

Experimental Procedure

The large cell (841 cm^2 electrode) was assembled according to the procedure described above. To a clean dry 2 L glass bottle equipped with a stir bar was added: 1 equiv species **2** (400 mmol, 95 g), 1.5 equiv 1-bromo-4-boc-piperidine (**1**) (600 mmol, 159 g), 1.6 equiv LiBr (640 mmol, 56 g) and 5 mol% (relative to species **2**) Ni catalyst (20 mmol, 15.1 g) synthetized according to the procedure described above. The bottle was gently shaken to intermix solids prior to DMA addition. 1.2 L of DMA was then added to bottle. The solution was stirred and vigorously sparged with N_2 until complete dissolution. Manual breaking of clumps of LiBr with a glass stirring rod may be required. N_2 sparging occurred for the duration of the experiment. Upon complete dissolution of solids, reactor was filled at 1 L min^{-1} (MasterFlex Peristaltic Pump, L/S 18 tubing PharmMed BPT material) and allowed to circulate for ~5 min. Reciculation vial was changed to 500 mL bottle for ease of sample collection. Recirculation vial was placed in ice bath, and the tubing was connected to reactor and bottle containing to reactor solution. Flow rate was then increased to 1.8 L min^{-1}, leads were connected (12 AWG wire) and electrolysis commenced at 8.41 A for 2.1 F mol^{-1} (162 min). Power supply used was Kepco BOP 20/20. Cell current, voltage, recirculation vial temperature, outlet line temperature, reactor inlet pressure, and reactor outlet pressure were recorded with a LabView program reading signals from an NI USB-6001 DAQ. Additional ice was added to the water bath every 30 min. Samples were taken every 30 min and analyzed with 1H NMR with 1,3,5-trimethoxybenzene added as an external standard. NMR samples were quenched via exposure to air prior to analysis.

Cell Pressure and Temperature Data for 400 mmol Validation Run

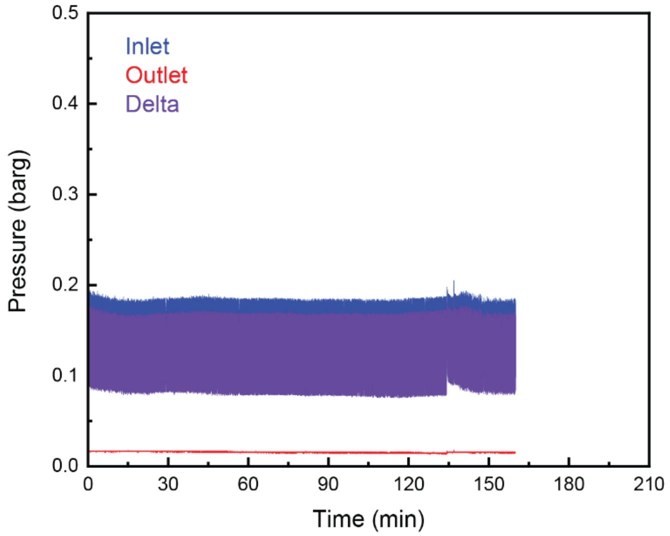

Figure S21. Pressure transducer trace and pressure drop for 400 mmol large cell validation run. Inlet pressure sensor shown in blue, outlet pressure sensor shown in red, pressure differential show in purple.

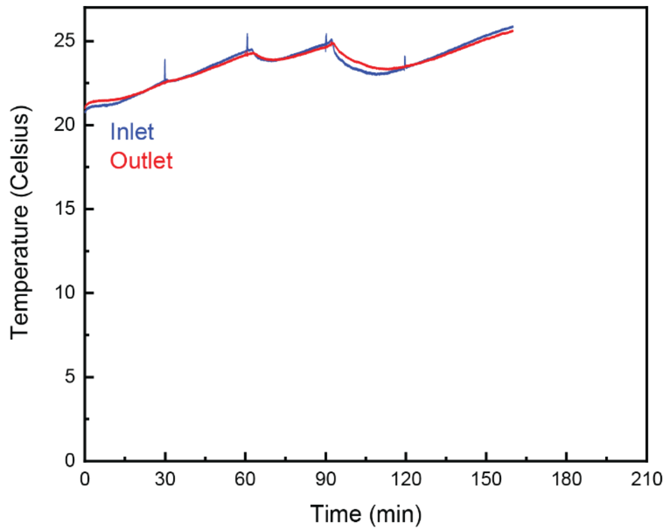

Figure S22. Reactor inlet (blue) and outlet temperature (red) trace for 400 mmol large cell validation run. Spikes in inlet temperature every 30 min due to probe being briefly removed to enable a sample to be taken. Additional ice was added to the recirculation ice bath every 30 min.

General Procedure for 1 kg Scale Run

Experimental Procedure

The large cell (841 cm^2 electrode) was assembled according to the procedure described above. To a clean dry 20L jacketed reactor vessel equipped with an overhead impeller were added: 1 equiv species **2** (2.92 mol, 691 g), 1.5 equiv 1-bromo-4-boc-piperidine (**1**) (4.38 mol, 1153 g), 1.6 equiv LiBr (4.67 mol, 406 g) and 5 mol% (relative to **2**) Ni catalyst (0.146 mol, 110.3 g) synthetized according to the procedure described above. Because 1-bromo-4-boc-piperidine solid was difficult to remove from bottle, DMA was used to dissolve the 2 x 500 g bottles and added to the rest of the solids in the reactor (remaining 168 g of **1** was added in solid form). In close proximity to an open fume hood to capture DMA vapor, the remaining DMA (9.73 L total) was slowly added to recirculation vessel. Recirculation vessel was then stirred at ~190 rpm overnight under N_2 sparging to allow complete dissolution of solids. The next day, tubing was connected to reactor and stir rate was decreased to ~150 rpm. Cooling jacket was then filled with pure H_2O and recirculated with a Neslab RTE 7 recirculating chiller set between 18.5 and 20°C. Reactor was filled at 1 L min^{-1} (MasterFlex Peristaltic Pump, L/S 18 tubing PharmMed BPT material) and allowed to circulate for ~5 min. Flow rate was then increased to 1.8 L min^{-1} (in 100 mL min^{-1} intervals), leads were connected (12 AWG wire) and electrolysis commenced at 8.41 A for 2.1 F mol^{-1} (19.55 h). Power supply used was Kepco BOP 20/20. Cell current, voltage, recirculation vial temperature, outline line temperature, reactor inlet pressure, and reactor outlet pressure were recorded with a LabView program reading signals from an NI USB-6001 DAQ. Chiller temperature setpoint was adjusted to maintain recirculation vessel temperature at room temperature (~20°C). Samples were taken every 30 min and analyzed with 1H NMR with 1,3,5-trimethoxybenzene added as an external standard. NMR samples were quenched via exposure to air prior to analysis.

Cell Pressure and Temperature Data for 1 kg Scale

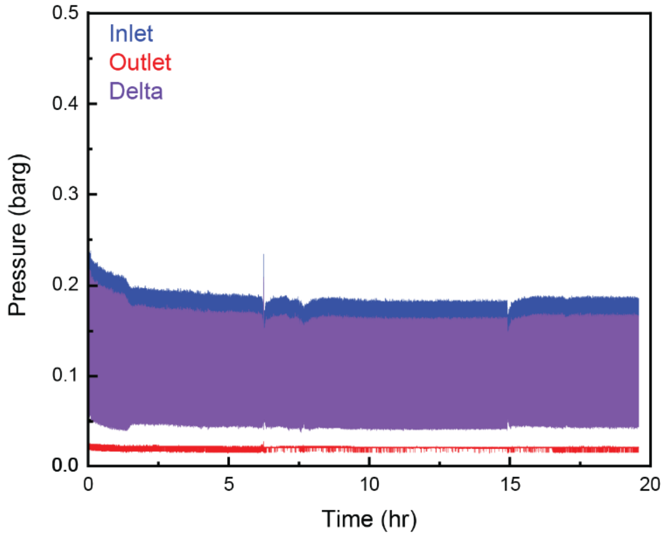

Figure S23. Pressure transducer trace and pressure drop for 1 kg scale synthesis. Inlet pressure sensor shown in blue, outlet pressure sensor shown in red, pressure differential show in purple.

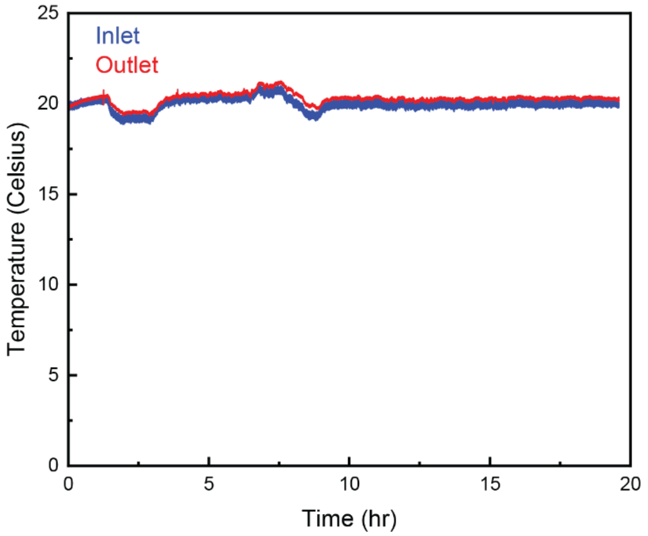

Figure S24. Reactor inlet (blue) and outlet (red) temperature trace for kilogram scale synthesis.

Voltage Trace Comparison

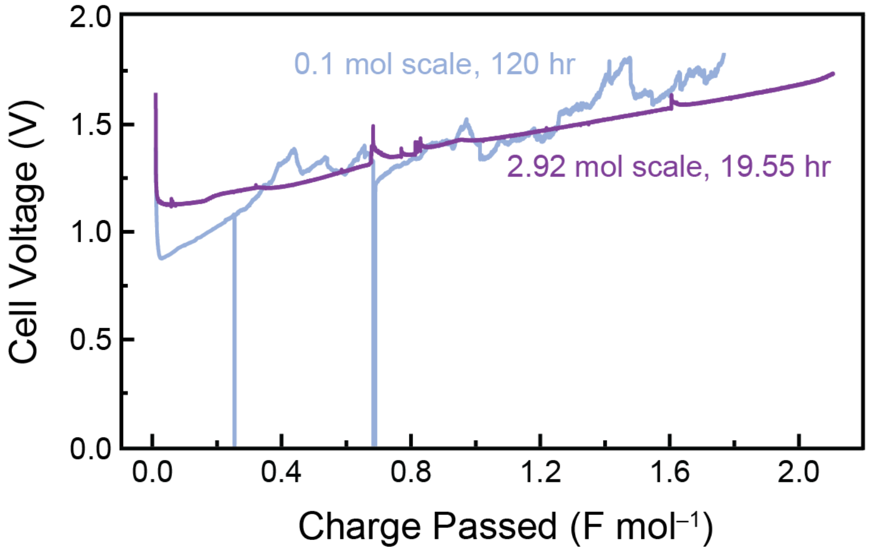

Figure S25. Overlay of cell voltage traces from multiday electrolysis (entry 4 **Table 3**) and kilogram scale synthesis plotted relative to the charge passed. 0.1 mol scale multiday run terminates prior to 2.1 F mol^{-1} to only show voltage trace relevant to when the reaction was occurring (see **Figure S19** for more information).

Estimating Expected Change in Zn Anode Thickness

For every mole of starting material **2**, 2.1 F mol^{-1} of charge are passed. The anodic reaction is shown below. Because 2 electrons per mole of zinc are released, this corresponds to 1.05 mol of Zn required per mol of starting material **2**.

$$Zn^0 \rightarrow Zn^{2+} + 2e^-$$

To determine the estimated change in thickness of the Zn anode, the following conversion was used:

$$\Delta Zn\ (cm) = mol\ Zn * mw\ Zn\ \left(\frac{g}{mol}\right) * \frac{1}{\rho_{Zn}}\left(\frac{cm^3}{g}\right) * \frac{1}{electrode\ area}\left(\frac{1}{cm^2}\right)$$

Where ρ_{Zn} is the density of solid Zn with a value of 7.14 g cm^{-3} and the electrode area is equal to the area of the C felt cathode for the respective cell (see **Fig S4**).

Power and Energy Requirement Calculations for 100 kg Production Campaign

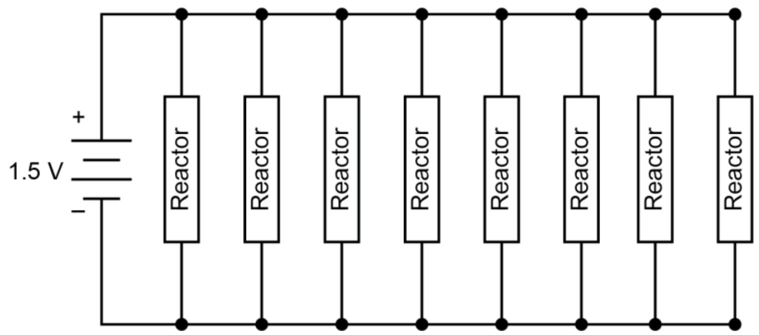

Figure S26. Representative circuit diagram for 8 reactors running in parallel with an average cell voltage of 1.5 V for a 100 kg production campaign conducted in 2 x 50 kg batches. Note that actual operation would be conducted under constant current conditions (which may require reactors to operate in series).

$$P = IV$$
$$P_{tot} = \sum P_{cell}$$
$$Energy = P * time$$

Table S3. Calculation of total power requirement for the reactors operating in parallel

Cell Number	J (mA cm^{-2})	V_{avg}	I (A)	P (W)
1	10	1.5	8.41	12.615
2	10	1.5	8.41	12.615
3	10	1.5	8.41	12.615
4	10	1.5	8.41	12.615
5	10	1.5	8.41	12.615
6	10	1.5	8.41	12.615
7	10	1.5	8.41	12.615
8	10	1.5	8.41	12.615
Total				**101**

Table S4. Calculating electrical energy required for the 100 kg production campaign

Batch Number	P (kW)	Length (h)	Energy (kWh)
1	0.1	122.4	12.24
2	0.1	122.4	12.24
Total			**24.48**

Product Isolation Procedure

A 1L portion of the DMAc solution of **3** (7.6 wt%) containing 75.9 g of **3** was filtered through a 0.45 mm PTFE filter into a 5 L overhead stirred reactor and 2 L of 2-methyltetrahydrofuran (MeTHF, 26 mL/g **2**) followed by 2 L of water (26 mL/g) was added. The mixture was stirred for 30 min, the layers allowed to settle and then the lower aqueous layer was removed. To this was added 1 L of water (13 mL/g **2**) and the mixture was stirred for 30 min, the layers allowed to settle and then the lower aqueous layer was removed. To this was added 0.5 L of a 10 wt% aqueous solution of citric acid (6 mL/g **2**) and the mixture was stirred for 30 min, the layers allowed to settle and then lower aqueous layer was removed. The upper brown organic layer was transferred to a 2L flask and concentrated at 40°C on the rotovap to a low volume. Added 210 mL isopropanol (IPA, 3 mL/g **2**) and concentrated to a low volume. Repeated addition of 210 mL IPA (3 mL/g **2**) twice and then added 210 mL IPA (3 mL/g **2**) before transferring solution containing 72.6 g of **3** to a 1 L overhead stirred reactor. Added 70 mL H_2O over 1h then seeded with 70 mg authentic **2** (1 wt%). Aged for 3 h then added 140 mL H_2O to the tan slurry over 6h. Held for 12h then filtered, washed solids with 70 mL 1:1 IPA:H_2O (1 mL/g **2**) followed by 70 mL heptanes (1 mL/g **2**). Dried under N_2/vacuum sweep to obtain 57.6 g of **3** off-white solids (76% recovery, 62% isolated yield, 99.2 LC-AP (215 nm), 98.6 wt% QNMR).[1] mp 126 °C (DSC). ^{1}H NMR ($CDCl_3$, 500 MHz): δ 7.91 (br s, 1H), 7.47 (br s, 1H), 7.29 (d, J = 8.4 Hz, 1H), 7.05 (dd, J = 8.4, 1.6 Hz, 1H), 6.95 (d, J = 1.6 Hz, 1H), 4.28 (m, 2H), 3.20 (septd, J = 6.9, 0.9 Hz, 1H), 2.85 (td, J = 13.0, 2.5 Hz, 2H), 2.76 (tt, J = 12.2, 3.5 Hz, 1H), 1.89, (m, 2H), 1.72 (qd, J = 12.6, 4.2 Hz, 2H), 1.51 (s, 9H), 1.37 (d, J = 6.9 Hz, 6H). $^{13}C\{^1H\}$ NMR (100 MHz, $CDCl_3$, 25°C): δ 155.0, 136.6, 135.4, 126.9, 123.8, 121.1, 119.6, 116.8, 111.0, 79.3, 44.6, 43.0, 34.0, 28.5, 25.4, 23.3. IR (SS ATR): 1654, 1480, 1433, 1368, 1272, 1239, 1228, 1158, 1127, 1076, 1016 cm^{-1}. HRMS (ESI) m/z: $[M+H]^+$ Calcd for $C_{21}H_{30}N_2O_2$ 343.2380; found 343.2385 (1.35 ppm error).

Table S5. Purity Data for Isolation Streams

Entry	Sample	Conv (%)[a]	**3** *vs* **4** (%)[b]	**3** *vs* **5** (%)[b]
1	DMA stream	>99.9	95.0	93.0
2	Isolated	>99.9	99.7	99.5

a. Conversion based on LC-UV area counts (ACs) at 215 nm by CONV = 100(AC(**3** + **4** + **5**)/(AC(**2** + **3** + **4** + **5**)) not adjusted for response factors.

b. Selectivity based on LC-UV area counts (ACs) at 215 nm by 100(AC(**3**)/(AC(**3**) + AC-(**impurity**)) not adjusted for response factors.

tert-butyl 4-(3-isopropyl-1H-indol-5-yl)piperidine-1-carboxylate (**3**)

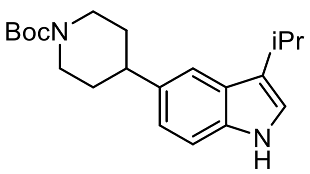

Spectra of Compounds

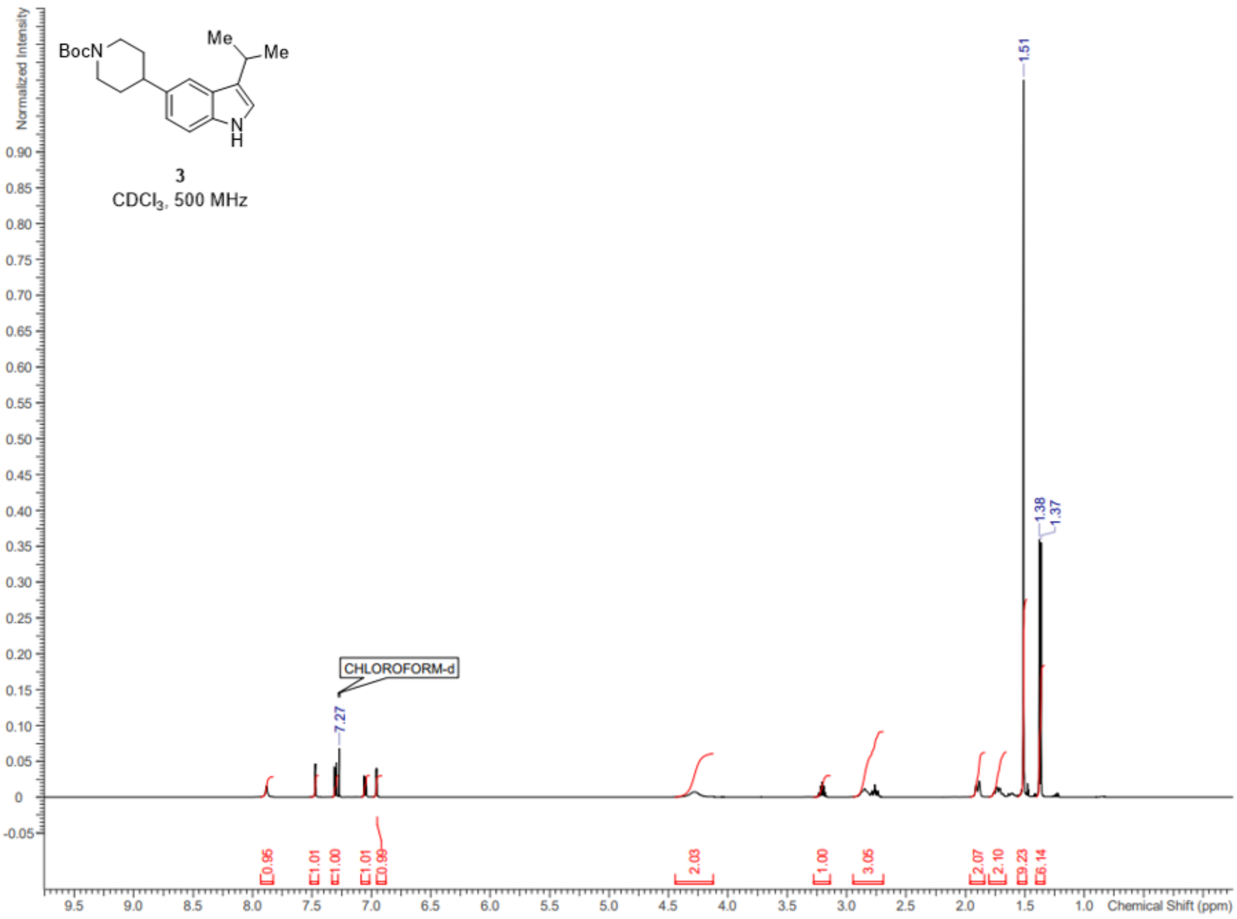

^{1}H NMR of compound **3** (500 MHz, $CDCl_3$)

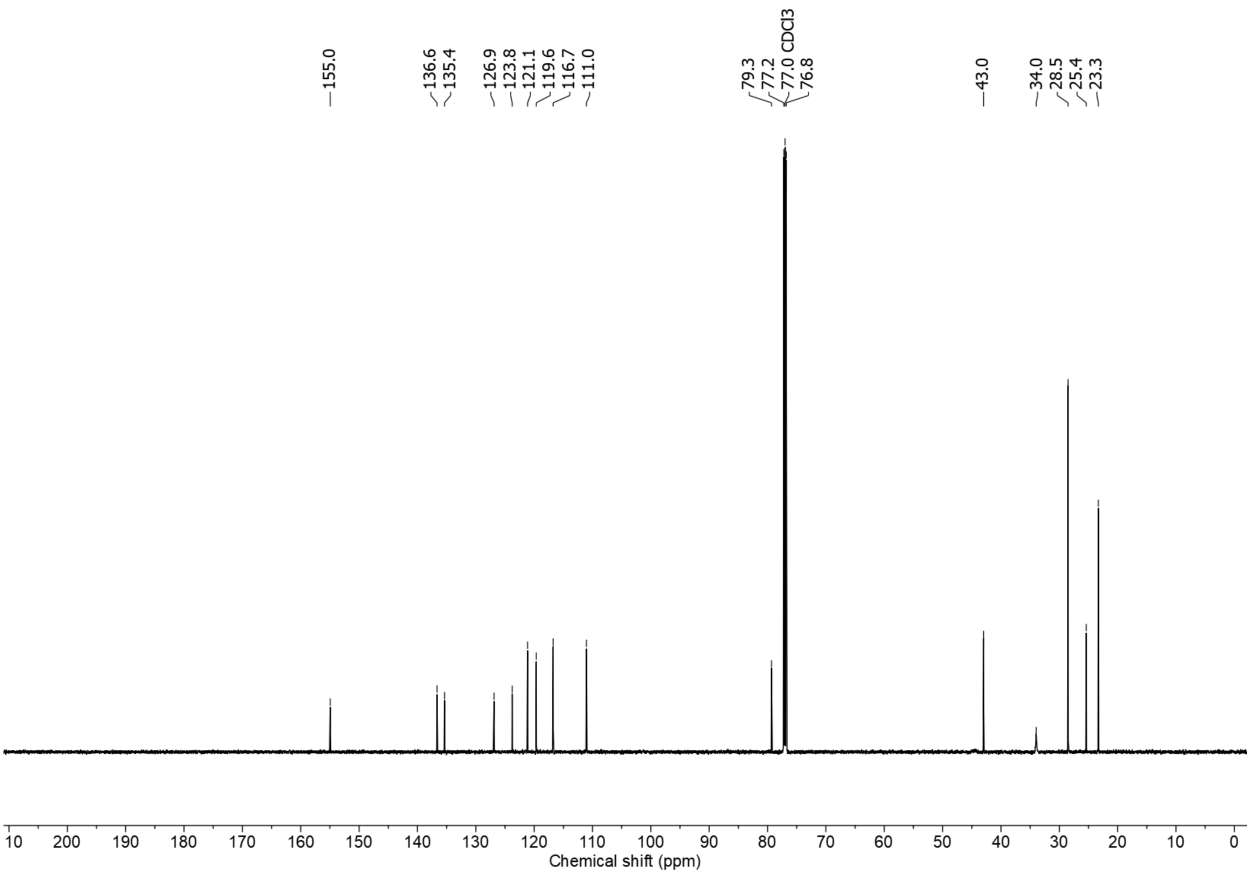

^{13}C NMR of compound **3** (152 MHz, $CDCl_3$)

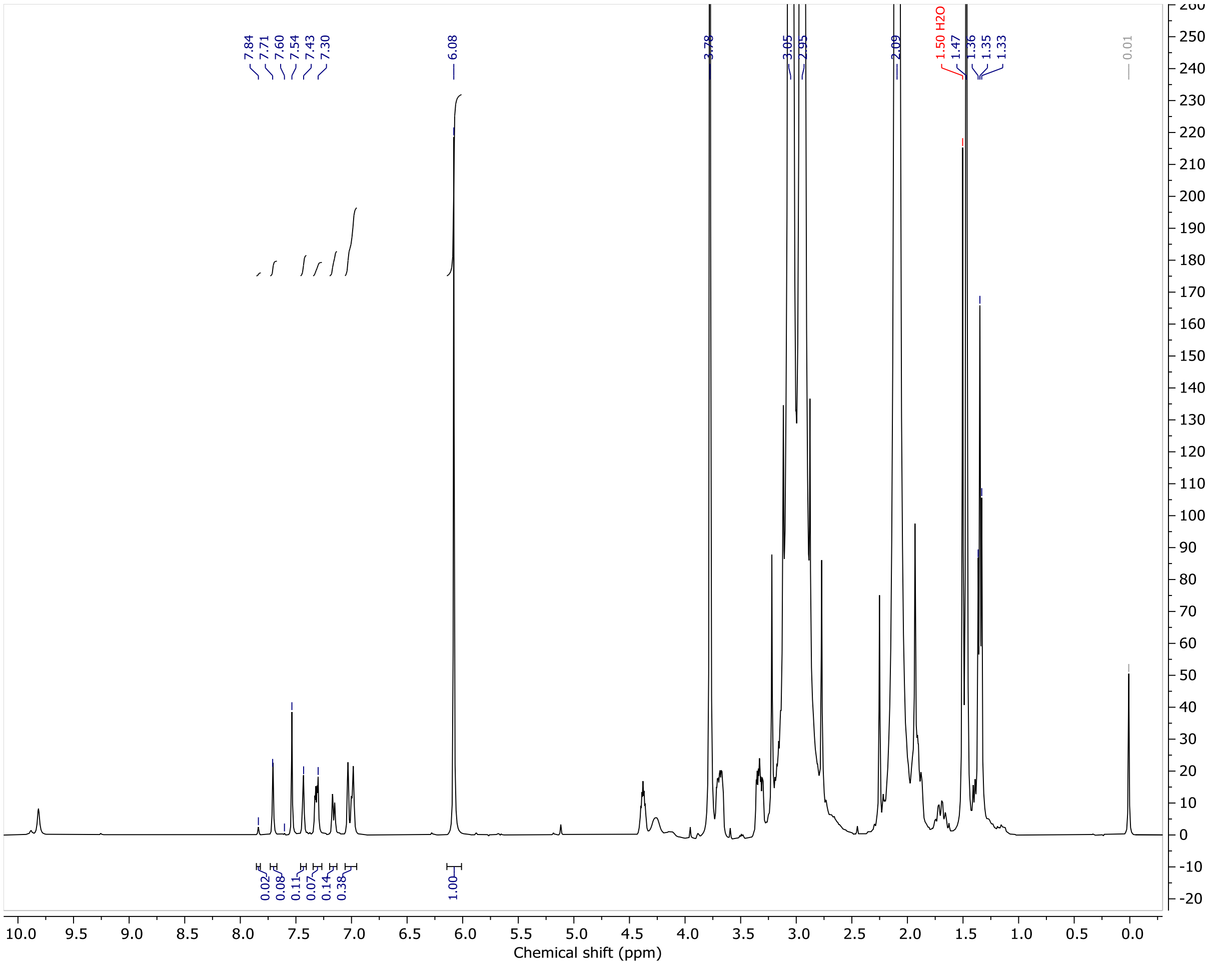

^{1}H NMR of crude reaction mixture (600 MHz $CDCl_3$)

Technical Specifications and Drawings

Table S6. Key Reactor Design Parameters

Reactor Size	Electrode Area (cm^2)	Electrode Height (cm)	Electrode Width (cm)	Wetted geometry (cm^3)	Approx. Interelectrode gap (mm)
Small	4	2	2	2 x 2 x 1.27	~2.5[b]
Standard	86	12[a]	8[a]	12 x 8 x 1.27	~2[b]
Large	841	29	29	29 x 29 x 1.27	~3.5[b]

a. Maximum height and width, electrode is an elongated hexagon, see Figures S4, S5
b. Reactors were designed to minimize the interelectrode gap without shorting the reactor. Each nominal 0.5" felt is closer to 3/8" in operation, thus this gap, plus the gap of the relevant meshes or caps (see detailed assembly instructions) were used to approximate the interelectrode gap. Compression from tightening the reactor likely makes this distance smaller.

Standard (86 cm^2) Cell Relevant Drawings

"Standard (86 cm^2) Cell Clamp"

"Standard (86 cm^2) Cell A Plate"

"Standard (86 cm^2) Cell Current Collector / Zn Anode"

"Standard (86 cm^2) Cell C Felt Frame"

"Standard (86 cm^2) Cell 'B' Plate"

Small (4 cm^2) Cell Relevant Drawings

"Small (4 cm^2) Cell Clamp"

"Small (4 cm^2) Cell A Plate"

"Small (4 cm^2) Cell Current Collector / Zn Anode"

"Small (4 cm^2) Cell C Felt Frame"

"Small (4 cm^2) Cell 'B' Plate"

Large (841 cm^2) Cell Relevant Drawings

"Large (841 cm^2) Cell Assembly"

"Large (841 cm^2) Cell Flow Plate"

"Large (841 cm^2) Cell Current Collector / Zn Anode / Clamp"

"Plastic Cap for C Felt"

Standard (86 cm²) Cell Clamp Plate

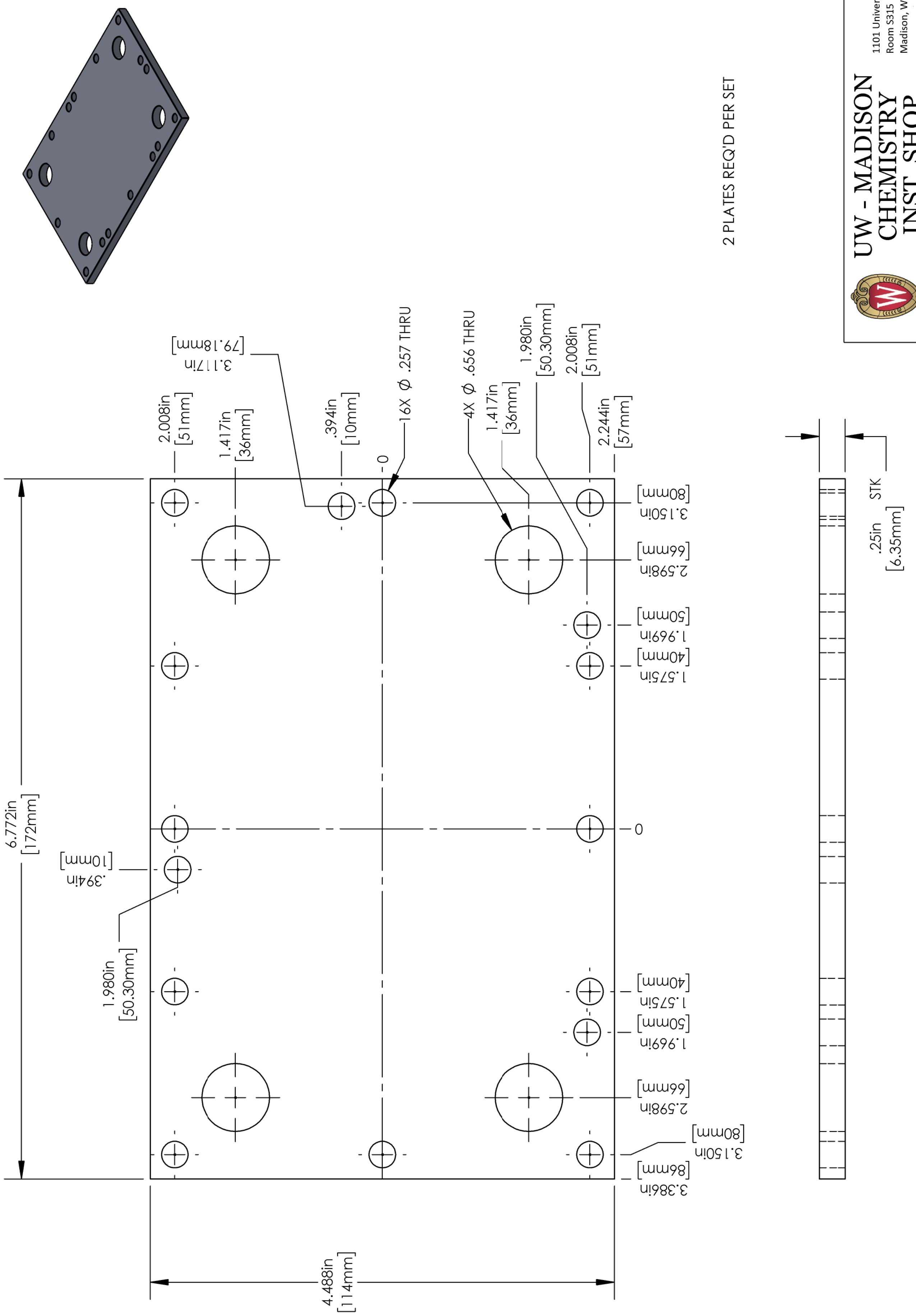

2 PLATES REQ'D PER SET

UW - MADISON
CHEMISTRY
INST. SHOP
1101 University Ave
Room S315
Madison, WI 53706

MAT: ALUMINUM

UNLESS OTHERWISE SPECIFIED:
DIMENSIONS ARE IN INCHES
TOLERANCES: ANG: ±1°
.XX ±.010 .XXX ±.005

THIRD ANGLE PROJECTION

Standard (86 cm^2) Cell "A" Plate

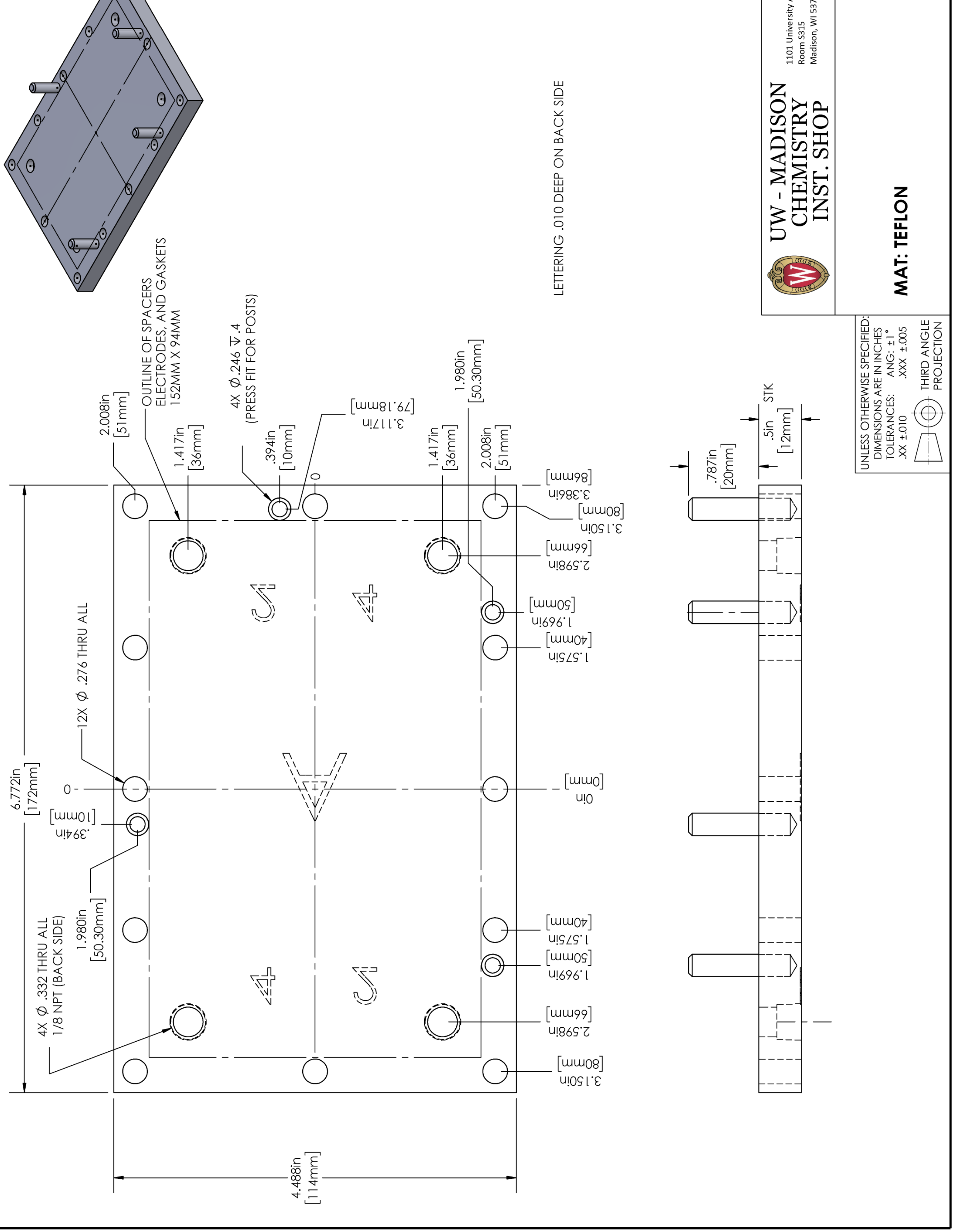

Standard (86 cm^2) Cell Current Collector / Zn Anode

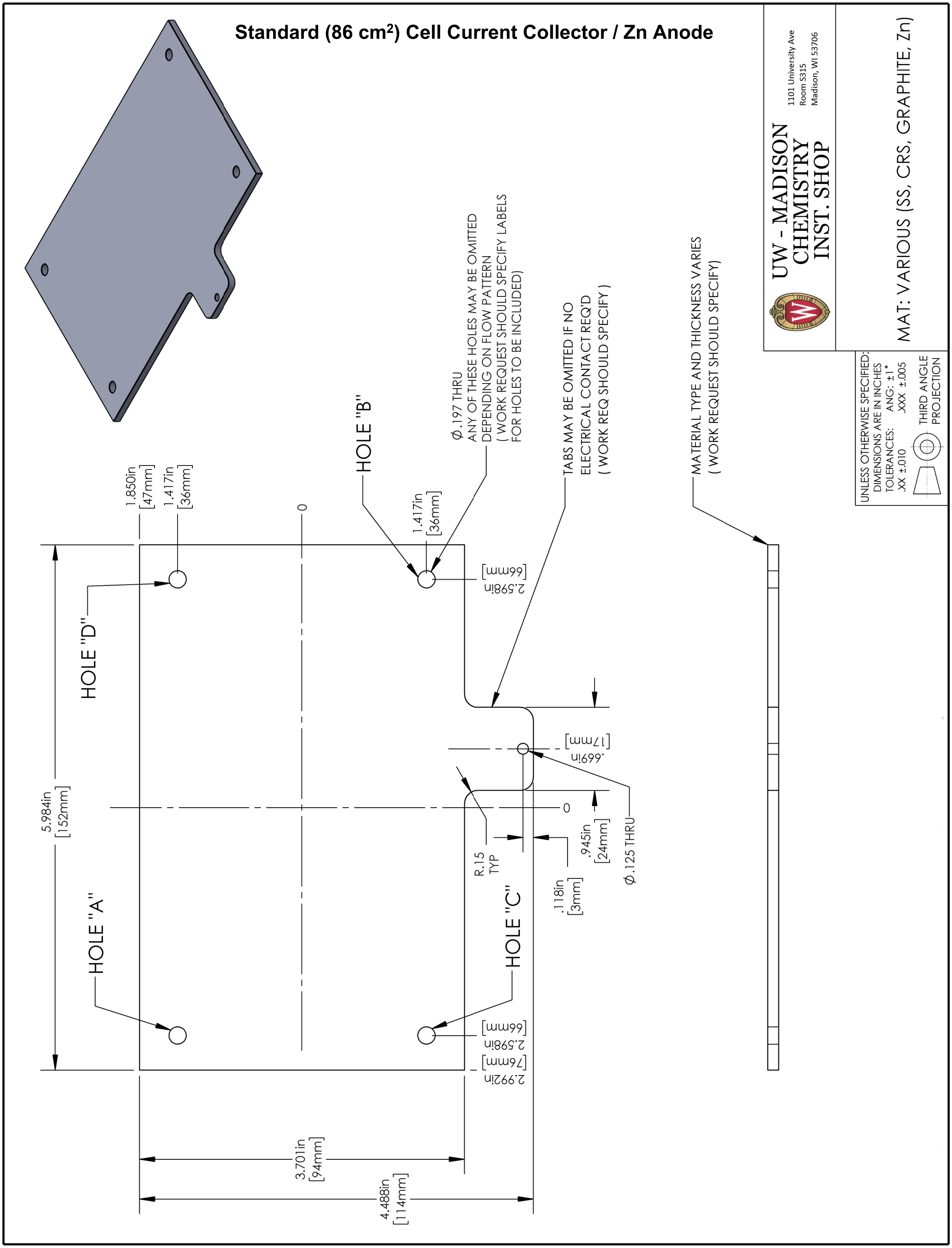

Standard (86 cm^2) Cell C Felt Frame

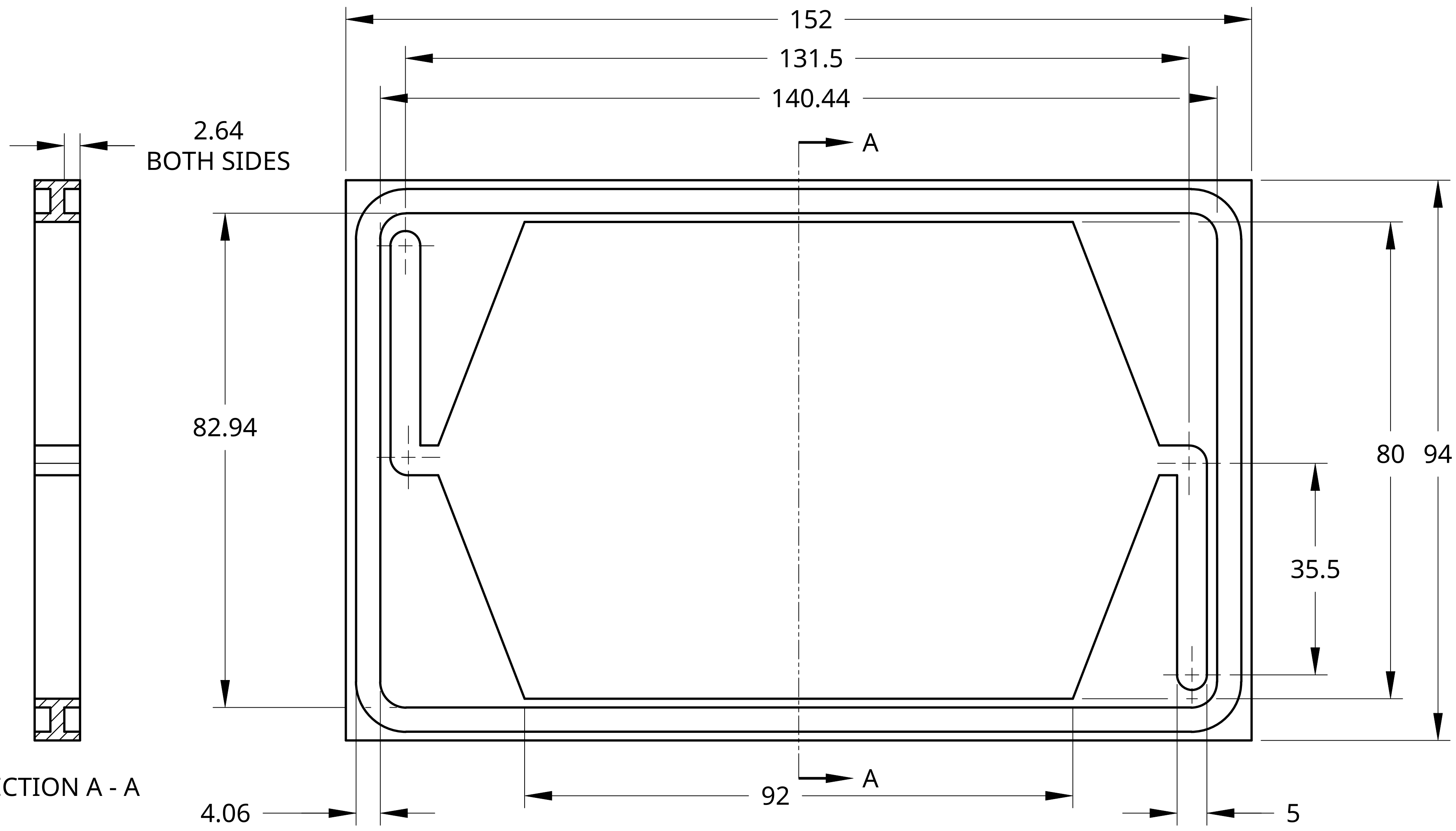

UNLESS OTHERWISE SPECIFIED, DIMENSIONS ARE IN MILLIMETERS<br>ANGULAR = ± °		NAME	DATE	
	DRAWN	JESSE DARLEY	04/07/2026	
SURFACE FINISH	CHECKED			TITLE
	APPROVED			
DO NOT SCALE DRAWING				
BREAK ALL SHARP EDGES AND REMOVE BURRS				SIZE A / DWG NO. / REV.
THIRD ANGLE PROJECTION	MATERIAL	FINISH		SCALE 1:1 / WEIGHT / SHEET 1 of 1

Standard (86 cm^2) Cell "B" Plate

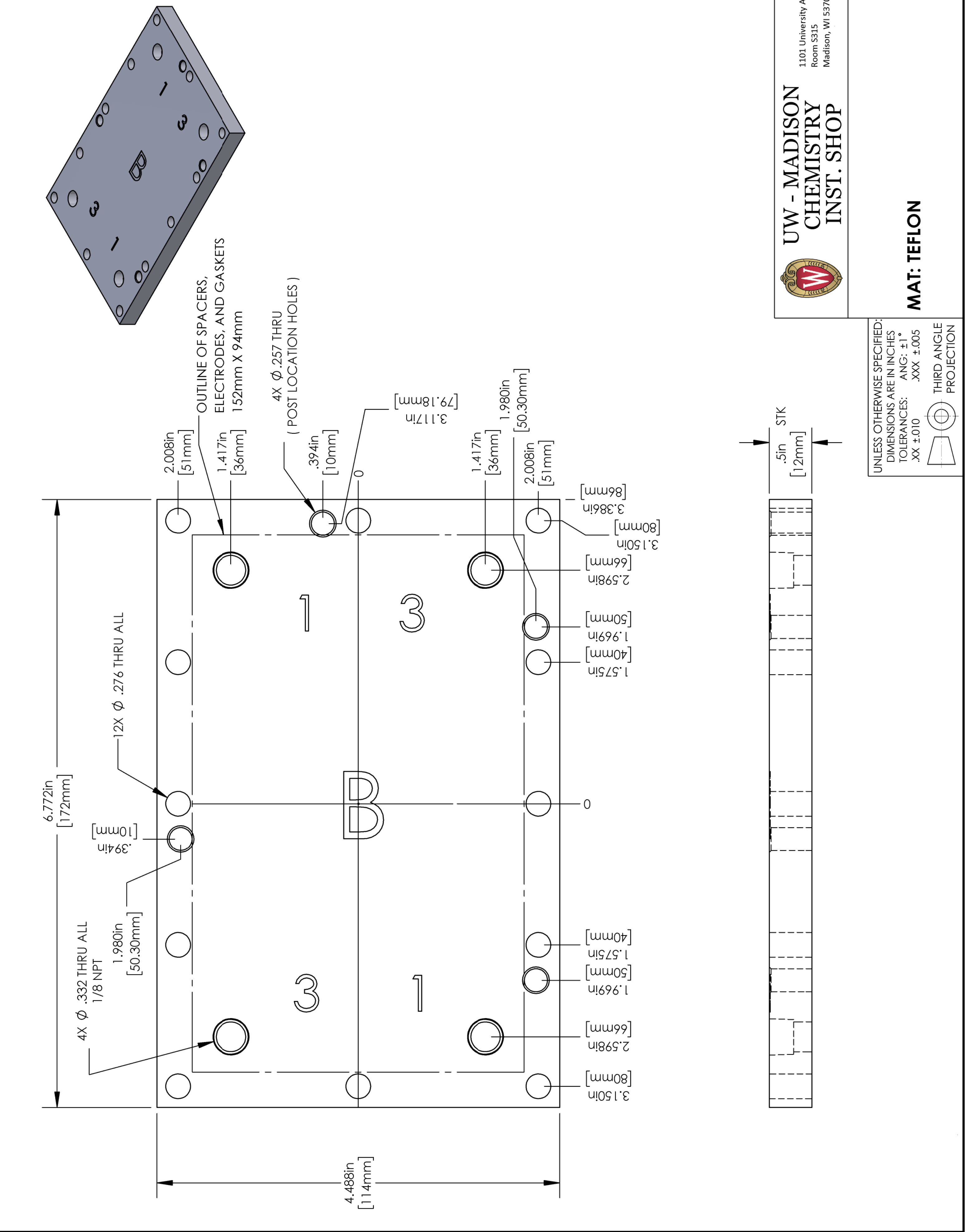

Small (4 cm^2) Cell Clamp

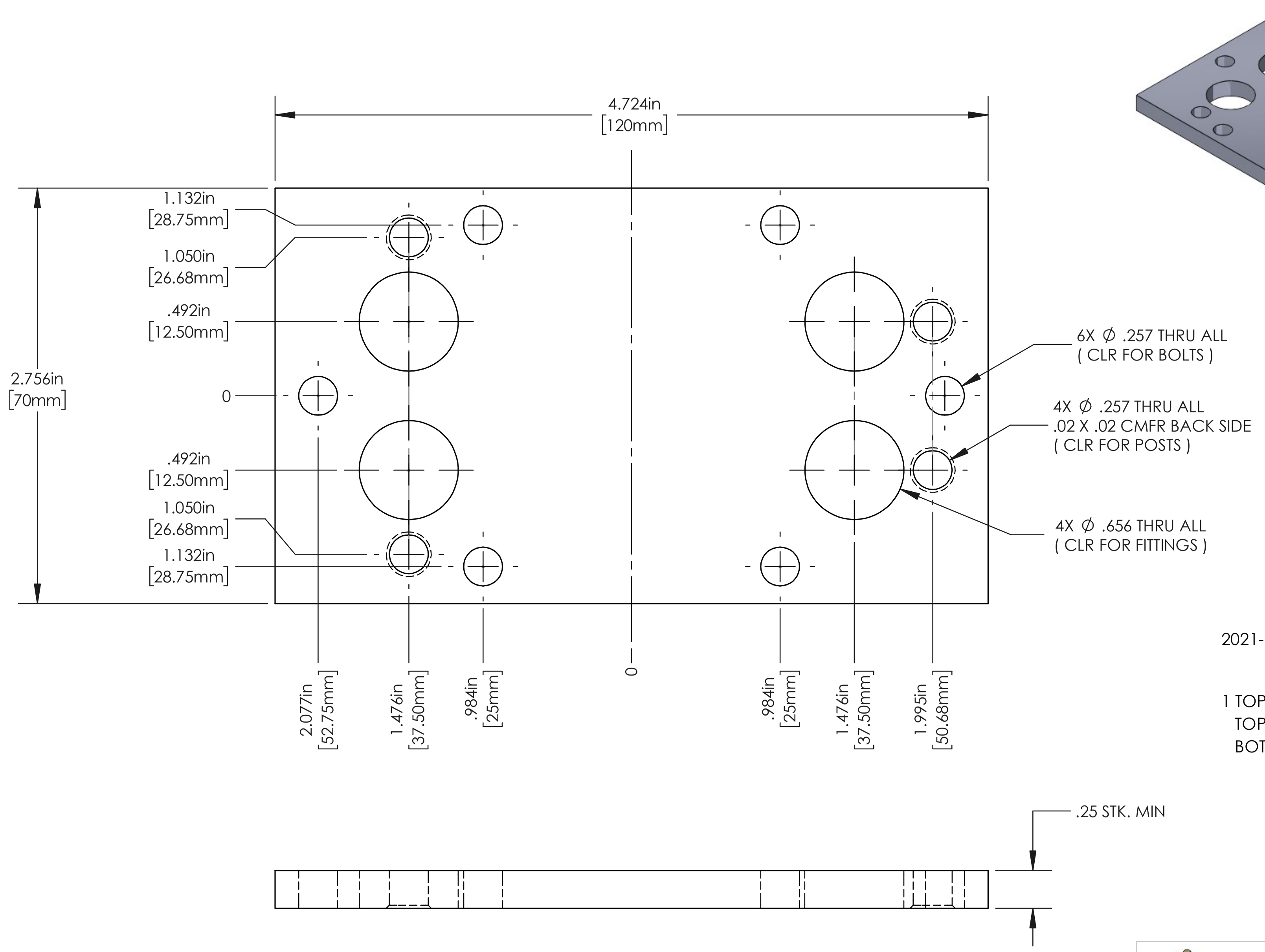

2021-11-10 REQUEST - 1 SET

1 TOP AND 1 BOTTOM REQ'D PER SET
TOP AS DRAWN
BOTTOM OMIT FITTING AND POST HOLES

UW - MADISON
CHEMISTRY
INST. SHOP

1101 University Ave
Room S315
Madison, WI 53706

UNLESS OTHERWISE SPECIFIED:
DIMENSIONS ARE IN INCHES
TOLERANCES: ANG: ±1°
.XX ±.010 .XXX ±.005
THIRD ANGLE PROJECTION

MATL: STAINLESS STEEL

Small (4 cm^2) Cell "A" Plate

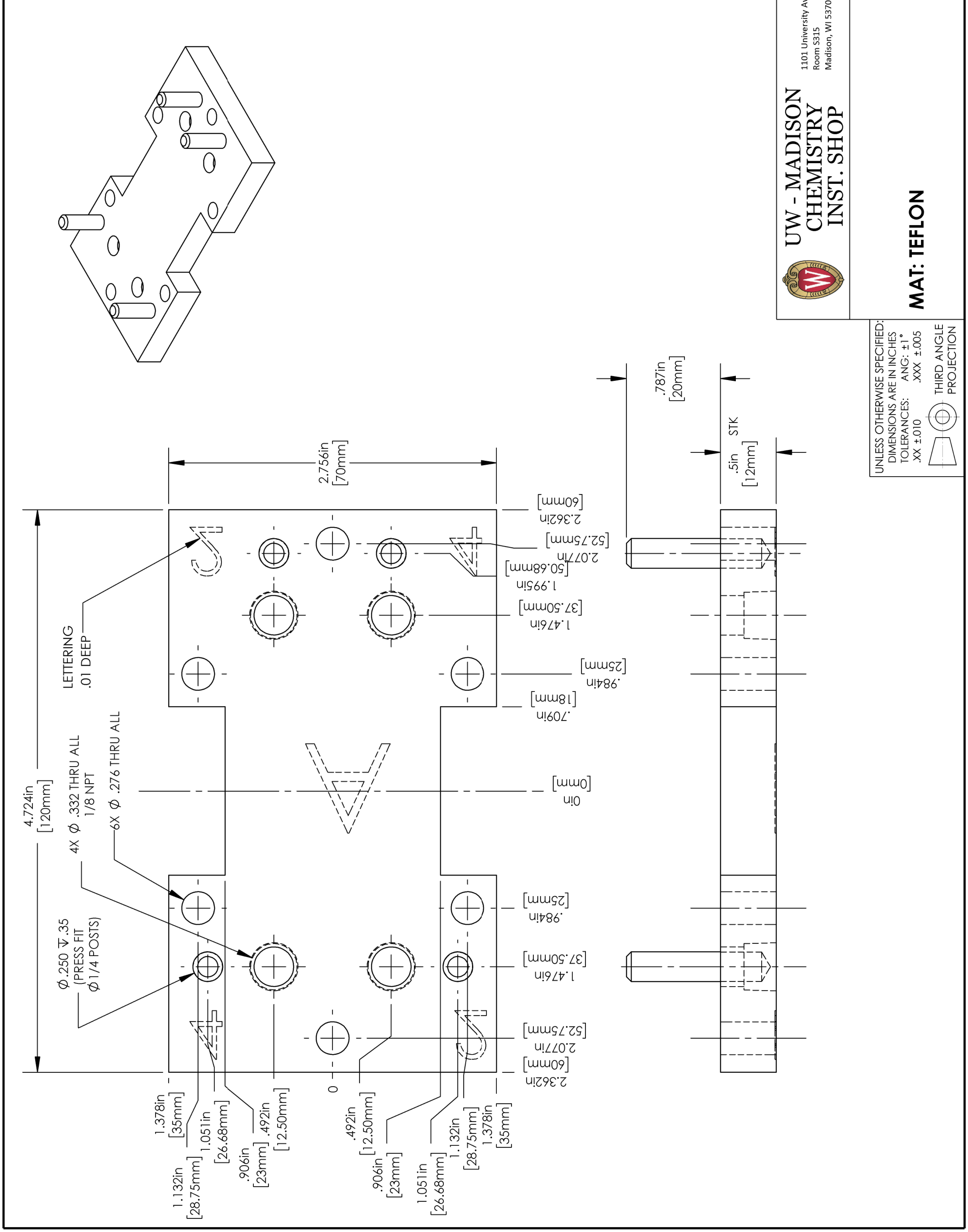

Small (4 cm^2) Cell Current Collector / Zn Anode
***Increase thickness as desired for Zn Anode**

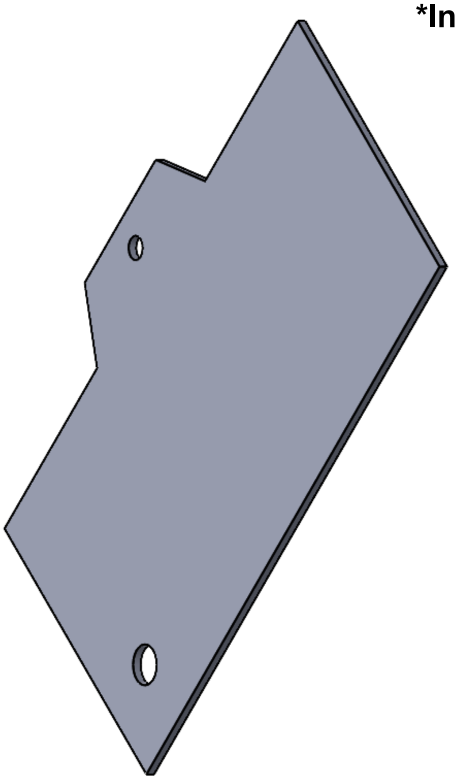

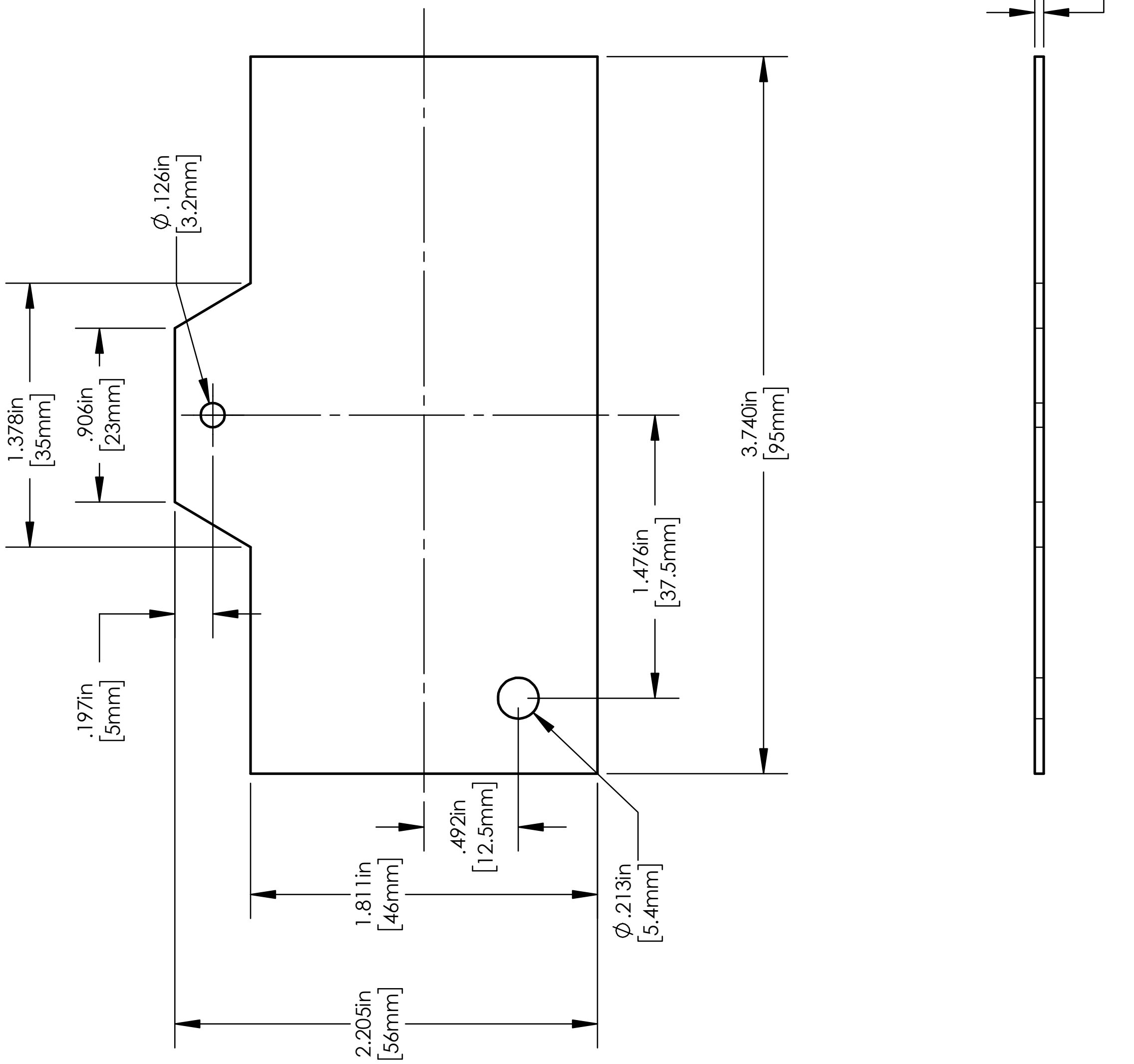

UW - MADISON
CHEMISTRY
INST. SHOP
1101 University Ave
Room S315
Madison, WI 53706

MAT: .048" STAINLESS STEEL

UNLESS OTHERWISE SPECIFIED:
DIMENSIONS ARE IN INCHES
TOLERANCES: ANG: ±1°
.XX ±.010 .XXX ±.005

THIRD ANGLE
PROJECTION

Small (4 cm^2) Cell C Felt Frame

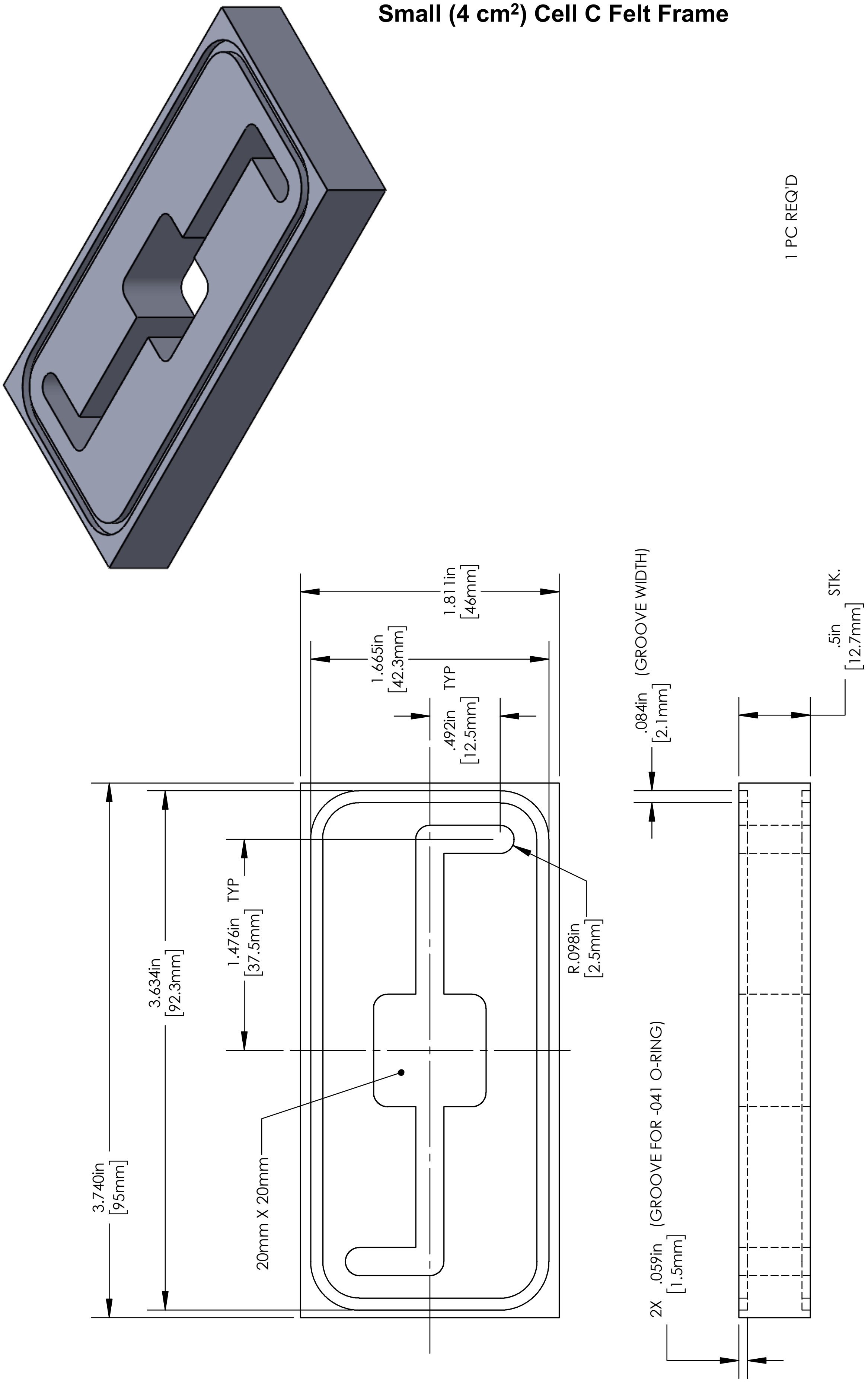

UW - MADISON
CHEMISTRY
INST. SHOP

1101 University Ave
Room 5315
Madison, WI 53706

MAT: HDPE

UNLESS OTHERWISE SPECIFIED:
DIMENSIONS ARE IN INCHES
TOLERANCES: ANG: ±1°
.XX ±.010 .XXX ±.005

THIRD ANGLE PROJECTION

Small (4 cm^2) Cell "B" Plate

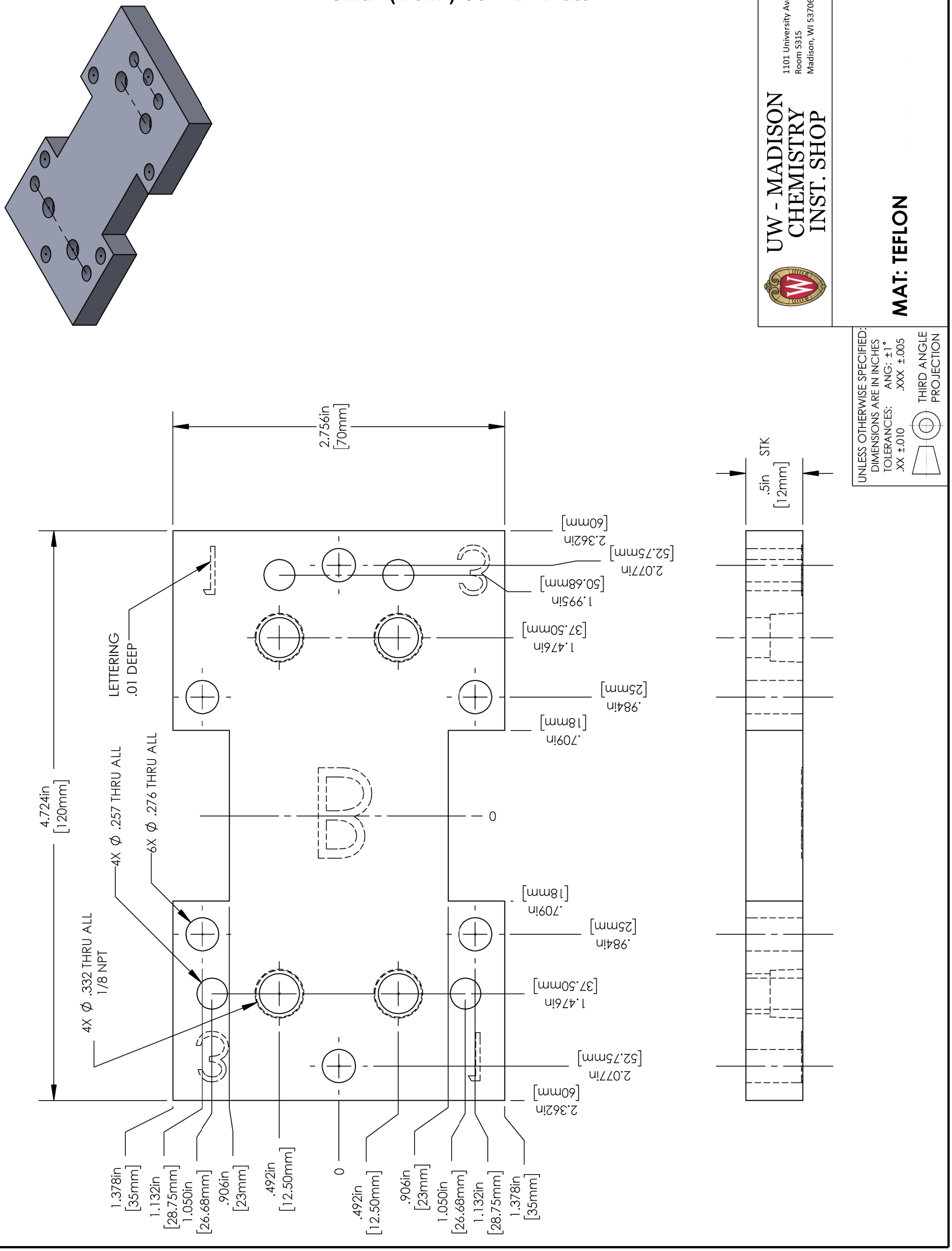

J2205

Large (841 cm^2) Cell Assembly

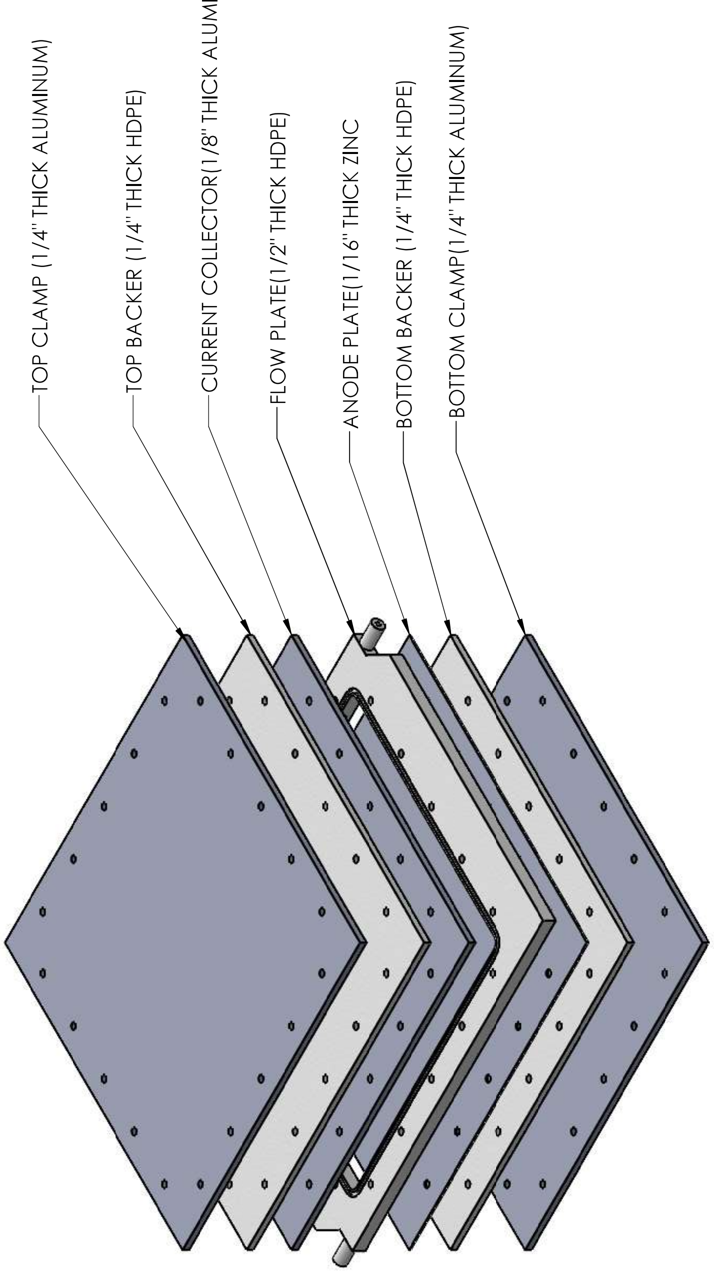

UW - MADISON
CHEMISTRY
INST. SHOP
1101 University Ave
Room S315
Madison, WI 53706

UNLESS OTHERWISE SPECIFIED:
DIMENSIONS ARE IN INCHES
TOLERANCES: ANG: ±1°
.XX ±.010 .XXX ±.005
THIRD ANGLE PROJECTION

Large (841 cm²) Cell Flow Plate

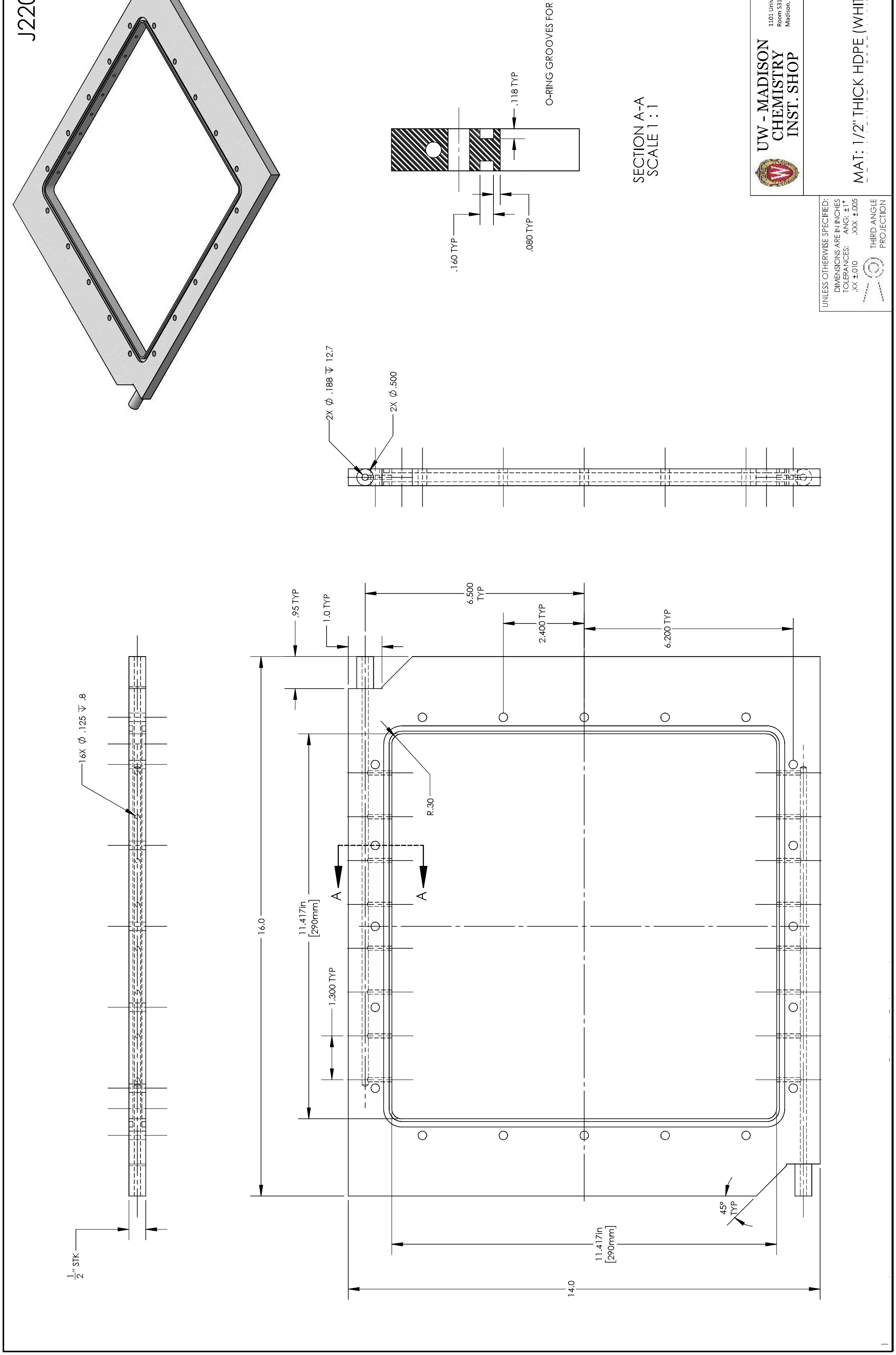

J2205

Large (841 cm^2) Cell Current Collector / Zn Anode / Clamp
***For Zn anode and clamp remove 5x #4-40 UNC THRU ALL**

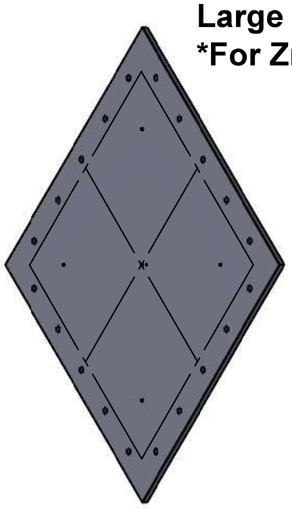

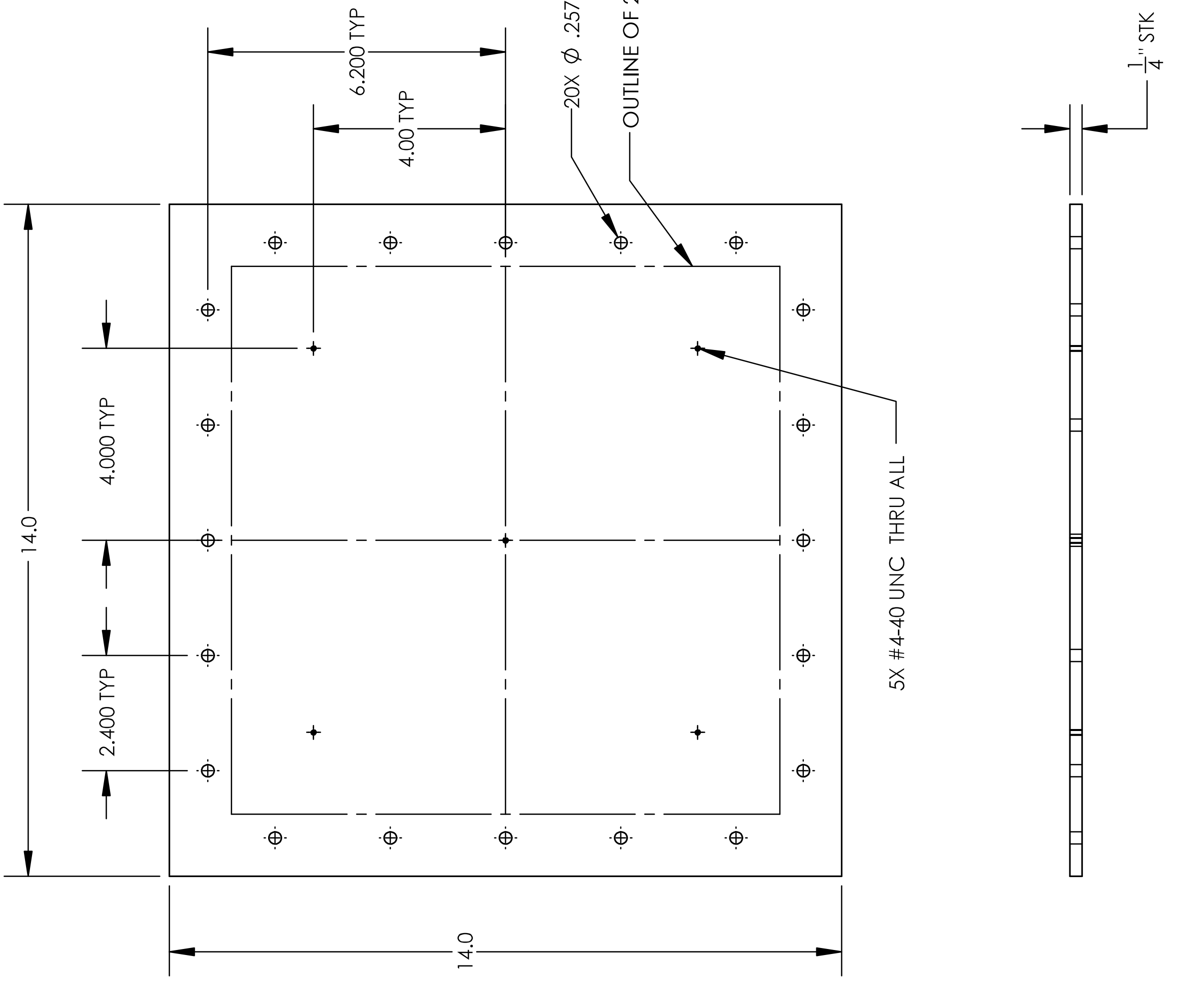

UW - MADISON
CHEMISTRY
INST. SHOP
1101 University Ave
Room S315
Madison, WI 53706

MAT: 1/4" THICK ALUMINUM

UNLESS OTHERWISE SPECIFIED:
DIMENSIONS ARE IN INCHES
TOLERANCES: ANG: ±1°
.XX ±.010 .XXX ±.005
THIRD ANGLE PROJECTION

Plastic Cap for C Felt

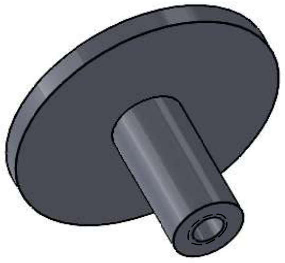

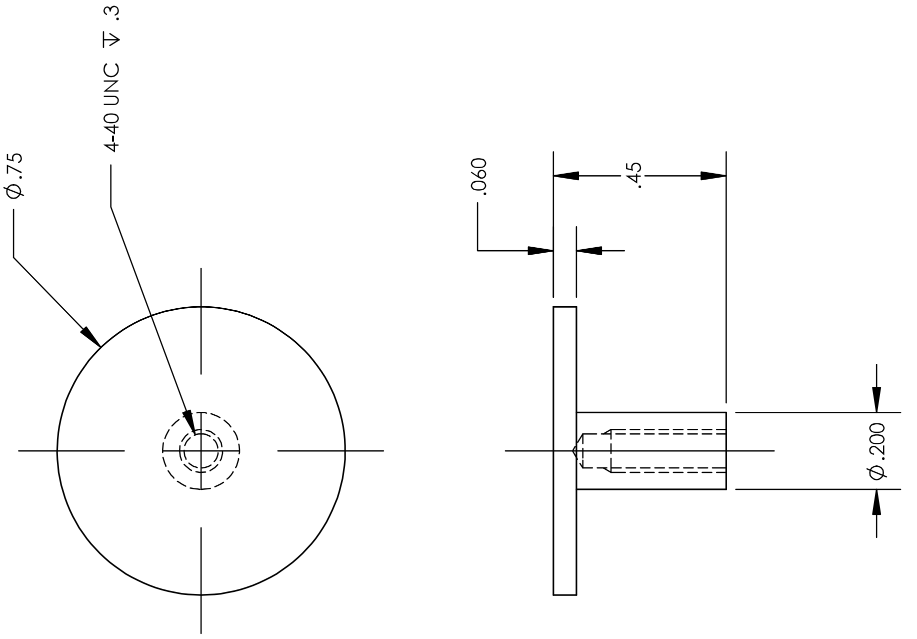

UW - MADISON
CHEMISTRY
INST. SHOP

1101 University Ave
Room S315
Madison, WI 53706

UNLESS OTHERWISE SPECIFIED:
DIMENSIONS ARE IN INCHES
TOLERANCES: ANG: ±1°
.XX ±.010 .XXX ±.005

THIRD ANGLE PROJECTION
